# The *Lethal(2)-Essential-for-Life* [*L(2)EFL*] Gene Family Modulates Dengue Virus Infection in *Aedes aegypti*

**DOI:** 10.3390/ijms21207520

**Published:** 2020-10-12

**Authors:** Lucky R. Runtuwene, Shuichi Kawashima, Victor D. Pijoh, Josef S. B. Tuda, Kyoko Hayashida, Junya Yamagishi, Chihiro Sugimoto, Shoko Nishiyama, Michihito Sasaki, Yasuko Orba, Hirofumi Sawa, Tomohiko Takasaki, Anthony A. James, Takashi Kobayashi, Yuki Eshita

**Affiliations:** 1 Department of Infectious Disease Control, Faculty of Medicine, Oita University, Oita 879-5593, Japan; takashik@oita-u.ac.jp; 2 Department of Computational Biology, Graduate School of Frontier Sciences, University of Tokyo, 5-1-5 Kashiwanoha, Kashiwa, Chiba 277-8562, Japan; 3 AIDS Research Centre, National Institute of Infectious Diseases, Tokyo 162-8640, Japan; 4 Database Center for Life Science, Joint Support-Center for Data Science Research, Research Organization of Information and Systems, 178-4-4 Wakashiba, Kashiwa, Chiba 277-0871, Japan; kwsm@dbcls.roia.ac.jp; 5 Faculty of Medicine, Sam Ratulangi University, Kampus Unsrat, Bahu Manado 95-115, Indonesia; victorpijoh@gmail.com (V.D.P.); jsbtuda@yahoo.com (J.S.B.T.); 6 Division of Collaboration and Education, Research Center for Zoonosis Control, Hokkaido University, North 20, West 10 Kita-ku, Sapporo, Hokkaido 001-0020, Japan; kyouko-h@czc.hokudai.ac.jp (K.H.); junya@czc.hokudai.ac.jp (J.Y.); sugimoto@czc.hokudai.ac.jp (C.S.); 7 Global Station for Zoonosis Control, GI-CoRE, Hokkaido University, North 20, West 10 Kita-ku, Sapporo, Hokkaido 001-0020, Japan; 8 Laboratory of Zoonotic Diseases, Faculty of Applied Biological Sciences, Gifu University, 1-1 Yanagido, Gifu-shi, Gifu 501-1193, Japan; shnishiy@gifu-u.ac.jp; 9 Division of Molecular Pathobiology, Research Center for Zoonosis Control, Hokkaido University, North 20, West 10 Kita-ku, Sapporo, Hokkaido 001-0020, Japan; m-sasaki@czc.hokudai.ac.jp (M.S.); orbay@czc.hokudai.ac.jp (Y.O.); h-sawa@czc.hokudai.ac.jp (H.S.); 10 National Institute of Infectious Diseases, Tokyo 162-8640, Japan; dengue3124@yahoo.co.jp; 11 Kanagawa Prefectural Institute of Public Health, Kanagawa 253-0087, Japan; 12 Departments of Microbiology & Molecular Genetics and Molecular Biology & Biochemistry, University of California, Irvine, CA 92697, USA; aajames@uci.edu; 13 Departments of Medical Entomology, Faculty of Tropical Medicine, Mahidol University, 420/6 Ratchawithi Road, Rajathewi, Bangkok 10400, Thailand; 14 Research Institute for Microbial Diseases, Osaka University, 3-1 Yamadaoka, Suita, Osaka 565-0871, Japan; 15 Hokudai Center for Zoonosis Control in Zambia, Research Center for Zoonosis Control, Hokkaido University, North 20, West 10 Kita-ku, Sapporo, Hokkaido 001-0020, Japan

**Keywords:** lethal(2)-essential-for-life, dengue, *Aedes aegypti*

## Abstract

Efforts to determine the mosquito genes that affect dengue virus replication have identified a number of candidates that positively or negatively modify amplification in the invertebrate host. We used deep sequencing to compare the differential transcript abundances in *Aedes aegypti* 14 days post dengue infection to those of uninfected *A. aegypti*. The gene *lethal(2)-essential-for-life* [*l(2)efl*], which encodes a member of the heat shock 20 protein (HSP20) family, was upregulated following dengue virus type 2 (DENV-2) infection in vivo. The transcripts of this gene did not exhibit differential accumulation in mosquitoes exposed to insecticides or pollutants. The induction and overexpression of *l(2)efl* gene products using poly(I:C) resulted in decreased DENV-2 replication in the cell line. In contrast, the RNAi-mediated suppression of *l(2)efl* gene products resulted in enhanced DENV-2 replication, but this enhancement occurred only if multiple *l(2)efl* genes were suppressed. *l(2)efl* homologs induce the phosphorylation of eukaryotic initiation factor 2α (eIF2α) in the fruit fly *Drosophila melanogaster*, and we confirmed this finding in the cell line. However, the mechanism by which *l(2)efl* phosphorylates eIF2α remains unclear. We conclude that *l(2)efl* encodes a potential anti-dengue protein in the vector mosquito.

## 1. Introduction

*Aedes aegypti* is an important vector of human disease pathogens, and it transmits dengue viruses that cause a spectrum of diseases that are of increasing concern as emerging infections [1,2]. The vector mosquito is hematophagous and usually becomes infected by dengue virus while feeding on the blood of infected individuals. After proliferation in the midgut and dispersal through the mosquito body to the salivary glands, the virus is transmitted to uninfected persons during subsequent blood feedings [3]. Breaking the transmission cycle is important, as effective vaccines and specific preventative and therapeutic drugs are not yet available [4,5,6]. The only approved dengue vaccine has been reviewed as unsafe for seronegative individuals at the time of vaccination [7], which is a drawback in dengue prevention. Traditional methods to control transmission include the avoidance of mosquito bites using repellents and suppressing mosquito populations with insecticides and breeding site removal [2]. However, insecticide resistance and the ineffectiveness of community-based source reduction make it impossible to sustain breaks in transmission [8]. The development of transgenic mosquitoes for either population suppression (genetic analog to an insecticide) or population modification (rendering the mosquito genetically incapable of transmitting the viruses) are among the proposals for novel control methods [9,10].

Scientists have tried to elaborate the complex relationships between dengue viruses and their vectors. Experiments using next-generation sequencing to perform a comprehensive analysis of gene expression following dengue infection have been carried out [11,12]. Researchers have predicted protein interactions between dengue viruses and *A. aegypti* using computer models [13,14]. Protein profiles of dengue-infected *A. aegypti* also have been published [15]. The mosquito transcriptome profiles in response to dengue infection have been studied using microarrays in cells [16], dissected organs [17,18], or whole-body preparations [11]. Specific genes, such as those related to immunity or innate immune pathways also have been investigated [19,20].

We used deep sequencing to analyze the differential transcript abundances between dengue-infected and uninfected *A. aegypti.* In contrast to previous studies that used young mice or viruses cultured in cells, we used adult immunocompetent mice for the challenge assay [21]. We infected the Liverpool strain of *A. aegypti*, for which the complete genome sequence is available [22], with dengue virus type 2 (DENV-2) Southeast Asian type, an isolate that has the potential to cause severe disease [23,24]. We identified a family of genes, including *lethal(2)-essential-for-life* [*l(2)efl*], which encodes a product belonging to the heat shock protein 20 (HSP20) family and whose transcripts are more abundant in infected mosquitoes than in uninfected controls. The induction or inhibition of the gene and family members in vitro results in decreased and increased DENV-2 replication, respectively.

## 2. Results and Discussion

### 2.1. Mosquito Infection with DENV-2 Using Direct Feeding

**Figure 1:**
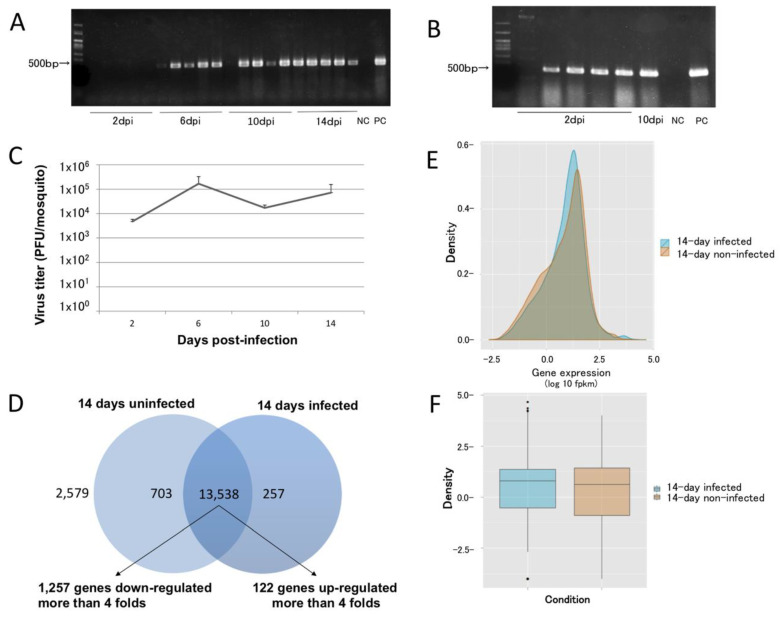
Temporal, spatial, and quantitative dynamics of dengue virus dissemination in *Aedes aegypti*. (**A**) Dengue virus (DENV) dissemination at 2, 6, 8, and 10 days post-infection (dpi) (N = 5 for each time point). (**B**) At 2 dpi, infected mosquitoes had DENV in their carcasses. One mosquito was not infected, and one 10 dpi mosquito had an apparent midgut escape barrier that prevented DENV dissemination. (**C**) The DENV titer in infected mosquitoes was stable for up to 14 dpi (N = 3). (**D**) As many as 13,538 genes were affected by DENV infection at 14 dpi, with 1257 and 122 genes showing decreased and increased transcript abundance, respectively, by more than 4-fold. As many as 2579 genes had products that showed no differential abundance. (**E**,**F**) Density and box plots of the overlapped transcript accumulation between 14-day-infected and uninfected mosquitoes. NC: negative control; PC: positive control.

Mosquitoes were infected by allowing them to feed to repletion on adult immunocompetent mice infected with DENV-2, as previously reported [21]. We used 5- to 9-day-old mosquitoes to increase the blood feeding rate. At this age, the mosquitoes had reached the second stage of ovarian development and would physiologically seek blood. We also performed the feeding in the late afternoon to coincide with the maximum circadian feeding activity. RT-PCR screening detected the DENV-2 genome in samples of mosquito legs collected at 6, 10, and 14 dpi, but not in 2 dpi mosquitoes (Figure 1A). These results are consistent with previous reports that demonstrated that the DENV-2 antigen is detected in the *A. aegypti* Chetumal strain in the trachea and salivary glands at 2 and 4 dpi, respectively [25]. However, the dissemination timing varies among mosquito strains, as salivary gland infection of Rex-D mosquitoes was not observed until 10 dpi. One of five mosquitoes at 10 dpi in the present study did not show any viral genome in its legs, although the RT-PCR of its carcass showed the presence of the DENV-2 genome (Figure 1B), thereby presenting evidence of a midgut barrier in this specific mosquito strain. Furthermore, one mosquito was not infected with the dengue virus, as shown by the absence of the DENV-2 genome in its carcass. As many as 99.6-100% of the female mosquitoes in each cage clearly fed on infected mice. Therefore, we did not remove the nonfeeding mosquitoes due to safety reasons. Moreover, we found that the DENV-2 kinetics in *A. aegypti* were stable for 2 to 14 days (Figure 1C).

### 2.2. Sequencing Data

A total of 131,774,320 reads were mapped to the reference genome using Bowtie v2.2 and TopHat v2.1, with an efficiency of 90.5% and 95.1% for the infected and uninfected groups, respectively (Table 1). It is possible that a few uninfected mosquitoes were included in the infected population, but the fraction (≤1/20 as determined above) was anticipated to be too small to affect the results. The differential transcript abundance of 10 selected loci (histone H4 [AAEL003689], *lethal(2)-essential-for-life* [AAEL013338], 4-nitrophenyl phosphatase [AAEL007097], cdc6 [AAEL010855], cactus [AAEL000709], antibacterial peptide [AAEL004233], hypothetical protein [AAEL004851], vago [AAEL000200], zinc carboxypeptidase [AAEL001863], carboxypeptidase [AAEL010766]) identified by RNA-seq were confirmed by qRT-PCR. These 10 loci exhibited a Spearman Rho’s R of 0.79394 (*p* <0.01) between the two assays (Table 2).

### 2.3. Identification of L(2)efl

Cufflinks processing showed that the transcript abundance of 13,538 genes (~79.3% of annotated genes) varied between the samples (Figure 1D, Supplementary Tables S4 and S5) with 6,704 and 6,834 genes with transcripts that increased or decreased, respectively. A total of 122/6,704 and 1,257/6,834 of the genes had >4-fold increases or decreases, respectively. Moreover, 703 and 257 transcripts were found exclusively in uninfected or infected mosquitoes, respectively. As many as 2579 genes did not show any differential transcript accumulation. Most of the genes had overlapping expression patterns, with a trend towards upregulation between the two groups (Figure 1E,F). The majority of genes whose transcripts increased in abundance in infected mosquitos were annotated by Gene Ontology [26] as binding and catalytic activity proteins. Genes exhibiting decreases were annotated as having catalytic activity (Supplementary Tables S4 and S5). Collectively, histone protein-encoding loci represented 29.61% of the genes, with the transcript abundance increasing ≥4-fold in infected mosquitoes. This observation is consistent with the fact that the DENV capsid protein has been shown to target the four core histones (H2A, H2B, H3, and H4) in liver cells [27], which are replaced by increasing their transcription.

Genes encoding several members of the heat shock protein 20 (HSP20) family also showed modulations in transcript accumulation. HSPs are known to be induced during stress (including heat shock, pathogen infection, heavy metal ion exposure, hypoxia, and osmotic stress), and they act as chaperones to guide misfolded proteins [28]. The *A. aegypti* genome includes 19 genes with the HSP20 domain. We detected increased differential transcript expression in infected mosquitoes for nine of these genes, including the *lethal(2)-essential-for-life* [*l(2)efl*] locus (Table 3).

### 2.4. DENV-2 Replication 

**Figure 2:**
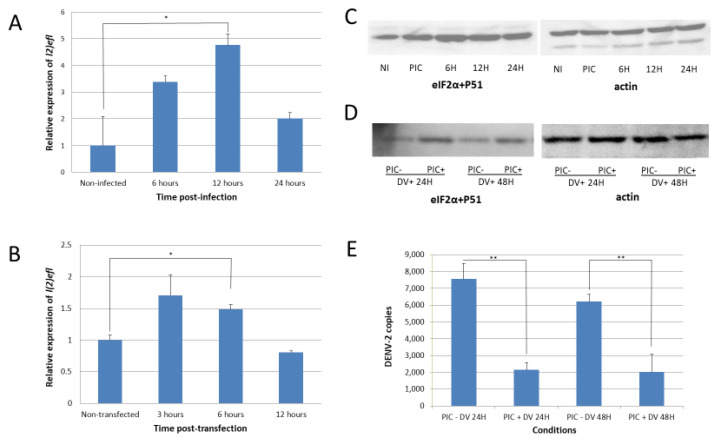
Dengue-virus infection increases the *l(2)efl* expression and phosphorylates eIF2α in CCL-125 cells. (**A**) When infected with dengue virus (DENV), *l(2)efl* was upregulated as high as ~5-fold, with a peak at 12 h post infection (hpi). (**B**) The poly(I:C) synthetic dsRNA also upregulated the *l(2)efl* expression as high as ~1.6-fold, with a peak at 3 hpi. (**C**) DENV infection caused the phosphorylation of eukaryotic translation initiation factor 2, subunit 1 alpha (eIF2α). (**D**) Transfection with poly(I:C) and subsequent infection DENV resulted in higher phosphorylation levels up to 48 hpi. (**E**) Induction of *l(2)efl* by poly(I:C) and the phosphorylation of eIF2α reduced the DENV type 2 replication at 24 and 48 hpi. All the experiments were performed using three biological replicates (except for Figure D, which had two biological replicates). NI: noninfected; PIC: poly(I:C); DV: dengue virus. * *p* < 0.05, ** *p* < 0.01.

We infected the *A. aegypti* CCL-125 cell line with DENV-2 at an MOI of 5.0. Consistent with the RNA-seq data, the *l(2)efl* (AAEL013338) expression analyzed by RT-PCR was increased by approximately 3.25-fold by DENV-2 infection at 6 h post infection and reached a peak of approximately 4.75-fold at 12 h post infection (Figure 2A). Poly(I:C), a synthetic dsRNA, transfection into CCL-125 cells also induced the expression of *l(2)efl*, with the transcript abundance reaching a peak (~1.6-fold) at 3 h post transfection (Figure 2B). The synthetic agent did not achieve the highest level induced by DENV-2.

*l(2)efl* overexpression is known to result in the phosphorylation of eukaryotic translation initiation factor 2, subunit 1 alpha (eIF2α) in the fruit fly *Drosophila melanogaster* [29]. We observed a similar result in *A. aegypti* following DENV-2 infection, as eIF2α was phosphorylated at serine position 51 (Figure 2C). The introduction of poly(I:C) also enhanced phosphorylation in cells infected with DENV-2 (Figure 2D). The eIF2α phosphorylation may decrease protein synthesis and may also repress DENV-2 replication. We confirmed this predicted correlation and observed that the *l(2)efl* overexpression suppressed the DENV-2 replication by approximately 10-fold in poly(I:C)-treated CCL-125 cells at 48 h post infection (hpi) (Figure 2E).

### 2.5. Suppression of L(2)efl-1 and L(2)efl-4 Enhances DENV-2 Replication 

**Figure 3:**
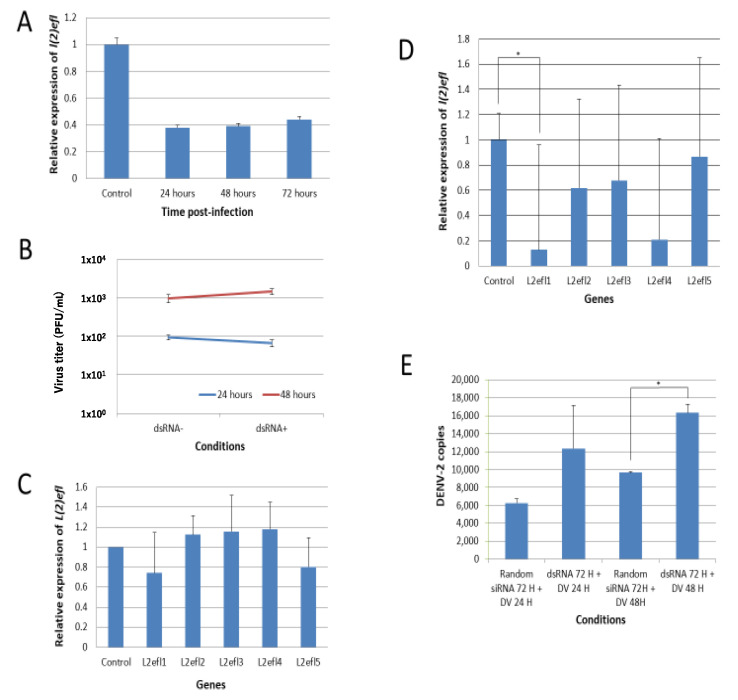
Suppression of *l(2)efl-1* and *l(2)efl-4* promotes dengue virus type 2 replication in CCL-125 cells. (**A**) Gene-specific dsRNAs ablated 60% of *l(2)efl* transcripts for as long as 72 h post transfection. (**B**) Suppression did not cause any difference in the dengue virus type 2 (DENV2) replication 24 and 48 h post infection, which may have been due to compensation by other *l(2)efl* homologs (**C**). (**D**) RNAi effectively repressed *l(2)efl-1* and *l(2)efl-4* transcripts, but only partially affected *l(2)efl-2* and *l(2)efl-3*. (**E**) *l(2)efl-1* and *l(2)efl-4* transcript ablation enhanced the DENV2 replication by as much as 2-fold in CCL-125 cells. All the experiments were performed using three biological replicates (except for Figures B and C, which have two biological replicates). Rdm: random siRNA; dsRNA: double-stranded RNA; DV: dengue virus; H: hours. * *p* < 0.05.

The introduction of *l(2)efl-1* dsRNAs into CCL-125 cells resulted in a 60% reduction in the transcript abundance up to 72 h post dsRNA-transfection (Figure 3A). However, the reduction in specific transcripts did not lead to any difference in DENV-2 titer at 24 and 48 hpi (Figure 3B).

We hypothesized that the failure to observe an impact on the viral titer in the previous experiments may have resulted from compensation by other *l(2)efl* family members. Our RNA-seq data showed that there were multiple upregulated *l(2)efl* homologs (Table 3). We randomly checked another four *l(2)efl* homologs and found that, after the introduction of *l(2)efl-*1 dsRNA, *l(2)efl-*2, 3, and 4 were induced but not *l(2)efl*-5 (Figure 3C). This induction may have compensated for the *l(2)efl-*1 loss of action. Therefore, we suppressed the expression of *l(2)efl*-1 to *l(2)efl*-4. dsRNA targeting these gene products effectively reduced the *l(2)efl*-1 and *l(2)efl-*4 transcripts, but it only partially reduced the abundance of *l(2)efl-*2 and *l(2)efl-*3 (Figure 3D). *l(2)efl-*1- and *l(2)efl-*4-suppressed CCL-125 cells infected with DENV-2 had higher DENV-2 copies at 24 and 48 hpi (Figure 3E). We conclude that *l(2)efl* helps to suppress the DENV-2 replication in *A. aegypti*, but that one *l(2)efl* homolog alone is not enough to suppress virus replication and function.

## 3. Material and Methods

### 3.1. Mosquito Rearing

The *Aedes aegypti* LVP INB strain was grown at 26 ± 1 °C and at 80–95% relative humidity, with a 16:8 h light:dark cycle. Larvae were fed on a combination of finely ground mouse food (CE-2, CLEA, Shizuoka, Japan), dried yeast EBIOS (#2332001X1084, Asahi Food and Healthcare, Shibuya, Tokyo, Japan), and finely ground fish food (TetraMin, Spectrum Brands Japan, Yokohama, Kanagawa, Japan). Male and female adults were housed together in a cage with unlimited access to a 4% sugar solution until infection. The mosquitoes used in the experiments were 5–9 days post eclosion. Females were not blood fed prior to the experiments.

### 3.2. Mosquito Infection

The stock virus of DEN-2 ThNH7/93 was prepared by infecting C6/36 mosquito cells. Mosquitoes were infected with dengue virus as previously described (Runtuwene et al. 2014). Briefly, K562 erythroleukemia cells were infected with 1.5 × 10^7^ PFU/mL DEN-2 ThNH7/93 (MOI 1.0) and then the cells were supplemented with D4-I-1D6 monoclonal antibody. The virus concentration in the mouse blood was maintained at 10^4^ to 10^5^ PFU/mL for at least up to 7 h post injection. Five- to nine-day-old female mosquitoes were allowed to blood-feed from the infected mice 5 h post-injection [21]. After blood feeding, the mosquitoes were incubated at 28 °C for 2, 6, 10, and 14 days. Both the infected group and the noninfected group were provided unlimited access to a cotton pad impregnated with a 4% sugar solution. All the mosquitoes completely digested the blood meal by 14 days post blood feeding. The mosquitoes were frozen for at least 1 h at −80 °C and stored at −80° C until processing.

### 3.3. Confirmation of Mosquito Infection

Successful infection was confirmed using reverse transcriptase polymerase chain reaction (RT-PCR) with SuperScript III One-Step RT-PCR (Invitrogen, Carlsbad, CA, USA) and previously published universal DENV primers [30]. The DENV-2 titer in mosquitoes were measured with the plaque assay described below. The dissemination rate was determined by checking isolated mosquito legs for the DENV-2 genome. A positive sample indicated that the mosquito did not have midgut-restricted infection.

### 3.4. RNA Extraction and Illumina Library Preparation 

Total RNA was extracted using a combination of ISOGEN (Nippon Gene, Tokyo, Japan) and TruSeq Stranded mRNA Sample Preparation (Illumina, San Diego, CA, USA) from control mosquitoes and those 14 days post-infection (dpi) to DENV-2. Each group consisted of two pools of 25 females. A total of four pools (i.e., two from each group) were prepared as four single-read Illumina libraries according to the manufacturer’s protocol. After library validation with a bioanalyzer, the ds-cDNA was run for 40 cycles on an Illumina Genome Analyzer.

### 3.5. RNA-seq Data Analyses

The assembled supercontig sequences (version AaegL3) of the *A. aegypti* genome and the GTF annotation file (ver. AaegL3.3) were downloaded from VectorBase [31]. The supercontigs were used as the reference genome sequence. Sequence reads were mapped to the reference genome using Bowtie v2.2 and then TopHat v2.1 [32] with the GTF file, a segment length of 12, and a segment mismatch of 1. The Cuffdiff program within the package Cufflinks v2.2.1 [33] was used to calculate the reads per kilobase per million reads (RPKM) of each gene and to identify genes with significant transcript abundance differences between two samples with a false discovery rate (FDR) of 0.01%. The entire primary sequencing data are archived in the DDBJ Sequence Read Archive (DRA) as accession number DRA002522. 

### 3.6. Transfection and Infection of the A. aegypti Cell Line CCL-125 with Poly(I:C) and DENV-2

The *A. aegypti* CCL-125 (ATC-10) cell line (purchased from American Type Culture Collection, Manassas, VA, USA) was transfected with 30 µg/mL poly(I:C) (Sigma-Aldrich, St. Louis, MO, USA) using polyethylenimine (PEI, Sigma-Aldrich, St. Louis, MO, USA) at a working concentration of 3 µg/mL. Prior to transfection, the cells were washed and incubated with serum-free minimal essential medium (MEM, Sigma-Aldrich, St. Louis, MO, USA) to cause serum starvation. The transfected cells were incubated at 28 °C with 5% CO_2_ for 3, 6, and 12 h before harvesting. CCL-125 cells were also infected with the ThNH7/93 DENV-2 strain at a multiplicity of infection (MOI) of 5.0, followed by incubation at 28 °C and 5% CO_2_ for one hour. The cells were washed to remove unbound virus particles and then incubated for 6, 12, and 24 h in MEM containing 2% fetal bovine serum (FBS, HyClone, Characterized Grade, Canadian Origin, Cytiva, Marlborough, MA, USA) before collection. To analyze the effect of the *l(2)efl* upregulation on DENV-2 replication, the CCL-125 cells were transfected with poly(I:C) and incubated for 1.5 h. The cells were then infected with the ThNH7/93 DENV-2 strain at an MOI of 5.0 and incubated for 24 and 48 h before cell collection.

### 3.7. Immunoblotting of EIF2α

Poly(I:C)-treated or DENV-2-infected CCL-125 cells were collected and lysed using lysis buffer (1 mM of DTT and 1× protease inhibitor in HEPES-NaCl-Glycerol-Triton X 100 (HNTG) buffer). The cell lysates were run on a 10% polyacrylamide gel. The proteins were transferred to a membrane (Millipore Immobilon-P, Merck Millipore, Burlington, MA, USA), which was incubated with anti-eIF2α (phospho S51) primary antibody (Abcam, Cambridge, UK) for 12 h. The peroxidase-conjugated mouse anti-rabbit IgG (Jackson Laboratory, Bar Harbor, ME, USA) secondary antibody was reacted with horseradish peroxidase (HRP) substrate (Merck Millipore, Burlington, MA, USA) Actin antibody (Jackson Laboratory, Bar Harbor, ME, USA) was used as a loading control.

### 3.8. L(2)efl Suppression

Four dsRNAs with sequences complementary to *l(2)efl-1*, *l(2)efl-2*, *l(2)efl-3*, and *l(2)efl-4* (Supplementary Tables S1–S3) were synthesized using the MegaScript RNAi Kit (Ambion, Austin, TX, USA). Suppression was achieved by introducing 1 µg of dsRNA directly to confluent CCL-125 cells. The cells were then incubated at 28 °C and 5% CO_2_ for 24, 48, and 72 h. Control samples were transfected with MISSION siRNA Universal Negative Controls (Sigma-Aldrich, St. Louis, MO, USA). To analyze the effect of *l(2)efl* suppression on DENV-2 replication, CCL-125 cells were transfected with *l(2)efl-1*, *l(2)efl-2*, *l(2)efl-3*, and *l(2)efl-4* dsRNAs and incubated for 24 h prior to infection with the ThNH7/93 DENV-2 strain at an MOI of 5.0. The cells were incubated for 24 and 48 h before harvesting.

### 3.9. Virus Titer

Virus titer was measured with plaque assays. Briefly, mosquitoes or mosquito legs were homogenized in MEM containing 2% FBS or supernatants of DENV-2-infected CCL-125 cells were collected, diluted to 10 to 10^6^-fold, and introduced to each well of a 12-well Corning plate (Corning Inc., Corning, NY, USA) layered with a confluent Vero cell monolayer. Following 2 h of incubation at 37 °C and 5% CO_2_ with occasional shaking every 5–10 min, the infected monolayer was layered with methylcellulose containing 2% FBS and incubated again at 37 °C and 5% CO_2_ for 9 days. The overlay medium was removed, and the monolayer was fixed with formalin and stained with 1.25% methylene blue (Sigma-Aldrich, St. Louis, MO, USA). Plaques were counted, and the results were expressed as PFU per mosquito or PFU/mL.

### 3.10. Quantitative RT-PCR

A total of 10 genes identified by RNA-seq to be accumulated differentially between uninfected and infected mosquitoes were selected for real-time quantitative RT-PCR (qRT-PCR) analysis. Total RNA from a pool of either 10 uninfected or 10 infected females was extracted using a combination of TRIZOL reagent (Invitrogen, Carlsbad, CA, USA) and PureLink RNA Mini Kits (Ambion, Austin, TX, USA). qRT-PCR was performed using One Step SYBR PrimeScript PLUS (Takara Bio, Shiga, Japan) and a LightCycler 96 (Roche, Basel, Switzerland) (see Supplementary Tables S1–S3 for the primer sequences). Fold changes in transcript abundance between uninfected and infected mosquitoes were derived by the comparative CT method [34] using the *40srps17* gene (AAEL004715, which encodes the 40S ribosomal protein 17) as the reference. We compared the stability of three housekeeping genes (*40srps17* (AAEL004715), actin (AAEL004631), and *rpL5* (gi|94468377)) and found that *40srps17* was the most stable among them (Supplementary Figure S1). The correlation between the abundance values detected by RNA-seq and qRT-PCR for the 10 genes tested was estimated by calculating the Spearman Rho’s R using online software [35]. The *l(2)efl-1* to *l(2)efl-5* abundance levels were also measured after the collection of poly(I:C)-transfected and DENV-2-infected CCL-125 cells using the same procedure (see Supplementary Tables S1–S3 for primer sequences). DENV-2 copies were measured by absolute quantification that required two qRT-PCR steps. cDNAs were produced using the Transcriptor First-Strand cDNA Synthesis Kit (Roche) using random primers. Measurement with qRT-PCR was performed using the LightCycler FastStart DNA Master SYBRGreen I (Roche, Basel, Switzerland) and a LightCycler 96. The DENV-2 copy standard was produced by Nihon Gene Research Laboratory Inc. (Sendai, Miyagi, Japan). Statistical significance was calculated using a Student’s *t*-test.

### 3.11. Ethics Statement

The Committee for Animal Experiments at Oita University approved the experiments under permission numbered D00902. The Committee for Animal Experiments at Oita University complies with the Guidelines for Proper Conduct of Animal Experiments stipulated by the Science Council of Japan.

## 4. Conclusions

We observed complex changes in the *A. aegypti* transcriptome at 14 days after dengue virus infection. We identified at least one gene family—namely, *lethal(2)-essential-for-life*—to have a role in DENV-2 infection. Heat-shock proteins are induced during stress and act as chaperones to guide misfolded proteins [28]. The *l(2)efl* genes belong to the small HSP20 family, and while many family members are induced in the presence of stresses, *l(2)efl* transcription is upregulated by flavivirus infection [36], but not pollutants and insecticides [37]. When *A. aegypti* larvae are exposed to pollutants and insecticides as stressors, the *l(2)efl* gene is not upregulated [37], supporting the hypothesis that flaviviruses may be a selective upregulator of *l(2)efl* transcription. The overexpression of *l(2)efl* in *D. melanogaster* leads to the phosphorylation of eIF2α [29]. This phosphorylation inhibits the formation of translation initiation complexes, leading to translation inhibition, a mechanism that is postulated to impede virus protein translation [38,39]. *L(2)efl* may also work in *A. aegypti* through the same mechanism. However, it remains unclear how *l(2)efl* phosphorylates eIF2α, which is the limitation of our paper. We focused the analysis only on *l(2)efl* gene members. This analysis oversimplifies the complex interaction between proteins in the *A. aegypti*. Other decreased proteins, such as the Toll subunit in salivary glands, might play a bigger role in keeping a higher DENV-2 titer in the saliva [40]. We also only looked at the dynamics of DENV-2, which is another limitation of this paper.

## Figures and Tables

**Table 1:** RNA-seq generated more than 60 million reads in both groups. Among these reads, Bowtie v2.2 and TopHat v2.1 mapped at least 87.7% of the reads to the reference genome. At least one successfully aligned read was required for inclusion.

Sample Source	Replicate	Number of Reads Processed	Number of Reads with ≥1 Reported Alignment (Percent of Total Reads)	Number of Reads that Failed to Align (Percent of Total Reads)
**14 days noninfected**	1	39,174,903	37,360,756 (95.4%)	1,814,147 (4.6%)
	2	30,866,781	29,259,296 (94.8)	1,607,485 (5.2%)
**14 days infected**	1	27,119,695	23,790,556 (87.7%)	3,849,139 (12.3%)
	2	34,612,941	32,289,737 (93.3%)	2,323,204 (6.7%)

**Table 2:** Real-time PCR was used to validate the RNA-seq data. We randomly selected 10 genes and confirmed their expression in a pool of 10 mosquitoes. The RNA-seq and qRT-PCR data were obtained from two different mosquito populations. Compared to the RNA-seq data, the qRT-PCR data does not show large discrepancies in the abundances. The RNA-seq and qRT-PCR data are correlated statistically with a Spearman Rho’s R of 0.79394 (*p* < 0.01) between the two assays. qRT-PCR values are the average of two biological replicates.

Gene ID	Gene Annotation	RNA-seq			qRT-PCR	
		RPKM		Fold Changes	Normalized Abundance Values	
		Uninfected	Infected		Uninfected	Infected
AAEL003689	Histone H4	69.5104	999.832	14.38	1.00	1.80
AAEL013338	*Lethal(2)-essential-for-life*	743.143	7346.79	9.9	1.00	3.84
AAEL007097	4-nitrophenyl phosphatase	918.192	6941.7	7.56	1.00	2.55
AAEL010855	Cdc6	8.18748	59.1695	7.22	1.00	2.13
AAEL000709	Cactus	113.145	219.984	1.944	1.00	1.76
AAEL004223	Antibacterial peptide	17608.2	4622.73	−3.8	1.00	2.20
AAEL004851	Hypothetical protein	46960.7	9712.4	−4.835	1.00	1.77
AAEL000200	Hypothetical protein	464.005	75.3489	−6.158	1.00	0.62
AAEL001863	Zinc carboxypeptidase	1303.55	98.5612	−13.22	1.00	0.33
AAEL010776	Carboxypeptidase	34.4547	0.512953	−67.169	1.00	0.58

**Table 3:** The heat shock protein 20 (HSP20) gene has 19 homologs in the *A. aegypti* genome. RNA-seq data showed that the majority of the homologs were expressed differentially compared to those in uninfected mosquitoes. Nine of these homologs, which included the *l(2)efl* gene, were upregulated.

Gene ID	Gene Annotation	Abundance Level Compared to Uninfected Mosquitoes (2-log-fold)
AAEL003344	Metaxin	0
AAEL010654	*Lethal(2)-essential-for-life* protein, *l(2)efl*	−2.51386
AAEL010660	Alpha-B-crystallin, putative	−2.86544
AAEL010664	Actin binding protein, putative	−0.90751
AAEL010667	*Lethal(2)-essential-for-life* protein, *l(2)efl*	0
AAEL010670	*Lethal(2)-essential-for-life* protein, *l(2)efl*	2.80795
AAEL013338	*Lethal(2)-essential-for-life* protein, *l(2)efl*	3.3054
AAEL013339	AlphaA-crystallin, putative	0.816776
AAEL013340	*Lethal(2)-essential-for-life* protein, *l(2)efl*	−0.247543
AAEL013341	*Lethal(2)-essential-for-life* protein, *l(2)efl*	−1.30716
AAEL013344	*Lethal(2)-essential-for-life* protein, *l(2)efl*	3.03323
AAEL013345	AlphaA-crystallin, putative	0.752978
AAEL013346	*Lethal(2)-essential-for-life* protein, *l(2)efl*	1.63413
AAEL013347	*Lethal(2)-essential-for-life* protein, *l(2)efl*	−0.812587
AAEL013348	*Lethal(2)-essential-for-life* protein, *l(2)efl*	0
AAEL013349	*Lethal(2)-essential-for-life* protein, *l(2)efl*	1.00427
AAEL013350	Heat shock protein 26kD, putative	2.43882
AAEL013351	*Lethal(2)-essential-for-life* protein, *l(2)efl*	1.48215
AAEL013352	*Lethal(2)-essential-for-life* protein, *l(2)efl*	−0.581082

*L(2)efl* is expressed in DENV-2-infected or poly(I:C)-transfected CCL-125 cells and modulates.

## Supplementary Materials

The following are available online at https://www.mdpi.com/1422-0067/21/20/7520/s1, Figure S1: The Ct value among different experimental conditions on different time points for housekeeping genes 40s rps17 (A), actin (B), and rpL5 (C)., Table S1: List of primer sequences for dsRNA production, Table S2: List of primer sequences for RNA-seq validation, Table S3: List of primer sequences for *l(2)efl-2* to *l(2)efl-5*, Table S4: Upregulated genes at 14 days post dengue infection in *Aedes aegypt*i, Table S5: Downregulated genes at 14 days post dengue infection in *Aedes aegypti*.
