# Supplementary material for: The Lethal(2)-Essential-for-Life [L(2)EFL] Gene Family Modulates Dengue Virus Infection in Aedes aegypti
Source: Int J Mol Sci. 2020 Oct 12;21(20):7520. doi: 10.3390/ijms21207520 (PMC7593908; doi:10.3390/ijms21207520)
Supplement: Supplementary file 1 [file ijms-21-07520-s001.zip › Supplementary Data/Tables S4,5.docx]

Table S4: Upregulated genes at 14 days post dengue infection in *Aedes aegypti*

|  | | | | |
| --- | --- | --- | --- | --- |
|  |  |  |  |  |
| VectorBase ID | RPKM (blood fed) | RPKM (14 days post dengue infection) | log2 fold | VectorBase description |
|  |  |  |  |  |
| AAEL017748 | 0.78972 | 42.8413 | 5.76152 | U2 spliceosomal RNA |
| AAEL005098 | 1.01302 | 29.5448 | 4.86616 | cysteine-rich venom protein, putative [Source:VB External Description;Acc:AAEL005098] |
| AAEL003673 | 39.7839 | 910.855 | 4.51697 | histone H4 [Source:VB External Description;Acc:AAEL003673] |
| AAEL003659 | 39.3939 | 843.915 | 4.42105 | histone H3 [Source:VB External Description;Acc:AAEL003659] |
| AAEL015464 | 2.49608 | 46.4733 | 4.21867 | histone H1, putative [Source:VB External Description;Acc:AAEL015464] |
| AAEL015684 | 34.0523 | 612.329 | 4.16848 |  |
| AAEL009557 | 0.366199 | 6.23333 | 4.0893 | Niemann-Pick Type C-2, putative [Source:VB External Description;Acc:AAEL009557] |
| AAEL003685 | 31.7846 | 528.509 | 4.05553 | histone H3 [Source:VB External Description;Acc:AAEL003685] |
| AAEL017358 | 36.0798 | 595.473 | 4.04477 |  |
| AAEL003669 | 52.1774 | 767.836 | 3.8793 | histone H2A [Source:VB External Description;Acc:AAEL003669] |
| AAEL003689 | 69.5104 | 999.832 | 3.84639 | histone H4 [Source:VB External Description;Acc:AAEL003689] |
| AAEL015682 | 156.222 | 2238.43 | 3.84081 | histone H2B [Source:VB External Description;Acc:AAEL015682] |
| AAEL013338 | 743.143 | 7346.79 | 3.3054 | lethal(2)essential for life protein, l2efl [Source:VB External Description;Acc:AAEL013338] |
| AAEL012529 | 0.146114 | 1.41216 | 3.27273 |  |
| AAEL013286 | 30.0263 | 274.603 | 3.19305 | single-strand selective monofunctional uracil DNA glycosylase [Source:VB External Description;Acc:AAEL013286] |
| AAEL005212 | 77.2372 | 655.367 | 3.08494 |  |
| AAEL013344 | 0.966762 | 7.91431 | 3.03323 | lethal(2)essential for life protein, l2efl [Source:VB External Description;Acc:AAEL013344] |
| AAEL012339 | 0.813114 | 6.47846 | 2.99412 | cdk1 [Source:VB External Description;Acc:AAEL012339] |
| AAEL004537 | 59.823 | 469.633 | 2.97276 |  |
| AAEL003797 | 3.19108 | 24.7467 | 2.95512 |  |
| AAEL007097 | 918.192 | 6941.7 | 2.91842 | 4-nitrophenylphosphatase [Source:VB External Description;Acc:AAEL007097] |
| AAEL014524 | 0.307682 | 2.26126 | 2.87761 | DNA replication licensing factor MCM4 [Source:VB External Description;Acc:AAEL014524] |
| AAEL009263 | 33.3703 | 241.955 | 2.8581 |  |
| AAEL010855 | 8.18748 | 59.1695 | 2.85336 | cdc6 [Source:VB External Description;Acc:AAEL010855] |
| AAEL005501 | 10.6152 | 76.2026 | 2.84371 |  |
| AAEL010670 | 7.7611 | 54.3501 | 2.80795 | lethal(2)essential for life protein, l2efl [Source:VB External Description;Acc:AAEL010670] |
| AAEL009928 | 1.77256 | 12.3967 | 2.80605 | shoc2 [Source:VB External Description;Acc:AAEL009928] |
| AAEL008937 | 0.966353 | 6.72589 | 2.7991 |  |
| AAEL004937 | 169.925 | 1182.17 | 2.79847 |  |
| AAEL010644 | 38.5521 | 265.324 | 2.78287 | ribonucleoside-diphosphate reductase large chain [Source:VB External Description;Acc:AAEL010644] |
| AAEL013738 | 3.17817 | 20.3 | 2.67521 |  |
| AAEL012667 | 3.26487 | 20.7894 | 2.67075 |  |
| AAEL012668 | 3.26487 | 20.7894 | 2.67075 |  |
| AAEL009962 | 42.3215 | 265.25 | 2.64789 |  |
| AAEL006243 | 141.638 | 871.46 | 2.62123 |  |
| AAEL006239 | 141.638 | 871.46 | 2.62123 |  |
| AAEL004555 | 16.481 | 101.255 | 2.61912 |  |
| AAEL008519 | 23.2716 | 142.847 | 2.61782 | DNA replication factor Cdt1 [Source:VB External Description;Acc:AAEL008519] |
| AAEL003741 | 1.55905 | 9.46357 | 2.60171 |  |
| AAEL012499 | 61.8098 | 370.672 | 2.58423 | histone H2A [Source:VB External Description;Acc:AAEL012499] |
| AAEL010086 | 5.83329 | 34.2836 | 2.55514 | DNA replication licensing factor MCM4 [Source:VB External Description;Acc:AAEL010086] |
| AAEL005850 | 19.4315 | 114.135 | 2.55427 |  |
| AAEL005841 | 19.4315 | 114.135 | 2.55427 |  |
| AAEL004918 | 27.5103 | 157.91 | 2.52106 | brat protein [Source:VB External Description;Acc:AAEL004918] |
| AAEL010691 | 73.9351 | 422.41 | 2.51431 | ribonucleoside-diphosphate reductase small chain [Source:VB External Description;Acc:AAEL010691] |
| AAEL013329 | 1.55859 | 8.83841 | 2.50354 | cdk1 [Source:VB External Description;Acc:AAEL013329] |
| AAEL014542 | 75.1641 | 424.733 | 2.49844 |  |
| AAEL010155 | 118.883 | 670.828 | 2.4964 |  |
| AAEL018034 | 118.883 | 670.828 | 2.4964 |  |
| AAEL010717 | 20.1941 | 113.899 | 2.49575 | ecdysone receptor isoform-B (EcRB) [Source:VB External Description;Acc:AAEL010717] |
| AAEL018316 | 1.14887 | 6.42672 | 2.48386 |  |
| AAEL006241 | 2.62321 | 14.5063 | 2.46727 | sugar transporter [Source:VB External Description;Acc:AAEL006241] |
| AAEL011811 | 17.6271 | 96.7719 | 2.4568 | DNA replication licensing factor MCM3 [Source:VB External Description;Acc:AAEL011811] |
| AAEL007198 | 0.711939 | 3.89575 | 2.45208 | Osiris, putative [Source:VB External Description;Acc:AAEL007198] |
| AAEL001524 | 1.43712 | 7.86219 | 2.45175 | rab23 [Source:VB External Description;Acc:AAEL001524] |
| AAEL017098 | 551.876 | 2981.62 | 2.43368 |  |
| AAEL012546 | 17.598 | 94.5673 | 2.42593 | DNA replication licensing factor MCM6 [Source:VB External Description;Acc:AAEL012546] |
| AAEL003688 | 32.8051 | 175.375 | 2.41846 |  |
| AAEL010253 | 2.98549 | 15.9459 | 2.41714 |  |
| AAEL012188 | 1.238 | 6.53806 | 2.40085 |  |
| AAEL003804 | 1.26435 | 6.60053 | 2.38418 |  |
| AAEL010282 | 10.951 | 56.9834 | 2.37948 |  |
| AAEL002403 | 1.75679 | 9.13461 | 2.3784 |  |
| AAEL018162 | 9.62201 | 49.9254 | 2.37536 |  |
| AAEL002388 | 3.13474 | 16.1935 | 2.369 | zinc finger protein [Source:VB External Description;Acc:AAEL002388] |
| AAEL017329 | 9.83836 | 49.4519 | 2.32954 |  |
| AAEL017186 | 9.23442 | 46.2781 | 2.32524 |  |
| AAEL007007 | 20.4912 | 102.402 | 2.32116 | DNA replication licensing factor MCM2 [Source:VB External Description;Acc:AAEL007007] |
| AAEL013141 | 81.719 | 408.128 | 2.32028 |  |
| AAEL013146 | 81.719 | 408.128 | 2.32028 |  |
| AAEL012178 | 4.23139 | 20.9729 | 2.30933 |  |
| AAEL013145 | 133.266 | 650.525 | 2.2873 |  |
| AAEL000218 | 97.733 | 474.609 | 2.27982 |  |
| AAEL008182 | 89.7195 | 435.433 | 2.27896 |  |
| AAEL010185 | 1.77082 | 8.54992 | 2.2715 | A-kinase anchoring protein AKAP120, putative [Source:VB External Description;Acc:AAEL010185] |
| AAEL010097 | 454.562 | 2189.66 | 2.26816 |  |
| AAEL017931 | 77.3837 | 372.506 | 2.26716 | U1 spliceosomal RNA [Source:RFAM;Acc:RF00003] |
| AAEL010638 | 33.8707 | 162.746 | 2.26451 | histone H1, putative [Source:VB External Description;Acc:AAEL010638] |
| AAEL018236 | 0.435225 | 2.08771 | 2.26209 |  |
| AAEL001666 | 111.976 | 534.805 | 2.25582 |  |
| AAEL010081 | 9.47303 | 45.1412 | 2.25255 |  |
| AAEL011694 | 0.516375 | 2.45146 | 2.24715 |  |
| AAEL012224 | 4.71219 | 22.2867 | 2.24171 | zinc finger protein [Source:VB External Description;Acc:AAEL012224] |
| AAEL004130 | 9.57421 | 45.0509 | 2.23433 |  |
| AAEL002810 | 14.4106 | 67.0967 | 2.21912 | DNA replication licensing factor MCM5 [Source:VB External Description;Acc:AAEL002810] |
| AAEL018047 | 4.30307 | 20.0096 | 2.21725 |  |
| AAEL003795 | 8.88067 | 41.112 | 2.21082 |  |
| AAEL000601 | 3.39734 | 15.6546 | 2.20411 |  |
| AAEL017387 | 3.7069 | 17.0232 | 2.19922 |  |
| AAEL006389 | 35.1925 | 161.488 | 2.19809 | cathepsin l [Source:VB External Description;Acc:AAEL006389] |
| AAEL012203 | 0.785259 | 3.60269 | 2.19783 |  |
| AAEL013686 | 4.91642 | 22.5512 | 2.19752 |  |
| AAEL006660 | 8.46455 | 38.7698 | 2.19543 |  |
| AAEL006179 | 2.14809 | 9.72854 | 2.17917 | tubulin alpha chain [Source:VB External Description;Acc:AAEL006179] |
| AAEL008301 | 3.91195 | 17.6813 | 2.17626 | eph receptor tyrosine kinase [Source:VB External Description;Acc:AAEL008301] |
| AAEL011250 | 11.7934 | 53.1805 | 2.17292 |  |
| AAEL000425 | 2.42968 | 10.8736 | 2.16199 |  |
| AAEL008316 | 13.7978 | 61.5292 | 2.15683 | mitotic spindle assembly checkpoint protein mad2 [Source:VB External Description;Acc:AAEL008316] |
| AAEL011396 | 18.4999 | 81.149 | 2.13305 | neurogenic locus delta protein [Source:VB External Description;Acc:AAEL011396] |
| AAEL007438 | 5.56679 | 24.3545 | 2.12927 | dipeptidyl-peptidase [Source:VB External Description;Acc:AAEL007438] |
| AAEL011430 | 12.0153 | 52.5339 | 2.12838 |  |
| AAEL007046 | 24.433 | 106.777 | 2.1277 | mitochondrial brown fat uncoupling protein [Source:VB External Description;Acc:AAEL007046] |
| AAEL011235 | 7.43119 | 32.2071 | 2.11572 |  |
| AAEL003981 | 49.4495 | 214.039 | 2.11385 |  |
| AAEL002778 | 36.7265 | 157.031 | 2.09615 |  |
| AAEL014718 | 63.1564 | 267.675 | 2.08348 |  |
| AAEL014716 | 63.1564 | 267.675 | 2.08348 |  |
| AAEL000006 | 43.6727 | 184.168 | 2.07622 |  |
| AAEL000025 | 43.6727 | 184.168 | 2.07622 |  |
| AAEL007479 | 51.1811 | 215.772 | 2.07583 |  |
| AAEL002477 | 13.3013 | 55.9946 | 2.07372 |  |
| AAEL006037 | 7.36271 | 30.6052 | 2.05547 |  |
| AAEL000584 | 27.4654 | 114.022 | 2.05362 | sex-determining region y protein, sry [Source:VB External Description;Acc:AAEL000584] |
| AAEL002820 | 4.72472 | 19.4535 | 2.04173 |  |
| AAEL000604 | 87.2 | 356.322 | 2.03078 |  |
| AAEL001246 | 33.7813 | 137.938 | 2.02973 | Thymidylate kinase, putative [Source:VB External Description;Acc:AAEL001246] |
| AAEL000776 | 162.022 | 659.586 | 2.02537 |  |
| AAEL014025 | 27.9094 | 112.613 | 2.01255 | cell division cycle 20 (cdc20) (fizzy) [Source:VB External Description;Acc:AAEL014025] |
| AAEL006782 | 21.0254 | 84.5764 | 2.00812 | histone-arginine methyltransferase CARMER [Source:VB Community Annotation;Acc:AAEL006782] |
| AAEL008621 | 24.2416 | 97.5011 | 2.00793 | cdk1 [Source:VB External Description;Acc:AAEL008621] |
| AAEL012362 | 2.9704 | 11.9401 | 2.00708 | WD-repeat protein [Source:VB External Description;Acc:AAEL012362] |
| AAEL001135 | 6.23519 | 24.9788 | 2.0022 |  |

Table S5: Downregulated genes at 14 days post dengue infection in *Aedes aegypti*

|  | | | | | | | | | | |  |  |  |  |  |  |
| --- | --- | --- | --- | --- | --- | --- | --- | --- | --- | --- | --- | --- | --- | --- | --- | --- |
|  |  |  |  | |  |  |  |  |  |  |  |  |  |  |  |  |
| VectorBase ID | RPKM (blood fed) | RPKM (14 days post dengue infection) | log2 fold | VectorBase description | | |  |  |  |  |  |  |  |  |  |  |
|  |  |  |  |  | |  |  |  |  |  |  |  |  |  |  |  |
| AAEL010776 | 34.4547 | 0.512953 | -6.06973 | carboxypeptidase [Source:VB External Description;Acc:AAEL010776] | | | | | | |  |  |  |  |  |  |
| AAEL012858 | 62.9787 | 1.01427 | -5.95635 |  | |  |  |  |  |  |  |  |  |  |  |  |
| AAEL013555 | 23.9259 | 0.487024 | -5.61843 | cytochrome P450 (CYP4J13) [Source:VB Community Annotation;Acc:AAEL013555] | | | | | | | |  |  |  |  |  |
| AAEL009899 | 131.338 | 3.03213 | -5.4368 |  | |  |  |  |  |  |  |  |  |  |  |  |
| AAEL001417 | 16.3778 | 0.381873 | -5.4225 | leucine-rich immune protein (Short) [Source:VB Community Annotation;Acc:AAEL001417] | | | | | | | | |  |  |  |  |
| AAEL009669 | 9.15652 | 0.21552 | -5.4089 |  | |  |  |  |  |  |  |  |  |  |  |  |
| AAEL004701 | 7.66932 | 0.209825 | -5.19184 | Argininosuccinate synthase (EC 6.3.4.5)(Citrulline--aspartate ligase) [Source:VB External Description;Acc:AAEL004701] | | | | | | | | | | |  |  |
| AAEL001690 | 13.9412 | 0.409666 | -5.08877 | serine-type enodpeptidase, [Source:VB External Description;Acc:AAEL001690] | | | | | | | |  |  |  |  |  |
| AAEL001679 | 65.1524 | 2.06228 | -4.98151 |  | |  |  |  |  |  |  |  |  |  |  |  |
| AAEL005416 | 56.859 | 1.81244 | -4.97138 | oxidase/peroxidase [Source:VB External Description;Acc:AAEL005416] | | | | | | |  |  |  |  |  |  |
| AAEL002378 | 12.3272 | 0.396542 | -4.95823 |  | |  |  |  |  |  |  |  |  |  |  |  |
| AAEL012855 | 28.8204 | 0.944123 | -4.93197 |  | |  |  |  |  |  |  |  |  |  |  |  |
| AAEL010634 | 52.6025 | 1.95128 | -4.75264 |  | |  |  |  |  |  |  |  |  |  |  |  |
| AAEL001402 | 129.498 | 4.92187 | -4.71758 | leucine-rich immune protein (Short) [Source:VB Community Annotation;Acc:AAEL001402] | | | | | | | | |  |  |  |  |
| AAEL009513 | 12.6089 | 0.479588 | -4.71651 | adenylate cyclase [Source:VB External Description;Acc:AAEL009513] | | | | | | |  |  |  |  |  |  |
| AAEL000650 | 15.7805 | 0.608572 | -4.69657 |  | |  |  |  |  |  |  |  |  |  |  |  |
| AAEL001414 | 106.486 | 4.45325 | -4.57966 | leucine-rich immune protein (Short) [Source:VB Community Annotation;Acc:AAEL001414] | | | | | | | | |  |  |  |  |
| AAEL001420 | 322.944 | 13.5129 | -4.57887 | leucine-rich immune protein (Short) [Source:VB Community Annotation;Acc:AAEL001420] | | | | | | | | |  |  |  |  |
| AAEL018185 | 6.61967 | 0.284636 | -4.53957 |  | |  |  |  |  |  |  |  |  |  |  |  |
| AAEL002576 | 17.9226 | 0.779719 | -4.52268 | sodium/solute symporter [Source:VB External Description;Acc:AAEL002576] | | | | | | | |  |  |  |  |  |
| AAEL013857 | 27.8397 | 1.22805 | -4.5027 |  | |  |  |  |  |  |  |  |  |  |  |  |
| AAEL010396 | 4.16565 | 0.187708 | -4.47198 | secreted ferritin G subunit precursor, putative [Source:VB External Description;Acc:AAEL010396] | | | | | | | | |  |  |  |  |
| AAEL001401 | 71.6693 | 3.31716 | -4.43333 | leucine-rich immune protein (Short) [Source:VB Community Annotation;Acc:AAEL001401] | | | | | | | | |  |  |  |  |
| AAEL010276 | 44.6689 | 2.25014 | -4.31118 | aminomethyltransferase [Source:VB External Description;Acc:AAEL010276] | | | | | | | |  |  |  |  |  |
| AAEL004547 | 114.593 | 5.82344 | -4.2985 |  | |  |  |  |  |  |  |  |  |  |  |  |
| AAEL000196 | 4.84825 | 0.250082 | -4.27699 |  | |  |  |  |  |  |  |  |  |  |  |  |
| AAEL013542 | 13.0117 | 0.673515 | -4.27196 | elongase, putative [Source:VB External Description;Acc:AAEL013542] | | | | | | |  |  |  |  |  |  |
| AAEL002686 | 6.53355 | 0.338271 | -4.27161 | testisin precursor, putative [Source:VB External Description;Acc:AAEL002686] | | | | | | | |  |  |  |  |  |
| AAEL004798 | 38.7418 | 2.01996 | -4.26149 |  | |  |  |  |  |  |  |  |  |  |  |  |
| AAEL003483 | 107.785 | 5.69765 | -4.24165 |  | |  |  |  |  |  |  |  |  |  |  |  |
| AAEL014004 | 5.4659 | 0.304217 | -4.16729 | clip-domain serine protease, putative [Source:VB External Description;Acc:AAEL014004] | | | | | | | | |  |  |  |  |
| AAEL003235 | 88.3893 | 4.97372 | -4.15148 |  | |  |  |  |  |  |  |  |  |  |  |  |
| AAEL007653 | 75.3216 | 4.24495 | -4.14925 | allantoinase [Source:VB External Description;Acc:AAEL007653] | | | | | | |  |  |  |  |  |  |
| AAEL011510 | 8.18648 | 0.462474 | -4.1458 | multiple inositol polyphosphate phosphatase [Source:VB External Description;Acc:AAEL011510] | | | | | | | | |  |  |  |  |
| AAEL012856 | 49.3277 | 2.82191 | -4.12766 |  | |  |  |  |  |  |  |  |  |  |  |  |
| AAEL010028 | 5.82935 | 0.334058 | -4.12517 | sarcosine dehydrogenase [Source:VB External Description;Acc:AAEL010028] | | | | | | | |  |  |  |  |  |
| AAEL006576 | 93.2441 | 5.3607 | -4.12052 | clip-domain serine protease, putative [Source:VB External Description;Acc:AAEL006576] | | | | | | | | |  |  |  |  |
| AAEL000500 | 28.8949 | 1.70279 | -4.08484 |  | |  |  |  |  |  |  |  |  |  |  |  |
| AAEL017536 | 11965.5 | 711.375 | -4.07213 | holotricin glycine rich repeat protein (GRRP) anti-microbial peptide [Source:VB Community Annotation;Acc:AAEL017536] | | | | | | | | | | |  |  |
| AAEL000311 | 35.2824 | 2.10029 | -4.07029 |  | |  |  |  |  |  |  |  |  |  |  |  |
| AAEL012767 | 13.3303 | 0.801447 | -4.05595 | leucine-rich immune protein (Short) [Source:VB Community Annotation;Acc:AAEL012767] | | | | | | | | |  |  |  |  |
| AAEL013484 | 1012.4 | 61.3704 | -4.04409 |  | |  |  |  |  |  |  |  |  |  |  |  |
| AAEL013486 | 1012.4 | 61.3704 | -4.04409 |  | |  |  |  |  |  |  |  |  |  |  |  |
| AAEL001792 | 7.16591 | 0.442143 | -4.01856 | sodium/solute symporter [Source:VB External Description;Acc:AAEL001792] | | | | | | | |  |  |  |  |  |
| AAEL009359 | 8.83259 | 0.548172 | -4.01013 |  | |  |  |  |  |  |  |  |  |  |  |  |
| AAEL014855 | 3.10297 | 0.193274 | -4.00493 |  | |  |  |  |  |  |  |  |  |  |  |  |
| AAEL000101 | 5.1636 | 0.329578 | -3.96968 | AMP dependent coa ligase [Source:VB External Description;Acc:AAEL000101] | | | | | | | |  |  |  |  |  |
| AAEL010884 | 63.4735 | 4.08534 | -3.95763 | ADP,ATP carrier protein [Source:VB External Description;Acc:AAEL010884] | | | | | | | |  |  |  |  |  |
| AAEL008876 | 5.08487 | 0.334917 | -3.92433 | deoxyribonuclease I, putative [Source:VB External Description;Acc:AAEL008876] | | | | | | | |  |  |  |  |  |
| AAEL006161 | 10.8296 | 0.715144 | -3.9206 | Clip-Domain Serine Protease family B [Source:VB Community Annotation;Acc:AAEL006161] | | | | | | | | |  |  |  |  |
| AAEL006662 | 43.2942 | 2.89923 | -3.90043 |  | |  |  |  |  |  |  |  |  |  |  |  |
| AAEL000059 | 19.7519 | 1.33514 | -3.88693 | Clip-Domain Serine Protease family B. Protease homologue. [Source:VB Community Annotation;Acc:AAEL000059] | | | | | | | | | | |  |  |
| AAEL004014 | 3.51831 | 0.239393 | -3.87743 | glucose dehydrogenase [Source:VB External Description;Acc:AAEL004014] | | | | | | | |  |  |  |  |  |
| AAEL012255 | 5.95792 | 0.412595 | -3.85201 | leucine-rich immune protein (Short) [Source:VB Community Annotation;Acc:AAEL012255] | | | | | | | | |  |  |  |  |
| AAEL007090 | 14.2352 | 0.989542 | -3.84655 | 4-nitrophenylphosphatase [Source:VB External Description;Acc:AAEL007090] | | | | | | | |  |  |  |  |  |
| AAEL002731 | 4.26524 | 0.299639 | -3.83133 | Serine Protease Inhibitor (serpin) homologue - unlikely to be inhibitory. [Source:VB Community Annotation;Acc:AAEL002731] | | | | | | | | | | | |  |
| AAEL011448 | 11.8489 | 0.833121 | -3.83008 |  | |  |  |  |  |  |  |  |  |  |  |  |
| AAEL007103 | 27.6796 | 1.97037 | -3.81228 | leucine-rich immune protein (TM) [Source:VB Community Annotation;Acc:AAEL007103] | | | | | | | | |  |  |  |  |
| AAEL008753 | 10.6907 | 0.766034 | -3.80281 |  | |  |  |  |  |  |  |  |  |  |  |  |
| AAEL010125 | 42.7211 | 3.06231 | -3.80225 | leucine-rich immune protein (Coil-less) [Source:VB Community Annotation;Acc:AAEL010125] | | | | | | | | |  |  |  |  |
| AAEL000642 | 6.25128 | 0.455507 | -3.77861 | alpha-amylase [Source:VB External Description;Acc:AAEL000642] | | | | | | |  |  |  |  |  |  |
| AAEL002696 | 4.45028 | 0.327657 | -3.76364 |  | |  |  |  |  |  |  |  |  |  |  |  |
| AAEL007033 | 20.3356 | 1.51042 | -3.75098 | pyrroline-5-carboxylate reductase [Source:VB External Description;Acc:AAEL007033] | | | | | | | | |  |  |  |  |
| AAEL003405 | 41.6666 | 3.14732 | -3.7267 |  | |  |  |  |  |  |  |  |  |  |  |  |
| AAEL001863 | 1303.55 | 98.5612 | -3.72528 | zinc carboxypeptidase [Source:VB External Description;Acc:AAEL001863] | | | | | | | |  |  |  |  |  |
| AAEL012136 | 9.39327 | 0.713277 | -3.71909 | allergen, putative [Source:VB External Description;Acc:AAEL012136] | | | | | | |  |  |  |  |  |  |
| AAEL007878 | 1.77189 | 0.136314 | -3.70028 | ornithine decarboxylase [Source:VB External Description;Acc:AAEL007878] | | | | | | | |  |  |  |  |  |
| AAEL005178 | 10.5028 | 0.812352 | -3.69252 |  | |  |  |  |  |  |  |  |  |  |  |  |
| AAEL011130 | 22.8514 | 1.77379 | -3.68737 | alcohol dehydrogenase [Source:VB External Description;Acc:AAEL011130] | | | | | | | |  |  |  |  |  |
| AAEL008494 | 85.4003 | 6.66108 | -3.68041 | mitochondrial carrier protein ymc [Source:VB External Description;Acc:AAEL008494] | | | | | | | |  |  |  |  |  |
| AAEL001295 | 9.00198 | 0.703177 | -3.67828 |  | |  |  |  |  |  |  |  |  |  |  |  |
| AAEL013433 | 9.00209 | 0.703985 | -3.67664 | spaetzle-like cytokine [Source:VB Community Annotation;Acc:AAEL013433] | | | | | | | |  |  |  |  |  |
| AAEL003000 | 41.7017 | 3.27797 | -3.66923 |  | |  |  |  |  |  |  |  |  |  |  |  |
| AAEL015450 | 29.7067 | 2.33819 | -3.66733 | ribonuclease UK114, putative [Source:VB External Description;Acc:AAEL015450] | | | | | | | |  |  |  |  |  |
| AAEL008473 | 147.658 | 11.6677 | -3.66167 | cysteine-rich venom protein, putative [Source:VB External Description;Acc:AAEL008473] | | | | | | | | |  |  |  |  |
| AAEL011453 | 14.4542 | 1.14319 | -3.66036 | C-Type Lectin (CTL14) [Source:VB Community Annotation;Acc:AAEL011453] | | | | | | | |  |  |  |  |  |
| AAEL006319 | 3.73793 | 0.296235 | -3.65743 |  | |  |  |  |  |  |  |  |  |  |  |  |
| AAEL011008 | 6.20385 | 0.503016 | -3.62449 | lipase [Source:VB External Description;Acc:AAEL011008] | | | | | |  |  |  |  |  |  |  |
| AAEL014516 | 8.04153 | 0.652781 | -3.6228 | metalloproteinase, putative [Source:VB External Description;Acc:AAEL014516] | | | | | | | |  |  |  |  |  |
| AAEL008844 | 153.878 | 12.5617 | -3.61469 |  | |  |  |  |  |  |  |  |  |  |  |  |
| AAEL016994 | 153.878 | 12.5617 | -3.61469 |  | |  |  |  |  |  |  |  |  |  |  |  |
| AAEL014937 | 1137 | 92.9078 | -3.61329 |  | |  |  |  |  |  |  |  |  |  |  |  |
| AAEL011542 | 94.6652 | 7.8337 | -3.59507 | metalloproteinase, putative [Source:VB External Description;Acc:AAEL011542] | | | | | | | |  |  |  |  |  |
| AAEL001757 | 4.57385 | 0.378693 | -3.59431 | iduronate 2-sulfatase precursor [Source:VB External Description;Acc:AAEL001757] | | | | | | | |  |  |  |  |  |
| AAEL017380 | 100.894 | 8.37844 | -3.59002 |  | |  |  |  |  |  |  |  |  |  |  |  |
| AAEL005482 | 49.4114 | 4.10698 | -3.5887 | C-Type Lectin (CTL). [Source:VB Community Annotation;Acc:AAEL005482] | | | | | | | |  |  |  |  |  |
| AAEL006004 | 17.063 | 1.42508 | -3.58175 |  | |  |  |  |  |  |  |  |  |  |  |  |
| AAEL000749 | 56.8575 | 4.78446 | -3.57092 |  | |  |  |  |  |  |  |  |  |  |  |  |
| AAEL002858 | 2.18882 | 0.187947 | -3.54175 |  | |  |  |  |  |  |  |  |  |  |  |  |
| AAEL008620 | 12.2726 | 1.06012 | -3.53314 | D7 protein, putative [Source:VB External Description;Acc:AAEL008620] | | | | | | |  |  |  |  |  |  |
| AAEL009904 | 7.06668 | 0.610459 | -3.53307 |  | |  |  |  |  |  |  |  |  |  |  |  |
| AAEL006248 | 13.7496 | 1.1886 | -3.53205 | p37NB protein, putative [Source:VB External Description;Acc:AAEL006248] | | | | | | | |  |  |  |  |  |
| AAEL005480 | 44.6819 | 3.87971 | -3.52567 | hairy protein [Source:VB External Description;Acc:AAEL005480] | | | | | | |  |  |  |  |  |  |
| AAEL012341 | 61.9388 | 5.37863 | -3.52553 | lysosomal acid lipase, putative [Source:VB External Description;Acc:AAEL012341] | | | | | | | |  |  |  |  |  |
| AAEL006568 | 53.6539 | 4.67567 | -3.52044 | serine protease [Source:VB External Description;Acc:AAEL006568] | | | | | | |  |  |  |  |  |  |
| AAEL009925 | 10.2183 | 0.894238 | -3.51436 | amidase [Source:VB External Description;Acc:AAEL009925] | | | | | |  |  |  |  |  |  |  |
| AAEL000546 | 20.5352 | 1.80798 | -3.50565 |  | |  |  |  |  |  |  |  |  |  |  |  |
| AAEL006834 | 108.073 | 9.5889 | -3.49449 | glutamate semialdehyde dehydrogenase [Source:VB External Description;Acc:AAEL006834] | | | | | | | | |  |  |  |  |
| AAEL007128 | 17.9958 | 1.59798 | -3.49334 | sugar transporter [Source:VB External Description;Acc:AAEL007128] | | | | | | |  |  |  |  |  |  |
| AAEL002969 | 24.5074 | 2.1809 | -3.49022 | brain chitinase and chia [Source:VB External Description;Acc:AAEL002969] | | | | | | | |  |  |  |  |  |
| AAEL001816 | 15.9892 | 1.43183 | -3.48116 | glucosyl/glucuronosyl transferases [Source:VB External Description;Acc:AAEL001816] | | | | | | | | |  |  |  |  |
| AAEL014936 | 1.82002 | 0.164581 | -3.46709 | sarcosine dehydrogenase [Source:VB External Description;Acc:AAEL014936] | | | | | | | |  |  |  |  |  |
| AAEL004120 | 39.9105 | 3.62148 | -3.46212 | Niemann-Pick Type C-2, putative [Source:VB External Description;Acc:AAEL004120] | | | | | | | |  |  |  |  |  |
| AAEL005961 | 57.2219 | 5.20848 | -3.45763 | actin [Source:VB External Description;Acc:AAEL005961] | | | | | |  |  |  |  |  |  |  |
| AAEL009625 | 19.2228 | 1.75451 | -3.45367 | short-chain dehydrogenase [Source:VB External Description;Acc:AAEL009625] | | | | | | | |  |  |  |  |  |
| AAEL013655 | 42.6164 | 3.89029 | -3.45346 |  | |  |  |  |  |  |  |  |  |  |  |  |
| AAEL002655 | 7.33341 | 0.669481 | -3.45337 | matrix metalloproteinase [Source:VB External Description;Acc:AAEL002655] | | | | | | | |  |  |  |  |  |
| AAEL001293 | 12.2195 | 1.12418 | -3.44225 |  | |  |  |  |  |  |  |  |  |  |  |  |
| AAEL008285 | 10.1121 | 0.939892 | -3.42744 | pupal cuticle protein, putative [Source:VB External Description;Acc:AAEL008285] | | | | | | | |  |  |  |  |  |
| AAEL010782 | 46.8437 | 4.35734 | -3.42634 | carboxypeptidase [Source:VB External Description;Acc:AAEL010782] | | | | | | |  |  |  |  |  |  |
| AAEL006971 | 51.392 | 4.78475 | -3.42503 |  | |  |  |  |  |  |  |  |  |  |  |  |
| AAEL006377 | 26.6696 | 2.49617 | -3.41741 | leucine-rich immune protein (Coil-less) [Source:VB Community Annotation;Acc:AAEL006377] | | | | | | | | |  |  |  |  |
| AAEL001897 | 5.09179 | 0.477728 | -3.41391 |  | |  |  |  |  |  |  |  |  |  |  |  |
| AAEL005001 | 45.3632 | 4.26543 | -3.41076 | aquaporin [Source:VB External Description;Acc:AAEL005001] | | | | | | |  |  |  |  |  |  |
| AAEL011400 | 17.8411 | 1.68974 | -3.40033 |  | |  |  |  |  |  |  |  |  |  |  |  |
| AAEL005741 | 95.7332 | 9.09362 | -3.39609 |  | |  |  |  |  |  |  |  |  |  |  |  |
| AAEL005731 | 95.7332 | 9.09362 | -3.39609 |  | |  |  |  |  |  |  |  |  |  |  |  |
| AAEL005748 | 23.8903 | 2.27221 | -3.39425 | elastase, putative [Source:VB External Description;Acc:AAEL005748] | | | | | | |  |  |  |  |  |  |
| AAEL017875 | 21.8251 | 2.08823 | -3.38564 | microRNA mir-932 [Source:RFAM;Acc:RF01914] | | | | |  |  |  |  |  |  |  |  |
| AAEL004407 | 24.6734 | 2.36311 | -3.3842 | allergen, putative [Source:VB External Description;Acc:AAEL004407] | | | | | | |  |  |  |  |  |  |
| AAEL011574 | 16.4825 | 1.58313 | -3.38008 |  | |  |  |  |  |  |  |  |  |  |  |  |
| AAEL001305 | 11.7059 | 1.12539 | -3.37874 |  | |  |  |  |  |  |  |  |  |  |  |  |
| AAEL010636 | 2.42214 | 0.233236 | -3.37642 |  | |  |  |  |  |  |  |  |  |  |  |  |
| AAEL014361 | 5.21421 | 0.506389 | -3.36413 | amidase [Source:VB External Description;Acc:AAEL014361] | | | | | |  |  |  |  |  |  |  |
| AAEL007139 | 5.91904 | 0.577182 | -3.35827 | sugar transporter [Source:VB External Description;Acc:AAEL007139] | | | | | | |  |  |  |  |  |  |
| AAEL001737 | 7.10167 | 0.692684 | -3.35789 |  | |  |  |  |  |  |  |  |  |  |  |  |
| AAEL006096 | 52.0779 | 5.08719 | -3.35573 | Gelsolin precursor [Source:VB External Description;Acc:AAEL006096] | | | | | | |  |  |  |  |  |  |
| AAEL003259 | 13.6395 | 1.3336 | -3.35439 | pupal cuticle protein 78E, putative [Source:VB External Description;Acc:AAEL003259] | | | | | | | | |  |  |  |  |
| AAEL009761 | 3.04258 | 0.299047 | -3.34685 |  | |  |  |  |  |  |  |  |  |  |  |  |
| AAEL003060 | 4053.03 | 400.461 | -3.33927 | serine-type enodpeptidase, [Source:VB External Description;Acc:AAEL003060] | | | | | | | |  |  |  |  |  |
| AAEL010132 | 9.31175 | 0.922253 | -3.33582 | leucine-rich immune protein (Long) [Source:VB Community Annotation;Acc:AAEL010132] | | | | | | | | |  |  |  |  |
| AAEL008345 | 18.2573 | 1.81694 | -3.32889 | cytochrome P450 (CYP4G35) [Source:VB Community Annotation;Acc:AAEL008345] | | | | | | | |  |  |  |  |  |
| AAEL007814 | 3.17868 | 0.317366 | -3.3242 | n-twist [Source:VB External Description;Acc:AAEL007814] | | | | | |  |  |  |  |  |  |  |
| AAEL008307 | 6.13583 | 0.615041 | -3.3185 |  | |  |  |  |  |  |  |  |  |  |  |  |
| AAEL012089 | 33.1031 | 3.32153 | -3.31705 |  | |  |  |  |  |  |  |  |  |  |  |  |
| AAEL010075 | 7.98455 | 0.80357 | -3.31272 | oxidoreductase [Source:VB External Description;Acc:AAEL010075] | | | | | | |  |  |  |  |  |  |
| AAEL003457 | 17.8952 | 1.80471 | -3.30973 |  | |  |  |  |  |  |  |  |  |  |  |  |
| AAEL006572 | 319.852 | 32.2831 | -3.30855 | troponin C [Source:VB External Description;Acc:AAEL006572] | | | | | | |  |  |  |  |  |  |
| AAEL002610 | 95.5224 | 9.64235 | -3.30838 | serine protease [Source:VB External Description;Acc:AAEL002610] | | | | | | |  |  |  |  |  |  |
| AAEL002730 | 7.84932 | 0.793713 | -3.30588 | Serine Protease Inhibitor (serpin) likely cleavage at R/V. [Source:VB Community Annotation;Acc:AAEL002730] | | | | | | | | | | |  |  |
| AAEL005316 | 2.21403 | 0.225061 | -3.29829 |  | |  |  |  |  |  |  |  |  |  |  |  |
| AAEL001594 | 7.41707 | 0.754228 | -3.29778 |  | |  |  |  |  |  |  |  |  |  |  |  |
| AAEL006102 | 65.2456 | 6.71223 | -3.28102 | gelsolin precursor [Source:VB External Description;Acc:AAEL006102] | | | | | | |  |  |  |  |  |  |
| AAEL014019 | 8.36076 | 0.861166 | -3.27927 | cytochrome P450 (CYP4J16) [Source:VB Community Annotation;Acc:AAEL014019] | | | | | | | |  |  |  |  |  |
| AAEL004213 | 2.57576 | 0.266794 | -3.2712 | monocarboxylate transporter [Source:VB External Description;Acc:AAEL004213] | | | | | | | |  |  |  |  |  |
| AAEL009915 | 1.74741 | 0.181335 | -3.26849 | centrosomal protein [Source:VB External Description;Acc:AAEL009915] | | | | | | |  |  |  |  |  |  |
| AAEL018678 | 225.829 | 23.4572 | -3.26713 | NADH dehydrogenase subunit 5 [Source:VB Community Annotation;Acc:AAEL018678] | | | | | | | |  |  |  |  |  |
| AAEL005004 | 9.0612 | 0.941517 | -3.26664 |  | |  |  |  |  |  |  |  |  |  |  |  |
| AAEL014553 | 3.6013 | 0.375042 | -3.26339 | triacylglycerol lipase, pancreatic [Source:VB External Description;Acc:AAEL014553] | | | | | | | |  |  |  |  |  |
| AAEL008163 | 1.98287 | 0.20669 | -3.26205 | protease m1 zinc metalloprotease [Source:VB External Description;Acc:AAEL008163] | | | | | | | |  |  |  |  |  |
| AAEL013364 | 28.3261 | 2.96354 | -3.25674 |  | |  |  |  |  |  |  |  |  |  |  |  |
| AAEL002624 | 23.6884 | 2.49245 | -3.24854 | serine protease [Source:VB External Description;Acc:AAEL002624] | | | | | | |  |  |  |  |  |  |
| AAEL005789 | 12.9431 | 1.36877 | -3.24123 |  | |  |  |  |  |  |  |  |  |  |  |  |
| AAEL006674 | 76.733 | 8.12855 | -3.23878 | Clip-Domain Serine Protease family B. [Source:VB Community Annotation;Acc:AAEL006674] | | | | | | | | |  |  |  |  |
| AAEL000786 | 59.4084 | 6.29393 | -3.23863 |  | |  |  |  |  |  |  |  |  |  |  |  |
| AAEL009955 | 88.6965 | 9.41097 | -3.23646 |  | |  |  |  |  |  |  |  |  |  |  |  |
| AAEL008701 | 209.891 | 22.3159 | -3.2335 | myoinositol oxygenase [Source:VB External Description;Acc:AAEL008701] | | | | | | | |  |  |  |  |  |
| AAEL001766 | 2.07265 | 0.220463 | -3.23286 | leucine-rich transmembrane proteins [Source:VB External Description;Acc:AAEL001766] | | | | | | | | |  |  |  |  |
| AAEL002601 | 22.3946 | 2.40342 | -3.21999 | Clip-Domain Serine Protease family A. Protease homologue. [Source:VB Community Annotation;Acc:AAEL002601] | | | | | | | | | | |  |  |
| AAEL004814 | 13.7502 | 1.47913 | -3.21664 | potassium-dependent sodium-calcium exchanger, putative [Source:VB External Description;Acc:AAEL004814] | | | | | | | | | |  |  |  |
| AAEL001328 | 118.948 | 12.8499 | -3.2105 |  | |  |  |  |  |  |  |  |  |  |  |  |
| AAEL001343 | 118.948 | 12.8499 | -3.2105 |  | |  |  |  |  |  |  |  |  |  |  |  |
| AAEL000760 | 7.37004 | 0.80027 | -3.20311 | Clip-Domain Serine Protease family B. [Source:VB Community Annotation;Acc:AAEL000760] | | | | | | | | |  |  |  |  |
| AAEL013707 | 10.2111 | 1.10924 | -3.20249 | trypsin [Source:VB External Description;Acc:AAEL013707] | | | | | |  |  |  |  |  |  |  |
| AAEL000774 | 26.0078 | 2.82853 | -3.20082 |  | |  |  |  |  |  |  |  |  |  |  |  |
| AAEL003482 | 34.8479 | 3.79369 | -3.1994 |  | |  |  |  |  |  |  |  |  |  |  |  |
| AAEL014798 | 2.58826 | 0.282378 | -3.19628 | lung carbonyl reductase [Source:VB External Description;Acc:AAEL014798] | | | | | | | |  |  |  |  |  |
| AAEL006537 | 15.1454 | 1.65251 | -3.19615 |  | |  |  |  |  |  |  |  |  |  |  |  |
| AAEL010921 | 2.85332 | 0.311386 | -3.19586 | organic anion transporter [Source:VB External Description;Acc:AAEL010921] | | | | | | | |  |  |  |  |  |
| AAEL002535 | 3.17655 | 0.35016 | -3.18138 | nucleoprotein, putative [Source:VB External Description;Acc:AAEL002535] | | | | | | | |  |  |  |  |  |
| AAEL007796 | 8.01216 | 0.88596 | -3.17688 | Clip-Domain Serine Protease family D [Source:VB Community Annotation;Acc:AAEL007796] | | | | | | | | |  |  |  |  |
| AAEL006607 | 43.8111 | 4.88589 | -3.1646 | juvenile hormone-inducible protein, putative [Source:VB External Description;Acc:AAEL006607] | | | | | | | | |  |  |  |  |
| AAEL001022 | 72.327 | 8.07244 | -3.16346 | anterior fat body protein [Source:VB External Description;Acc:AAEL001022] | | | | | | | |  |  |  |  |  |
| AAEL018641 | 61.1116 | 6.84382 | -3.15857 | microRNA MIR82 [Source:VB Community Annotation;Acc:AAEL018641] | | | | | | |  |  |  |  |  |  |
| AAEL015304 | 2.58705 | 0.290251 | -3.15593 |  | |  |  |  |  |  |  |  |  |  |  |  |
| AAEL006390 | 38.5731 | 4.34009 | -3.1518 | vacuolar proton ATPases [Source:VB External Description;Acc:AAEL006390] | | | | | | | |  |  |  |  |  |
| AAEL017573 | 27.486 | 3.09335 | -3.15146 |  | |  |  |  |  |  |  |  |  |  |  |  |
| AAEL005883 | 10.7184 | 1.20645 | -3.15125 |  | |  |  |  |  |  |  |  |  |  |  |  |
| AAEL014950 | 5.17826 | 0.583115 | -3.15061 | spaetzle-like cytokine [Source:VB Community Annotation;Acc:AAEL014950] | | | | | | | |  |  |  |  |  |
| AAEL014893 | 83.4698 | 9.41708 | -3.1479 | cytochrome P450 [Source:VB Community Annotation;Acc:AAEL014893] | | | | | | |  |  |  |  |  |  |
| AAEL003842 | 17.3655 | 1.96077 | -3.14674 |  | |  |  |  |  |  |  |  |  |  |  |  |
| AAEL005386 | 0.864978 | 0.0977431 | -3.1456 | collagen alpha chain, anopheles [Source:VB External Description;Acc:AAEL005386] | | | | | | | |  |  |  |  |  |
| AAEL013032 | 21.9285 | 2.48265 | -3.14285 |  | |  |  |  |  |  |  |  |  |  |  |  |
| AAEL013033 | 21.9285 | 2.48265 | -3.14285 |  | |  |  |  |  |  |  |  |  |  |  |  |
| AAEL007381 | 24.4592 | 2.77723 | -3.13866 |  | |  |  |  |  |  |  |  |  |  |  |  |
| AAEL001390 | 180.886 | 20.5615 | -3.13706 |  | |  |  |  |  |  |  |  |  |  |  |  |
| AAEL002675 | 57.6258 | 6.58587 | -3.12927 | arginase [Source:VB External Description;Acc:AAEL002675] | | | | | |  |  |  |  |  |  |  |
| AAEL013077 | 12.3037 | 1.41253 | -3.12274 |  | |  |  |  |  |  |  |  |  |  |  |  |
| AAEL007055 | 36.5586 | 4.20061 | -3.12154 | lipase [Source:VB External Description;Acc:AAEL007055] | | | | | |  |  |  |  |  |  |  |
| AAEL007542 | 23.1068 | 2.65624 | -3.12086 | glutamate decarboxylase [Source:VB External Description;Acc:AAEL007542] | | | | | | | |  |  |  |  |  |
| AAEL007807 | 11.0986 | 1.2766 | -3.12 | cytochrome P450 (CYP4D38) [Source:VB Community Annotation;Acc:AAEL007807] | | | | | | | |  |  |  |  |  |
| AAEL000099 | 16.9047 | 1.97364 | -3.09849 | Clip-Domain Serine Protease family B. [Source:VB Community Annotation;Acc:AAEL000099] | | | | | | | | |  |  |  |  |
| AAEL013158 | 12.1954 | 1.43052 | -3.09173 | 40S ribosomal protein S3a [Source:VB Community Annotation;Acc:AAEL013158] | | | | | | | |  |  |  |  |  |
| AAEL012357 | 98.4281 | 11.5636 | -3.08948 |  | |  |  |  |  |  |  |  |  |  |  |  |
| AAEL003625 | 104.585 | 12.2934 | -3.08871 | Clip-Domain Serine Protease family B. [Source:VB Community Annotation;Acc:AAEL003625] | | | | | | | | |  |  |  |  |
| AAEL013266 | 21.3692 | 2.51339 | -3.08783 |  | |  |  |  |  |  |  |  |  |  |  |  |
| AAEL006962 | 50.0413 | 5.89063 | -3.08663 |  | |  |  |  |  |  |  |  |  |  |  |  |
| AAEL002046 | 143.54 | 16.9321 | -3.08362 | cytochrome P450 (CYP6CB1) [Source:VB Community Annotation;Acc:AAEL002046] | | | | | | | |  |  |  |  |  |
| AAEL010242 | 467.302 | 55.2099 | -3.08136 |  | |  |  |  |  |  |  |  |  |  |  |  |
| AAEL003614 | 7.5889 | 0.897768 | -3.07948 | Clip-Domain Serine Protease family B. [Source:VB Community Annotation;Acc:AAEL003614] | | | | | | | | |  |  |  |  |
| AAEL014551 | 3.56172 | 0.422483 | -3.07561 | triacylglycerol lipase, pancreatic [Source:VB External Description;Acc:AAEL014551] | | | | | | | |  |  |  |  |  |
| AAEL001771 | 0.739386 | 0.0877794 | -3.07437 | toll [Source:VB External Description;Acc:AAEL001771] | | | | | |  |  |  |  |  |  |  |
| AAEL003298 | 55.5484 | 6.59516 | -3.07426 | alkaline phosphatase [Source:VB External Description;Acc:AAEL003298] | | | | | | |  |  |  |  |  |  |
| AAEL005030 | 1.47249 | 0.174873 | -3.07388 | multidrug resistance protein 1 (ATP-binding cassette C1) [Source:VB External Description;Acc:AAEL005030] | | | | | | | | | |  |  |  |
| AAEL011624 | 50.7651 | 6.0373 | -3.07186 | granzyme A precursor, putative [Source:VB External Description;Acc:AAEL011624] | | | | | | | |  |  |  |  |  |
| AAEL011007 | 3.45119 | 0.410672 | -3.07104 | fibrinogen and fibronectin [Source:VB External Description;Acc:AAEL011007] | | | | | | | |  |  |  |  |  |
| AAEL001877 | 1.90453 | 0.227031 | -3.06848 | fucosyltransferase 11 (fut11) [Source:VB External Description;Acc:AAEL001877] | | | | | | | |  |  |  |  |  |
| AAEL000733 | 6.21907 | 0.750665 | -3.05046 | hydroxysteroid dehydrogenase [Source:VB External Description;Acc:AAEL000733] | | | | | | | |  |  |  |  |  |
| AAEL009121 | 7.19245 | 0.8708 | -3.04607 | cytochrome P450 [Source:VB Community Annotation;Acc:AAEL009121] | | | | | | |  |  |  |  |  |  |
| AAEL014005 | 5.17046 | 0.62683 | -3.04415 | clip-domain serine protease, putative [Source:VB External Description;Acc:AAEL014005] | | | | | | | | |  |  |  |  |
| AAEL001882 | 3.03935 | 0.370082 | -3.03785 |  | |  |  |  |  |  |  |  |  |  |  |  |
| AAEL001965 | 56.8105 | 6.92025 | -3.03726 | imaginal disc growth factor [Source:VB External Description;Acc:AAEL001965] | | | | | | | |  |  |  |  |  |
| AAEL017052 | 9.42798 | 1.14943 | -3.03603 |  | |  |  |  |  |  |  |  |  |  |  |  |
| AAEL003703 | 1.49105 | 0.181989 | -3.0344 | scarlet protein [Source:VB External Description;Acc:AAEL003703] | | | | | | |  |  |  |  |  |  |
| AAEL017835 | 19.9671 | 2.44779 | -3.02807 | 18S_rRNA [Source:RNAmmer;Acc:18S_rRNA] | | | | |  |  |  |  |  |  |  |  |
| AAEL008625 | 1.60609 | 0.197083 | -3.02668 | ABC transporter [Source:VB External Description;Acc:AAEL008625] | | | | | | |  |  |  |  |  |  |
| AAEL002920 | 42.9807 | 5.27536 | -3.02635 |  | |  |  |  |  |  |  |  |  |  |  |  |
| AAEL005687 | 4.1718 | 0.512243 | -3.02577 | protein serine/threonine kinase, putative [Source:VB External Description;Acc:AAEL005687] | | | | | | | | |  |  |  |  |
| AAEL010325 | 37.8953 | 4.65489 | -3.0252 |  | |  |  |  |  |  |  |  |  |  |  |  |
| AAEL004987 | 47.052 | 5.78372 | -3.02418 |  | |  |  |  |  |  |  |  |  |  |  |  |
| AAEL000563 | 9.41468 | 1.15851 | -3.02264 | C-Type Lectin (CTL) - mannose binding. [Source:VB Community Annotation;Acc:AAEL000563] | | | | | | | | |  |  |  |  |
| AAEL012128 | 3.64563 | 0.448979 | -3.02145 | cationic amino acid transporter [Source:VB External Description;Acc:AAEL012128] | | | | | | | |  |  |  |  |  |
| AAEL007027 | 4.23402 | 0.522003 | -3.0199 | sodium/solute symporter [Source:VB External Description;Acc:AAEL007027] | | | | | | | |  |  |  |  |  |
| AAEL009875 | 19.5309 | 2.4096 | -3.0189 | alanine aminotransferase [Source:VB External Description;Acc:AAEL009875] | | | | | | | |  |  |  |  |  |
| AAEL002022 | 13.9829 | 1.73698 | -3.00902 | protein serine/threonine kinase, putative [Source:VB External Description;Acc:AAEL002022] | | | | | | | | |  |  |  |  |
| AAEL002546 | 8.29876 | 1.03319 | -3.0058 |  | |  |  |  |  |  |  |  |  |  |  |  |
| AAEL005586 | 83.2498 | 10.3773 | -3.00401 |  | |  |  |  |  |  |  |  |  |  |  |  |
| AAEL005575 | 83.2498 | 10.3773 | -3.00401 |  | |  |  |  |  |  |  |  |  |  |  |  |
| AAEL000037 | 62.4727 | 7.78835 | -3.00384 | Clip-Domain Serine Protease family B. [Source:VB Community Annotation;Acc:AAEL000037] | | | | | | | | |  |  |  |  |
| AAEL012646 | 6.74266 | 0.844756 | -2.99671 |  | |  |  |  |  |  |  |  |  |  |  |  |
| AAEL004341 | 8.26397 | 1.03588 | -2.99598 | Carboxy/choline esterase Alpha Esterase [Source:VB Community Annotation;Acc:AAEL004341] | | | | | | | | |  |  |  |  |
| AAEL003114 | 11.7396 | 1.48057 | -2.98716 |  | |  |  |  |  |  |  |  |  |  |  |  |
| AAEL010379 | 9.42975 | 1.18953 | -2.98682 | ATP-binding cassette transporter [Source:VB External Description;Acc:AAEL010379] | | | | | | | | |  |  |  |  |
| AAEL018207 | 1.33274 | 0.168306 | -2.98524 | C-Type lectin (CTL) [Source:VB Community Annotation;Acc:AAEL018207] | | | | | | | |  |  |  |  |  |
| AAEL001868 | 0.918116 | 0.115973 | -2.98489 |  | |  |  |  |  |  |  |  |  |  |  |  |
| AAEL012853 | 31.3969 | 3.96703 | -2.98449 |  | |  |  |  |  |  |  |  |  |  |  |  |
| AAEL010235 | 1052.07 | 133.023 | -2.98348 | 30 kDa salivary gland allergen Aed a 3 Precursor (Allergen Aed a 3) [Source:VB External Description;Acc:AAEL010235] | | | | | | | | | | |  |  |
| AAEL010511 | 5.28295 | 0.668764 | -2.98177 |  | |  |  |  |  |  |  |  |  |  |  |  |
| AAEL002467 | 69.5003 | 8.80555 | -2.98053 |  | |  |  |  |  |  |  |  |  |  |  |  |
| AAEL010304 | 5.25863 | 0.66909 | -2.97441 |  | |  |  |  |  |  |  |  |  |  |  |  |
| AAEL010789 | 30.5627 | 3.88902 | -2.97429 |  | |  |  |  |  |  |  |  |  |  |  |  |
| AAEL009520 | 43.8642 | 5.59189 | -2.97164 | leucine-rich immune protein (Long) [Source:VB Community Annotation;Acc:AAEL009520] | | | | | | | | |  |  |  |  |
| AAEL000748 | 10.9107 | 1.39247 | -2.97002 |  | |  |  |  |  |  |  |  |  |  |  |  |
| AAEL001899 | 4.27257 | 0.546081 | -2.96792 | juvenile hormone-inducible protein, putative [Source:VB External Description;Acc:AAEL001899] | | | | | | | | |  |  |  |  |
| AAEL000726 | 31.4368 | 4.02534 | -2.96527 | fibrinogen and fibronectin [Source:VB External Description;Acc:AAEL000726] | | | | | | | |  |  |  |  |  |
| AAEL005988 | 2.86862 | 0.368293 | -2.96143 | C-Type Lysozyme (multilysozyme domain protein). [Source:VB Community Annotation;Acc:AAEL005988] | | | | | | | | | |  |  |  |
| AAEL000438 | 12.8832 | 1.65509 | -2.96051 | epoxide hydrolase [Source:VB External Description;Acc:AAEL000438] | | | | | | |  |  |  |  |  |  |
| AAEL008144 | 43.7873 | 5.64817 | -2.95466 | AMP dependent ligase [Source:VB External Description;Acc:AAEL008144] | | | | | | | |  |  |  |  |  |
| AAEL010228 | 309.484 | 39.922 | -2.95461 |  | |  |  |  |  |  |  |  |  |  |  |  |
| AAEL017188 | 1.63803 | 0.211388 | -2.95399 |  | |  |  |  |  |  |  |  |  |  |  |  |
| AAEL002457 | 104.98 | 13.5598 | -2.9527 |  | |  |  |  |  |  |  |  |  |  |  |  |
| AAEL003899 | 56.4748 | 7.30626 | -2.9504 | sugar transporter [Source:VB External Description;Acc:AAEL003899] | | | | | | |  |  |  |  |  |  |
| AAEL010655 | 0.683246 | 0.0884475 | -2.94951 | Class A Scavenger Receptor (SRCR domain) with Serine Protease domain. [Source:VB Community Annotation;Acc:AAEL010655] | | | | | | | | | | | |  |
| AAEL011810 | 20.4421 | 2.65681 | -2.94378 |  | |  |  |  |  |  |  |  |  |  |  |  |
| AAEL013990 | 5.84594 | 0.760181 | -2.94302 | hexamerin 2 beta [Source:VB External Description;Acc:AAEL013990] | | | | | | |  |  |  |  |  |  |
| AAEL006130 | 33.685 | 4.39547 | -2.93802 |  | |  |  |  |  |  |  |  |  |  |  |  |
| AAEL003584 | 3.35499 | 0.437805 | -2.93795 |  | |  |  |  |  |  |  |  |  |  |  |  |
| AAEL012953 | 1.83292 | 0.239211 | -2.93779 |  | |  |  |  |  |  |  |  |  |  |  |  |
| AAEL003816 | 187.72 | 24.5172 | -2.93671 |  | |  |  |  |  |  |  |  |  |  |  |  |
| AAEL003116 | 997.521 | 130.385 | -2.93557 | phosrestin i (arrestin b) (arrestin 2) [Source:VB External Description;Acc:AAEL003116] | | | | | | | | |  |  |  |  |
| AAEL010154 | 7.2444 | 0.948469 | -2.93319 | cytochrome P450 [Source:VB Community Annotation;Acc:AAEL010154] | | | | | | |  |  |  |  |  |  |
| AAEL005349 | 33.3485 | 4.37893 | -2.92897 |  | |  |  |  |  |  |  |  |  |  |  |  |
| AAEL004951 | 3.52231 | 0.463246 | -2.92667 |  | |  |  |  |  |  |  |  |  |  |  |  |
| AAEL014663 | 3.65051 | 0.481137 | -2.92358 | AMP dependent coa ligase [Source:VB External Description;Acc:AAEL014663] | | | | | | | |  |  |  |  |  |
| AAEL003079 | 5.85259 | 0.771797 | -2.92278 | glucosyl/glucuronosyl transferases [Source:VB External Description;Acc:AAEL003079] | | | | | | | | |  |  |  |  |
| AAEL008028 | 14.4746 | 1.91093 | -2.92117 | monocarboxylate transporter [Source:VB External Description;Acc:AAEL008028] | | | | | | | |  |  |  |  |  |
| AAEL011408 | 48.2235 | 6.36714 | -2.92102 | C-Type Lectin (CTL21) [Source:VB Community Annotation;Acc:AAEL011408] | | | | | | | |  |  |  |  |  |
| AAEL002880 | 20.8159 | 2.74981 | -2.92028 |  | |  |  |  |  |  |  |  |  |  |  |  |
| AAEL007006 | 7.98877 | 1.05649 | -2.9187 | Clip-Domain Serine Protease family A. [Source:VB Community Annotation;Acc:AAEL007006] | | | | | | | | |  |  |  |  |
| AAEL007665 | 21.6219 | 2.86575 | -2.91551 |  | |  |  |  |  |  |  |  |  |  |  |  |
| AAEL005829 | 11.9229 | 1.58368 | -2.91238 |  | |  |  |  |  |  |  |  |  |  |  |  |
| AAEL004181 | 6.36134 | 0.845704 | -2.9111 |  | |  |  |  |  |  |  |  |  |  |  |  |
| AAEL009018 | 117.864 | 15.7119 | -2.90719 | cytochrome P450 [Source:VB External Description;Acc:AAEL009018] | | | | | | |  |  |  |  |  |  |
| AAEL006417 | 259.779 | 34.6444 | -2.90659 | D7 protein, putative [Source:VB External Description;Acc:AAEL006417] | | | | | | |  |  |  |  |  |  |
| AAEL009762 | 3.33593 | 0.444913 | -2.9065 | cytochrome P450 [Source:VB Community Annotation;Acc:AAEL009762] | | | | | | |  |  |  |  |  |  |
| AAEL015386 | 7.96002 | 1.06327 | -2.90427 | dipeptidyl-peptidase [Source:VB External Description;Acc:AAEL015386] | | | | | | |  |  |  |  |  |  |
| AAEL011454 | 3.18004 | 0.425272 | -2.90259 |  | |  |  |  |  |  |  |  |  |  |  |  |
| AAEL005747 | 34.6845 | 4.64093 | -2.9018 |  | |  |  |  |  |  |  |  |  |  |  |  |
| AAEL006649 | 2.52823 | 0.338792 | -2.89965 | tnf receptor associated factor [Source:VB External Description;Acc:AAEL006649] | | | | | | | |  |  |  |  |  |
| AAEL005120 | 12.4638 | 1.67049 | -2.89941 | zinc finger protein [Source:VB External Description;Acc:AAEL005120] | | | | | | |  |  |  |  |  |  |
| AAEL018317 | 1.43372 | 0.193281 | -2.89099 |  | |  |  |  |  |  |  |  |  |  |  |  |
| AAEL006586 | 2.90539 | 0.392342 | -2.88855 | serine protease [Source:VB External Description;Acc:AAEL006586] | | | | | | |  |  |  |  |  |  |
| AAEL009205 | 6.08903 | 0.823093 | -2.88708 |  | |  |  |  |  |  |  |  |  |  |  |  |
| AAEL010082 | 1.11408 | 0.150854 | -2.88463 |  | |  |  |  |  |  |  |  |  |  |  |  |
| AAEL003601 | 36.3656 | 4.92709 | -2.88377 |  | |  |  |  |  |  |  |  |  |  |  |  |
| AAEL000316 | 13.9554 | 1.89607 | -2.87974 |  | |  |  |  |  |  |  |  |  |  |  |  |
| AAEL001641 | 70.9424 | 9.6469 | -2.87851 | deoxyribonuclease I, putative [Source:VB External Description;Acc:AAEL001641] | | | | | | | |  |  |  |  |  |
| AAEL010979 | 33.1134 | 4.50398 | -2.87814 |  | |  |  |  |  |  |  |  |  |  |  |  |
| AAEL017278 | 33.1134 | 4.50398 | -2.87814 |  | |  |  |  |  |  |  |  |  |  |  |  |
| AAEL017688 | 8.09381 | 1.10378 | -2.87436 | 28S_rRNA [Source:RNAmmer;Acc:28S_rRNA] | | | | |  |  |  |  |  |  |  |  |
| AAEL003320 | 1.00122 | 0.136674 | -2.87295 |  | |  |  |  |  |  |  |  |  |  |  |  |
| AAEL006824 | 58.1095 | 7.94213 | -2.87118 | cytochrome P450 [Source:VB External Description;Acc:AAEL006824] | | | | | | |  |  |  |  |  |  |
| AAEL003642 | 16.1736 | 2.21057 | -2.87115 | serine protease [Source:VB External Description;Acc:AAEL003642] | | | | | | |  |  |  |  |  |  |
| AAEL011405 | 1.69941 | 0.232738 | -2.86826 |  | |  |  |  |  |  |  |  |  |  |  |  |
| AAEL018103 | 6.95532 | 0.952787 | -2.86789 |  | |  |  |  |  |  |  |  |  |  |  |  |
| AAEL004969 | 6.95532 | 0.952787 | -2.86789 |  | |  |  |  |  |  |  |  |  |  |  |  |
| AAEL013417 | 17.9967 | 2.46821 | -2.8662 | fibrinogen and fibronectin [Source:VB External Description;Acc:AAEL013417] | | | | | | | |  |  |  |  |  |
| AAEL003389 | 552.28 | 75.8072 | -2.86499 | attacin anti-microbial peptide [Source:VB Community Annotation;Acc:AAEL003389] | | | | | | | |  |  |  |  |  |
| AAEL017114 | 54.6529 | 7.53298 | -2.859 |  | |  |  |  |  |  |  |  |  |  |  |  |
| AAEL011898 | 35.6407 | 4.91271 | -2.85893 | 1-acyl-sn-glycerol-3-phosphate acyltransferase [Source:VB External Description;Acc:AAEL011898] | | | | | | | | | |  |  |  |
| AAEL001517 | 5.63853 | 0.778186 | -2.85713 | carboxylesterase [Source:VB External Description;Acc:AAEL001517] | | | | | | |  |  |  |  |  |  |
| AAEL002959 | 1.96426 | 0.271154 | -2.8568 | brain chitinase and chia [Source:VB External Description;Acc:AAEL002959] | | | | | | | |  |  |  |  |  |
| AAEL000229 | 192.309 | 26.6189 | -2.8529 | prosialokinin precursor [Source:VB External Description;Acc:AAEL000229] | | | | | | | |  |  |  |  |  |
| AAEL006630 | 4.67143 | 0.648039 | -2.84971 |  | |  |  |  |  |  |  |  |  |  |  |  |
| AAEL003279 | 13.5452 | 1.88049 | -2.8486 | clip-domain serine protease, putative [Source:VB External Description;Acc:AAEL003279] | | | | | | | | |  |  |  |  |
| AAEL005853 | 3.42939 | 0.47697 | -2.84598 | amino acid transporter [Source:VB External Description;Acc:AAEL005853] | | | | | | | |  |  |  |  |  |
| AAEL010128 | 16.5436 | 2.31743 | -2.83567 | leucine-rich immune protein (Long) [Source:VB Community Annotation;Acc:AAEL010128] | | | | | | | | |  |  |  |  |
| AAEL011820 | 5.37346 | 0.75502 | -2.83126 |  | |  |  |  |  |  |  |  |  |  |  |  |
| AAEL008093 | 108.686 | 15.3227 | -2.82642 | trypsin [Source:VB External Description;Acc:AAEL008093] | | | | | |  |  |  |  |  |  |  |
| AAEL005849 | 240.437 | 33.9231 | -2.82532 | synaptic vesicle protein [Source:VB External Description;Acc:AAEL005849] | | | | | | | |  |  |  |  |  |
| AAEL003156 | 5.37002 | 0.758339 | -2.82401 | fibrinogen and fibronectin [Source:VB External Description;Acc:AAEL003156] | | | | | | | |  |  |  |  |  |
| AAEL010656 | 9.00554 | 1.27231 | -2.82337 | leucine-rich immune protein (Short) [Source:VB Community Annotation;Acc:AAEL010656] | | | | | | | | |  |  |  |  |
| AAEL002347 | 6.72089 | 0.951372 | -2.82057 | serine-type enodpeptidase, [Source:VB External Description;Acc:AAEL002347] | | | | | | | |  |  |  |  |  |
| AAEL005401 | 290.326 | 41.3602 | -2.81136 |  | |  |  |  |  |  |  |  |  |  |  |  |
| AAEL017262 | 17.4706 | 2.48915 | -2.8112 |  | |  |  |  |  |  |  |  |  |  |  |  |
| AAEL004786 | 4.62701 | 0.659649 | -2.81031 |  | |  |  |  |  |  |  |  |  |  |  |  |
| AAEL007378 | 2.59751 | 0.370738 | -2.80866 |  | |  |  |  |  |  |  |  |  |  |  |  |
| AAEL018691 | 181.996 | 26.0354 | -2.80536 |  | |  |  |  |  |  |  |  |  |  |  |  |
| AAEL004899 | 175.028 | 25.0618 | -2.80402 |  | |  |  |  |  |  |  |  |  |  |  |  |
| AAEL003413 | 21.7903 | 3.12139 | -2.80343 | F-spondin [Source:VB External Description;Acc:AAEL003413] | | | | | | |  |  |  |  |  |  |
| AAEL013180 | 13.1128 | 1.87866 | -2.8032 |  | |  |  |  |  |  |  |  |  |  |  |  |
| AAEL008108 | 17.9848 | 2.57873 | -2.80205 | guanine nucleotide-binding protein beta 2 (g protein beta2) [Source:VB External Description;Acc:AAEL008108] | | | | | | | | | |  |  |  |
| AAEL007294 | 15.8687 | 2.27789 | -2.80042 |  | |  |  |  |  |  |  |  |  |  |  |  |
| AAEL004717 | 2.18591 | 0.313955 | -2.7996 | cyclin-dependent kinase 5 activator [Source:VB External Description;Acc:AAEL004717] | | | | | | | | |  |  |  |  |
| AAEL006423 | 326.124 | 46.841 | -2.79958 |  | |  |  |  |  |  |  |  |  |  |  |  |
| AAEL007942 | 273.098 | 39.2312 | -2.79935 | fibrinogen and fibronectin [Source:VB External Description;Acc:AAEL007942] | | | | | | | |  |  |  |  |  |
| AAEL004278 | 139.078 | 19.9847 | -2.79892 |  | |  |  |  |  |  |  |  |  |  |  |  |
| AAEL017245 | 159.377 | 22.9527 | -2.79571 |  | |  |  |  |  |  |  |  |  |  |  |  |
| AAEL003047 | 3.79507 | 0.546547 | -2.79571 | netrin [Source:VB External Description;Acc:AAEL003047] | | | | | |  |  |  |  |  |  |  |
| AAEL003568 | 6.10857 | 0.880854 | -2.79386 | threonine dehydratase/deaminase [Source:VB External Description;Acc:AAEL003568] | | | | | | | | |  |  |  |  |
| AAEL000277 | 13.8989 | 2.00777 | -2.7913 |  | |  |  |  |  |  |  |  |  |  |  |  |
| AAEL010366 | 37.3938 | 5.40726 | -2.78983 | glucosyl/glucuronosyl transferases [Source:VB External Description;Acc:AAEL010366] | | | | | | | | |  |  |  |  |
| AAEL011455 | 40.4274 | 5.85297 | -2.78809 | C-Type Lectin (CTLMA12) - mannose binding [Source:VB Community Annotation;Acc:AAEL011455] | | | | | | | | | |  |  |  |
| AAEL003029 | 18.5945 | 2.69273 | -2.78774 |  | |  |  |  |  |  |  |  |  |  |  |  |
| AAEL013535 | 485.307 | 70.3232 | -2.78682 | phosrestin ii (arrestin a) (arrestin 1) [Source:VB External Description;Acc:AAEL013535] | | | | | | | | |  |  |  |  |
| AAEL003848 | 218.942 | 31.7374 | -2.78629 |  | |  |  |  |  |  |  |  |  |  |  |  |
| AAEL000556 | 153.7 | 22.2825 | -2.78613 | C-Type Lectin (CTL25) [Source:VB Community Annotation;Acc:AAEL000556] | | | | | | | |  |  |  |  |  |
| AAEL007818 | 16617.7 | 2410.55 | -2.78528 | Trypsin 3A1 Precursor (EC 3.4.21.4) [Source:VB External Description;Acc:AAEL007818] | | | | | | | | |  |  |  |  |
| AAEL008668 | 5.25944 | 0.764561 | -2.78221 | Clip-Domain Serine Protease family B. [Source:VB Community Annotation;Acc:AAEL008668] | | | | | | | | |  |  |  |  |
| AAEL013271 | 5.1335 | 0.747965 | -2.7789 | organic cation transporter [Source:VB External Description;Acc:AAEL013271] | | | | | | | |  |  |  |  |  |
| AAEL002971 | 42.2496 | 6.15712 | -2.77861 | sarcalumenin [Source:VB External Description;Acc:AAEL002971] | | | | | | |  |  |  |  |  |  |
| AAEL017309 | 14.9887 | 2.18516 | -2.77807 |  | |  |  |  |  |  |  |  |  |  |  |  |
| AAEL004000 | 1.86023 | 0.271602 | -2.77591 | Toll-like receptor [Source:VB Community Annotation;Acc:AAEL004000] | | | | | | |  |  |  |  |  |  |
| AAEL007371 | 70.7896 | 10.3415 | -2.7751 |  | |  |  |  |  |  |  |  |  |  |  |  |
| AAEL008802 | 383.695 | 56.3566 | -2.7673 |  | |  |  |  |  |  |  |  |  |  |  |  |
| AAEL004418 | 11.9624 | 1.76084 | -2.76417 |  | |  |  |  |  |  |  |  |  |  |  |  |
| AAEL000729 | 4.88844 | 0.719588 | -2.76413 |  | |  |  |  |  |  |  |  |  |  |  |  |
| AAEL003655 | 1.67291 | 0.246397 | -2.76331 |  | |  |  |  |  |  |  |  |  |  |  |  |
| AAEL017018 | 53.2267 | 7.84262 | -2.76274 |  | |  |  |  |  |  |  |  |  |  |  |  |
| AAEL008784 | 34.5947 | 5.11104 | -2.75886 | serine-type enodpeptidase, [Source:VB External Description;Acc:AAEL008784] | | | | | | | |  |  |  |  |  |
| AAEL002853 | 4.48803 | 0.663516 | -2.75788 | ccaat/enhancer binding protein [Source:VB External Description;Acc:AAEL002853] | | | | | | | |  |  |  |  |  |
| AAEL007416 | 4.0793 | 0.60407 | -2.75554 | cysteine dioxygenase [Source:VB External Description;Acc:AAEL007416] | | | | | | |  |  |  |  |  |  |
| AAEL004692 | 2.91141 | 0.431373 | -2.75471 | otopetrin [Source:VB External Description;Acc:AAEL004692] | | | | | |  |  |  |  |  |  |  |
| AAEL013545 | 168.154 | 24.94 | -2.75325 |  | |  |  |  |  |  |  |  |  |  |  |  |
| AAEL012060 | 36.5674 | 5.44305 | -2.74807 | PBAN-type neuropeptides Precursor (Pheromone/pyrokinin biosynthesis-activating neuropeptide) [Source:VB External Description;Acc:AAEL012060] | | | | | | | | | | | | |
| AAEL005392 | 2.53162 | 0.376885 | -2.74786 | dihydropyridine-sensitive l-type calcium channel [Source:VB External Description;Acc:AAEL005392] | | | | | | | | | |  |  |  |
| AAEL000294 | 142.209 | 21.2045 | -2.74557 |  | |  |  |  |  |  |  |  |  |  |  |  |
| AAEL002672 | 1.55706 | 0.232948 | -2.74075 | matrix metalloproteinase [Source:VB External Description;Acc:AAEL002672] | | | | | | | |  |  |  |  |  |
| AAEL003898 | 18.9763 | 2.84113 | -2.73966 |  | |  |  |  |  |  |  |  |  |  |  |  |
| AAEL010751 | 49.1906 | 7.36497 | -2.73963 |  | |  |  |  |  |  |  |  |  |  |  |  |
| AAEL010758 | 49.1906 | 7.36497 | -2.73963 |  | |  |  |  |  |  |  |  |  |  |  |  |
| AAEL005533 | 3.63863 | 0.545408 | -2.73799 | synaptic vesicle protein [Source:VB External Description;Acc:AAEL005533] | | | | | | | |  |  |  |  |  |
| AAEL012886 | 5.35739 | 0.803683 | -2.73683 |  | |  |  |  |  |  |  |  |  |  |  |  |
| AAEL003590 | 815.919 | 122.454 | -2.73619 |  | |  |  |  |  |  |  |  |  |  |  |  |
| AAEL000714 | 24.6579 | 3.70121 | -2.73598 | sodium-dependent excitatory amino acid transporter [Source:VB External Description;Acc:AAEL000714] | | | | | | | | | |  |  |  |
| AAEL018223 | 0.549787 | 0.0826457 | -2.73386 |  | |  |  |  |  |  |  |  |  |  |  |  |
| AAEL003934 | 29.7843 | 4.47795 | -2.73364 |  | |  |  |  |  |  |  |  |  |  |  |  |
| AAEL006347 | 152.655 | 22.9525 | -2.73355 | Apyrase Precursor (EC 3.6.1.5)(Adenosine diphosphatase)(ATP-diphosphohydrolase)(ATP-diphosphatase)(ADPase)(Allergen Aed a 1) [Source:VB Community Annotation;Acc:AAEL006347] | | | | | | | | | | | | |
| AAEL001457 | 9.90305 | 1.49119 | -2.73141 | guanylatte cyclase alpha 1 subunit [Source:VB External Description;Acc:AAEL001457] | | | | | | | | |  |  |  |  |
| AAEL014583 | 9403.75 | 1416.95 | -2.73045 | 60S acidic ribosomal protein P2 [Source:VB Community Annotation;Acc:AAEL014583] | | | | | | | |  |  |  |  |  |
| AAEL003611 | 11.8504 | 1.78776 | -2.72871 | stearoyl-coa desaturase [Source:VB External Description;Acc:AAEL003611] | | | | | | | |  |  |  |  |  |
| AAEL005200 | 3.59044 | 0.541731 | -2.72851 |  | |  |  |  |  |  |  |  |  |  |  |  |
| AAEL006886 | 5.5342 | 0.835482 | -2.72769 |  | |  |  |  |  |  |  |  |  |  |  |  |
| AAEL000793 | 568.492 | 86.0667 | -2.72361 | venom allergen [Source:VB External Description;Acc:AAEL000793] | | | | | | |  |  |  |  |  |  |
| AAEL013623 | 51.9582 | 7.86924 | -2.72306 | trypsin [Source:VB External Description;Acc:AAEL013623] | | | | | |  |  |  |  |  |  |  |
| AAEL001835 | 4.8316 | 0.733397 | -2.71984 | sodium/solute symporter [Source:VB External Description;Acc:AAEL001835] | | | | | | | |  |  |  |  |  |
| AAEL006424 | 326.35 | 49.6211 | -2.71739 | 37 kDa salivary gland allergen Aed a 2 Precursor (Protein D7)(Allergen Aed a 2) [Source:VB Community Annotation;Acc:AAEL006424] | | | | | | | | | | | |  |
| AAEL004249 | 90.3388 | 13.751 | -2.71581 |  | |  |  |  |  |  |  |  |  |  |  |  |
| AAEL002585 | 13.7424 | 2.0918 | -2.71581 | serine protease [Source:VB External Description;Acc:AAEL002585] | | | | | | |  |  |  |  |  |  |
| AAEL013656 | 62.7259 | 9.55159 | -2.71525 | bm-40 precursor [Source:VB External Description;Acc:AAEL013656] | | | | | | |  |  |  |  |  |  |
| AAEL010462 | 2.30097 | 0.350381 | -2.71524 | vesamicol binding protein, putative [Source:VB External Description;Acc:AAEL010462] | | | | | | | | |  |  |  |  |
| AAEL003585 | 32.7428 | 4.99051 | -2.71392 |  | |  |  |  |  |  |  |  |  |  |  |  |
| AAEL009927 | 7.89907 | 1.20696 | -2.7103 |  | |  |  |  |  |  |  |  |  |  |  |  |
| AAEL006498 | 2827.1 | 432.162 | -2.70968 | long wavelength sensitive opsin [Source:VB Community Annotation;Acc:AAEL006498] | | | | | | | |  |  |  |  |  |
| AAEL009852 | 79.5508 | 12.1606 | -2.70966 |  | |  |  |  |  |  |  |  |  |  |  |  |
| AAEL005995 | 51.5314 | 7.90722 | -2.70421 |  | |  |  |  |  |  |  |  |  |  |  |  |
| AAEL007105 | 3.52618 | 0.541554 | -2.70293 |  | |  |  |  |  |  |  |  |  |  |  |  |
| AAEL007126 | 12.0448 | 1.85068 | -2.70229 | sugar transporter [Source:VB External Description;Acc:AAEL007126] | | | | | | |  |  |  |  |  |  |
| AAEL007778 | 131.042 | 20.1531 | -2.70096 | leucine-rich transmembrane protein [Source:VB External Description;Acc:AAEL007778] | | | | | | | | |  |  |  |  |
| AAEL006602 | 73.2675 | 11.2701 | -2.70068 |  | |  |  |  |  |  |  |  |  |  |  |  |
| AAEL009489 | 0.912661 | 0.140564 | -2.69885 | forkhead box protein (AaegFOXM1) [Source:VB External Description;Acc:AAEL009489] | | | | | | | | |  |  |  |  |
| AAEL003035 | 291.909 | 45.0216 | -2.69683 | short wavelength sensitive opsin [Source:VB Community Annotation;Acc:AAEL003035] | | | | | | | | |  |  |  |  |
| AAEL005112 | 7.54772 | 1.16434 | -2.69653 | Carboxy/choline esterase Alpha Esterase [Source:VB Community Annotation;Acc:AAEL005112] | | | | | | | | |  |  |  |  |
| AAEL005437 | 8.7241 | 1.34802 | -2.69417 | transient receptor potential channel [Source:VB External Description;Acc:AAEL005437] | | | | | | | | |  |  |  |  |
| AAEL009844 | 87.8461 | 13.6092 | -2.69039 |  | |  |  |  |  |  |  |  |  |  |  |  |
| AAEL001241 | 6.89354 | 1.07002 | -2.68761 |  | |  |  |  |  |  |  |  |  |  |  |  |
| AAEL000640 | 9.94813 | 1.54814 | -2.68389 | alanine-glyoxylate aminotransferase [Source:VB External Description;Acc:AAEL000640] | | | | | | | | |  |  |  |  |
| AAEL007489 | 11.4804 | 1.78671 | -2.68379 | synaptic vesicle protein [Source:VB External Description;Acc:AAEL007489] | | | | | | | |  |  |  |  |  |
| AAEL008936 | 19.3419 | 3.01199 | -2.68294 |  | |  |  |  |  |  |  |  |  |  |  |  |
| AAEL005768 | 72.4835 | 11.2923 | -2.68231 |  | |  |  |  |  |  |  |  |  |  |  |  |
| AAEL008632 | 11.2769 | 1.7614 | -2.67857 | ABC transporter [Source:VB External Description;Acc:AAEL008632] | | | | | | |  |  |  |  |  |  |
| AAEL002185 | 5.05914 | 0.790678 | -2.67773 | cuticle protein, putative [Source:VB External Description;Acc:AAEL002185] | | | | | | | |  |  |  |  |  |
| AAEL008160 | 9.82165 | 1.538 | -2.67491 | fatty acid synthase [Source:VB External Description;Acc:AAEL008160] | | | | | | |  |  |  |  |  |  |
| AAEL002606 | 8.15202 | 1.27714 | -2.67424 | odorant binding protein OBP35 [Source:VB Community Annotation;Acc:AAEL002606] | | | | | | | |  |  |  |  |  |
| AAEL007808 | 9.20323 | 1.44496 | -2.67111 | cytochrome P450 (CYP4D39) [Source:VB Community Annotation;Acc:AAEL007808] | | | | | | | |  |  |  |  |  |
| AAEL011366 | 3.5441 | 0.556504 | -2.67095 |  | |  |  |  |  |  |  |  |  |  |  |  |
| AAEL005672 | 39.3348 | 6.18193 | -2.66968 | adenosine deaminase [Source:VB External Description;Acc:AAEL005672] | | | | | | |  |  |  |  |  |  |
| AAEL004188 | 165.386 | 26.0364 | -2.66724 |  | |  |  |  |  |  |  |  |  |  |  |  |
| AAEL009842 | 51.4815 | 8.11064 | -2.66616 | galectin [Source:VB Community Annotation;Acc:AAEL009842] | | | | | | |  |  |  |  |  |  |
| AAEL003053 | 160.031 | 25.2133 | -2.6661 | allergen, putative [Source:VB External Description;Acc:AAEL003053] | | | | | | |  |  |  |  |  |  |
| AAEL002811 | 310.025 | 48.8983 | -2.66453 |  | |  |  |  |  |  |  |  |  |  |  |  |
| AAEL002194 | 41.4284 | 6.54519 | -2.66211 | uricase [Source:VB External Description;Acc:AAEL002194] | | | | | |  |  |  |  |  |  |  |
| AAEL003078 | 27.9588 | 4.4177 | -2.66194 |  | |  |  |  |  |  |  |  |  |  |  |  |
| AAEL017517 | 236.897 | 37.443 | -2.66149 |  | |  |  |  |  |  |  |  |  |  |  |  |
| AAEL008855 | 0.972242 | 0.15376 | -2.66064 | dynein heavy chain [Source:VB External Description;Acc:AAEL008855] | | | | | | |  |  |  |  |  |  |
| AAEL017904 | 814.768 | 128.954 | -2.65954 | 5.8S_rRNA [Source:RNAmmer;Acc:5.8S_rRNA] | | | | |  |  |  |  |  |  |  |  |
| AAEL003299 | 58.7174 | 9.30456 | -2.65778 |  | |  |  |  |  |  |  |  |  |  |  |  |
| AAEL001998 | 75.8768 | 12.0443 | -2.65531 |  | |  |  |  |  |  |  |  |  |  |  |  |
| AAEL015076 | 19.1225 | 3.04394 | -2.65126 | uncharacerized [Source:VB External Description;Acc:AAEL015076] | | | | | | |  |  |  |  |  |  |
| AAEL005102 | 9.0622 | 1.4452 | -2.64859 |  | |  |  |  |  |  |  |  |  |  |  |  |
| AAEL013001 | 3.67223 | 0.585685 | -2.64846 |  | |  |  |  |  |  |  |  |  |  |  |  |
| AAEL009421 | 7.35381 | 1.17315 | -2.64811 | cyclophilin-r [Source:VB External Description;Acc:AAEL009421] | | | | | | |  |  |  |  |  |  |
| AAEL015200 | 2.77153 | 0.442468 | -2.64704 |  | |  |  |  |  |  |  |  |  |  |  |  |
| AAEL003716 | 84.6157 | 13.5378 | -2.64394 | ribonuclease UK114, putative [Source:VB External Description;Acc:AAEL003716] | | | | | | | |  |  |  |  |  |
| AAEL018266 | 4.02802 | 0.646445 | -2.63947 |  | |  |  |  |  |  |  |  |  |  |  |  |
| AAEL000119 | 3.01269 | 0.484107 | -2.63766 | AMP dependent coa ligase [Source:VB External Description;Acc:AAEL000119] | | | | | | | |  |  |  |  |  |
| AAEL001529 | 2.97355 | 0.47835 | -2.63605 |  | |  |  |  |  |  |  |  |  |  |  |  |
| AAEL002234 | 0.828509 | 0.133347 | -2.63534 |  | |  |  |  |  |  |  |  |  |  |  |  |
| AAEL006281 | 3.07998 | 0.496014 | -2.63447 | glucose transporter (sugar transporter [Source:VB External Description;Acc:AAEL006281] | | | | | | | | |  |  |  |  |
| AAEL005675 | 13.0419 | 2.10096 | -2.63403 | clip-domain serine protease, putative [Source:VB External Description;Acc:AAEL005675] | | | | | | | | |  |  |  |  |
| AAEL005026 | 0.941729 | 0.15171 | -2.634 | ATP-dependent bile acid permease [Source:VB External Description;Acc:AAEL005026] | | | | | | | | |  |  |  |  |
| AAEL003467 | 101.652 | 16.3782 | -2.63379 |  | |  |  |  |  |  |  |  |  |  |  |  |
| AAEL010980 | 6.61382 | 1.06603 | -2.63324 | GPCR Methuselah Family [Source:VB Community Annotation;Acc:AAEL010980] | | | | | | | |  |  |  |  |  |
| AAEL002417 | 344.092 | 55.4807 | -2.63274 | troponin t, invertebrate [Source:VB External Description;Acc:AAEL002417] | | | | | | | |  |  |  |  |  |
| AAEL006284 | 7.46182 | 1.20344 | -2.63237 |  | |  |  |  |  |  |  |  |  |  |  |  |
| AAEL018308 | 4.23214 | 0.683228 | -2.63095 |  | |  |  |  |  |  |  |  |  |  |  |  |
| AAEL006624 | 6.24366 | 1.00851 | -2.63017 |  | |  |  |  |  |  |  |  |  |  |  |  |
| AAEL005815 | 14.9604 | 2.41955 | -2.62834 | vitellogenin, putative [Source:VB External Description;Acc:AAEL005815] | | | | | | |  |  |  |  |  |  |
| AAEL010162 | 3.85216 | 0.624068 | -2.62589 |  | |  |  |  |  |  |  |  |  |  |  |  |
| AAEL002018 | 17.0775 | 2.76782 | -2.62527 |  | |  |  |  |  |  |  |  |  |  |  |  |
| AAEL008773 | 3.67463 | 0.59635 | -2.62337 | laminin A chain, putative [Source:VB External Description;Acc:AAEL008773] | | | | | | | |  |  |  |  |  |
| AAEL011941 | 2.16976 | 0.352215 | -2.62301 | oxidase/peroxidase [Source:VB External Description;Acc:AAEL011941] | | | | | | |  |  |  |  |  |  |
| AAEL012492 | 7.05969 | 1.14639 | -2.6225 | cytochrome P450 (CYP6AA5) [Source:VB Community Annotation;Acc:AAEL012492] | | | | | | | |  |  |  |  |  |
| AAEL000200 | 464.005 | 75.3489 | -2.62248 |  | |  |  |  |  |  |  |  |  |  |  |  |
| AAEL005738 | 10.6321 | 1.73386 | -2.61637 | yellow protein precursor [Source:VB External Description;Acc:AAEL005738] | | | | | | | |  |  |  |  |  |
| AAEL011702 | 9.02607 | 1.47244 | -2.61589 |  | |  |  |  |  |  |  |  |  |  |  |  |
| AAEL004502 | 5.15751 | 0.841518 | -2.61561 |  | |  |  |  |  |  |  |  |  |  |  |  |
| AAEL003428 | 16.0337 | 2.62259 | -2.61205 |  | |  |  |  |  |  |  |  |  |  |  |  |
| AAEL005518 | 10.4694 | 1.71317 | -2.61143 |  | |  |  |  |  |  |  |  |  |  |  |  |
| AAEL015672 | 12.7389 | 2.08705 | -2.6097 |  | |  |  |  |  |  |  |  |  |  |  |  |
| AAEL017183 | 50.5281 | 8.28296 | -2.60887 |  | |  |  |  |  |  |  |  |  |  |  |  |
| AAEL006007 | 3034.17 | 497.407 | -2.6088 |  | |  |  |  |  |  |  |  |  |  |  |  |
| AAEL003600 | 258.21 | 42.3602 | -2.60776 |  | |  |  |  |  |  |  |  |  |  |  |  |
| AAEL011981 | 21.2701 | 3.4955 | -2.60525 | glutamate decarboxylase [Source:VB External Description;Acc:AAEL011981] | | | | | | | |  |  |  |  |  |
| AAEL001920 | 8.81105 | 1.44803 | -2.60522 |  | |  |  |  |  |  |  |  |  |  |  |  |
| AAEL015114 | 14.7368 | 2.42545 | -2.6031 |  | |  |  |  |  |  |  |  |  |  |  |  |
| AAEL000732 | 44.3398 | 7.30719 | -2.60121 |  | |  |  |  |  |  |  |  |  |  |  |  |
| AAEL006714 | 16.9949 | 2.80146 | -2.60085 |  | |  |  |  |  |  |  |  |  |  |  |  |
| AAEL001031 | 5.82996 | 0.962081 | -2.59926 |  | |  |  |  |  |  |  |  |  |  |  |  |
| AAEL010764 | 16.2423 | 2.68084 | -2.599 | aldehyde dehydrogenase [Source:VB External Description;Acc:AAEL010764] | | | | | | | |  |  |  |  |  |
| AAEL017001 | 2.02663 | 0.335009 | -2.59681 |  | |  |  |  |  |  |  |  |  |  |  |  |
| AAEL010917 | 1.33211 | 0.220367 | -2.59573 | organic anion transporter [Source:VB External Description;Acc:AAEL010917] | | | | | | | |  |  |  |  |  |
| AAEL011452 | 19.3483 | 3.20132 | -2.59547 |  | |  |  |  |  |  |  |  |  |  |  |  |
| AAEL012110 | 27.7993 | 4.60228 | -2.59463 | protease m1 zinc metalloprotease [Source:VB External Description;Acc:AAEL012110] | | | | | | | |  |  |  |  |  |
| AAEL006483 | 493.155 | 81.8084 | -2.59172 |  | |  |  |  |  |  |  |  |  |  |  |  |
| AAEL000428 | 55.389 | 9.19873 | -2.59009 | tryptophan 2,3-dioxygenase (TDO)(EC 1.13.11.11) [Source:VB Community Annotation;Acc:AAEL000428] | | | | | | | | | |  |  |  |
| AAEL002301 | 42.5036 | 7.0627 | -2.58929 | serine protease [Source:VB External Description;Acc:AAEL002301] | | | | | | |  |  |  |  |  |  |
| AAEL006685 | 267.564 | 44.4666 | -2.58909 | G-protein, gamma-subunit, putative [Source:VB External Description;Acc:AAEL006685] | | | | | | | | |  |  |  |  |
| AAEL013867 | 25.8333 | 4.29415 | -2.58879 | beta-mannosidase [Source:VB External Description;Acc:AAEL013867] | | | | | | |  |  |  |  |  |  |
| AAEL010850 | 363.681 | 60.4703 | -2.58837 | troponin i [Source:VB External Description;Acc:AAEL010850] | | | | | | |  |  |  |  |  |  |
| AAEL007394 | 158.021 | 26.3263 | -2.58555 |  | |  |  |  |  |  |  |  |  |  |  |  |
| AAEL011569 | 4.8044 | 0.801375 | -2.58381 |  | |  |  |  |  |  |  |  |  |  |  |  |
| AAEL006562 | 3.26402 | 0.544829 | -2.58277 | acid phosphatase [Source:VB External Description;Acc:AAEL006562] | | | | | | |  |  |  |  |  |  |
| AAEL007784 | 27.1363 | 4.53726 | -2.58033 |  | |  |  |  |  |  |  |  |  |  |  |  |
| AAEL012092 | 43.5303 | 7.27977 | -2.58006 | leucine-rich repeat [Source:VB External Description;Acc:AAEL012092] | | | | | | |  |  |  |  |  |  |
| AAEL005147 | 14.6431 | 2.45054 | -2.57905 |  | |  |  |  |  |  |  |  |  |  |  |  |
| AAEL004824 | 11.1376 | 1.8649 | -2.57826 |  | |  |  |  |  |  |  |  |  |  |  |  |
| AAEL011325 | 16.1685 | 2.70844 | -2.57765 | gonadotropin-releasing hormone receptor [Source:VB External Description;Acc:AAEL011325] | | | | | | | | |  |  |  |  |
| AAEL011573 | 34.8678 | 5.84985 | -2.57543 |  | |  |  |  |  |  |  |  |  |  |  |  |
| AAEL001813 | 1.74852 | 0.29419 | -2.57132 | sodium/solute symporter [Source:VB External Description;Acc:AAEL001813] | | | | | | | |  |  |  |  |  |
| AAEL003186 | 80.1598 | 13.502 | -2.5697 |  | |  |  |  |  |  |  |  |  |  |  |  |
| AAEL003197 | 80.1598 | 13.502 | -2.5697 |  | |  |  |  |  |  |  |  |  |  |  |  |
| AAEL007107 | 2.35853 | 0.397597 | -2.56851 | serine protease, putative [Source:VB External Description;Acc:AAEL007107] | | | | | | | |  |  |  |  |  |
| AAEL003589 | 12.6112 | 2.12617 | -2.56837 | transcription factor, putative [Source:VB External Description;Acc:AAEL003589] | | | | | | | |  |  |  |  |  |
| AAEL018680 | 996.584 | 168.143 | -2.5673 |  | |  |  |  |  |  |  |  |  |  |  |  |
| AAEL018681 | 996.584 | 168.143 | -2.5673 |  | |  |  |  |  |  |  |  |  |  |  |  |
| AAEL017376 | 54.2969 | 9.17369 | -2.5653 |  | |  |  |  |  |  |  |  |  |  |  |  |
| AAEL002665 | 18.1929 | 3.07658 | -2.56398 | matrix metalloproteinase [Source:VB External Description;Acc:AAEL002665] | | | | | | | |  |  |  |  |  |
| AAEL003772 | 1.25023 | 0.211571 | -2.56298 |  | |  |  |  |  |  |  |  |  |  |  |  |
| AAEL002568 | 8.18665 | 1.38753 | -2.56076 |  | |  |  |  |  |  |  |  |  |  |  |  |
| AAEL007109 | 3.08944 | 0.52489 | -2.55726 |  | |  |  |  |  |  |  |  |  |  |  |  |
| AAEL000618 | 4.10351 | 0.697347 | -2.55691 | wingless protein, putative [Source:VB External Description;Acc:AAEL000618] | | | | | | | |  |  |  |  |  |
| AAEL000704 | 29.9421 | 5.09119 | -2.5561 | synaptotagmin, [Source:VB External Description;Acc:AAEL000704] | | | | | | |  |  |  |  |  |  |
| AAEL002545 | 17.181 | 2.92194 | -2.55581 |  | |  |  |  |  |  |  |  |  |  |  |  |
| AAEL003551 | 32.4655 | 5.52853 | -2.55394 |  | |  |  |  |  |  |  |  |  |  |  |  |
| AAEL010324 | 5.6116 | 0.955778 | -2.55366 |  | |  |  |  |  |  |  |  |  |  |  |  |
| AAEL012979 | 2.74172 | 0.467306 | -2.55264 |  | |  |  |  |  |  |  |  |  |  |  |  |
| AAEL003184 | 5.2685 | 0.898057 | -2.55251 | mfs transporter [Source:VB External Description;Acc:AAEL003184] | | | | | | |  |  |  |  |  |  |
| AAEL003037 | 4.80915 | 0.820015 | -2.55206 |  | |  |  |  |  |  |  |  |  |  |  |  |
| AAEL004027 | 121.255 | 20.6954 | -2.55067 | glucose dehydrogenase [Source:VB External Description;Acc:AAEL004027] | | | | | | | |  |  |  |  |  |
| AAEL017444 | 15.1364 | 2.58538 | -2.54957 |  | |  |  |  |  |  |  |  |  |  |  |  |
| AAEL008619 | 146.901 | 25.1185 | -2.54803 |  | |  |  |  |  |  |  |  |  |  |  |  |
| AAEL000474 | 6.89952 | 1.18121 | -2.54623 |  | |  |  |  |  |  |  |  |  |  |  |  |
| AAEL008828 | 14.4109 | 2.47306 | -2.54279 | carbonic anhydrase [Source:VB External Description;Acc:AAEL008828] | | | | | | |  |  |  |  |  |  |
| AAEL012571 | 5.99144 | 1.02889 | -2.54182 |  | |  |  |  |  |  |  |  |  |  |  |  |
| AAEL003345 | 110.046 | 18.9007 | -2.5416 | argininosuccinate lyase [Source:VB External Description;Acc:AAEL003345] | | | | | | | |  |  |  |  |  |
| AAEL003082 | 3.55246 | 0.610552 | -2.54063 | kinectin, putative [Source:VB External Description;Acc:AAEL003082] | | | | | | |  |  |  |  |  |  |
| AAEL013572 | 5.34869 | 0.919776 | -2.53983 | N-acetylgalactosaminyltransferase I, putative [Source:VB External Description;Acc:AAEL013572] | | | | | | | | |  |  |  |  |
| AAEL000838 | 16.83 | 2.89442 | -2.53969 |  | |  |  |  |  |  |  |  |  |  |  |  |
| AAEL008452 | 15.8288 | 2.72356 | -2.53899 | alpha-amylase [Source:VB External Description;Acc:AAEL008452] | | | | | | |  |  |  |  |  |  |
| AAEL012849 | 45.6172 | 7.85472 | -2.53794 |  | |  |  |  |  |  |  |  |  |  |  |  |
| AAEL006200 | 35.8773 | 6.1803 | -2.53732 |  | |  |  |  |  |  |  |  |  |  |  |  |
| AAEL010596 | 8.87125 | 1.52822 | -2.53729 | n-acetylgalactosaminyltransferase [Source:VB External Description;Acc:AAEL010596] | | | | | | | | |  |  |  |  |
| AAEL008609 | 8.14654 | 1.40374 | -2.53691 | zinc carboxypeptidase [Source:VB External Description;Acc:AAEL008609] | | | | | | | |  |  |  |  |  |
| AAEL006695 | 5.257 | 0.905911 | -2.5368 |  | |  |  |  |  |  |  |  |  |  |  |  |
| AAEL002506 | 16.2716 | 2.80668 | -2.53542 | glutamate receptor, ionotropic kainate 1, 2, 3 (glur5, glur6, glur7) [Source:VB External Description;Acc:AAEL002506] | | | | | | | | | | |  |  |
| AAEL000153 | 0.98006 | 0.169304 | -2.53326 |  | |  |  |  |  |  |  |  |  |  |  |  |
| AAEL004676 | 23.6282 | 4.08202 | -2.53316 | (s)-2-hydroxy-acid oxidase [Source:VB External Description;Acc:AAEL004676] | | | | | | | |  |  |  |  |  |
| AAEL001087 | 10.3339 | 1.78845 | -2.53061 | synaptic vesicle protein [Source:VB External Description;Acc:AAEL001087] | | | | | | | |  |  |  |  |  |
| AAEL008587 | 31.7383 | 5.49314 | -2.53052 | glutamate receptor, ionotropic, N-methyl d-aspartate [Source:VB External Description;Acc:AAEL008587] | | | | | | | | | |  |  |  |
| AAEL014619 | 3.98818 | 0.69046 | -2.5301 | cytochrome P450 (CYP9J22) [Source:VB Community Annotation;Acc:AAEL014619] | | | | | | | |  |  |  |  |  |
| AAEL000778 | 1.97951 | 0.342813 | -2.52965 |  | |  |  |  |  |  |  |  |  |  |  |  |
| AAEL003028 | 1.52224 | 0.263743 | -2.52899 | histamine-gated chloride channel subunit [Source:VB External Description;Acc:AAEL003028] | | | | | | | | |  |  |  |  |
| AAEL006451 | 108.09 | 18.7524 | -2.52708 |  | |  |  |  |  |  |  |  |  |  |  |  |
| AAEL002560 | 11.9153 | 2.06763 | -2.52676 |  | |  |  |  |  |  |  |  |  |  |  |  |
| AAEL003837 | 9.84226 | 1.71012 | -2.52489 | ryanodine receptor 3, brain [Source:VB External Description;Acc:AAEL003837] | | | | | | | |  |  |  |  |  |
| AAEL006027 | 14.8501 | 2.58236 | -2.52371 | lipase [Source:VB External Description;Acc:AAEL006027] | | | | | |  |  |  |  |  |  |  |
| AAEL001747 | 5.17743 | 0.901056 | -2.52255 |  | |  |  |  |  |  |  |  |  |  |  |  |
| AAEL009615 | 156.229 | 27.1984 | -2.52206 | ultraviolet wavelength sensitive opsin [Source:VB Community Annotation;Acc:AAEL009615] | | | | | | | | |  |  |  |  |
| AAEL013515 | 197.556 | 34.3954 | -2.52198 | pupal cuticle protein, putative [Source:VB External Description;Acc:AAEL013515] | | | | | | | |  |  |  |  |  |
| AAEL008275 | 10.4639 | 1.8223 | -2.52159 |  | |  |  |  |  |  |  |  |  |  |  |  |
| AAEL001964 | 490.885 | 85.6456 | -2.51893 | protein serine/threonine kinase, putative [Source:VB External Description;Acc:AAEL001964] | | | | | | | | |  |  |  |  |
| AAEL007050 | 8.00553 | 1.39907 | -2.51653 | sugar transporter [Source:VB External Description;Acc:AAEL007050] | | | | | | |  |  |  |  |  |  |
| AAEL007127 | 1.91832 | 0.335348 | -2.51611 |  | |  |  |  |  |  |  |  |  |  |  |  |
| AAEL004616 | 25.2589 | 4.41643 | -2.51584 | actin [Source:VB External Description;Acc:AAEL004616] | | | | | |  |  |  |  |  |  |  |
| AAEL007759 | 3.67693 | 0.643037 | -2.51553 |  | |  |  |  |  |  |  |  |  |  |  |  |
| AAEL002990 | 21.7371 | 3.80287 | -2.515 |  | |  |  |  |  |  |  |  |  |  |  |  |
| AAEL012689 | 4.49279 | 0.787446 | -2.51236 | glucose-methanol-choline (gmc) oxidoreductase [Source:VB External Description;Acc:AAEL012689] | | | | | | | | | |  |  |  |
| AAEL007281 | 8.88048 | 1.55691 | -2.51195 | stretchin-mlck [Source:VB External Description;Acc:AAEL007281] | | | | | | |  |  |  |  |  |  |
| AAEL004022 | 4.75443 | 0.834438 | -2.5104 |  | |  |  |  |  |  |  |  |  |  |  |  |
| AAEL013343 | 7.44885 | 1.30864 | -2.50895 | lethal(2)essential for life protein, l2efl [Source:VB External Description;Acc:AAEL013343] | | | | | | | | |  |  |  |  |
| AAEL002224 | 8.62652 | 1.51613 | -2.50838 |  | |  |  |  |  |  |  |  |  |  |  |  |
| AAEL011369 | 23.7266 | 4.17015 | -2.50834 | endothelin-converting enzyme [Source:VB External Description;Acc:AAEL011369] | | | | | | | |  |  |  |  |  |
| AAEL006719 | 157.157 | 27.6299 | -2.5079 | Alpha-amylase I Precursor (EC 3.2.1.1)(1,4-alpha-D-glucan glucanohydrolase) [Source:VB Community Annotation;Acc:AAEL006719] | | | | | | | | | | | |  |
| AAEL005964 | 3.37785 | 0.593983 | -2.50761 | actin [Source:VB External Description;Acc:AAEL005964] | | | | | |  |  |  |  |  |  |  |
| AAEL017302 | 206.395 | 36.3456 | -2.50555 |  | |  |  |  |  |  |  |  |  |  |  |  |
| AAEL003782 | 5.23709 | 0.922771 | -2.50472 |  | |  |  |  |  |  |  |  |  |  |  |  |
| AAEL009541 | 11.7902 | 2.07772 | -2.50451 |  | |  |  |  |  |  |  |  |  |  |  |  |
| AAEL005117 | 10.0296 | 1.76757 | -2.50442 |  | |  |  |  |  |  |  |  |  |  |  |  |
| AAEL008007 | 14.5362 | 2.56332 | -2.50357 |  | |  |  |  |  |  |  |  |  |  |  |  |
| AAEL000762 | 3.59593 | 0.63464 | -2.50235 | leucine-rich immune protein (Coil-less) [Source:VB Community Annotation;Acc:AAEL000762] | | | | | | | | |  |  |  |  |
| AAEL001203 | 2.2084 | 0.390368 | -2.5001 |  | |  |  |  |  |  |  |  |  |  |  |  |
| AAEL012630 | 41.433 | 7.34405 | -2.49613 |  | |  |  |  |  |  |  |  |  |  |  |  |
| AAEL003030 | 2.82884 | 0.501833 | -2.49493 |  | |  |  |  |  |  |  |  |  |  |  |  |
| AAEL010567 | 11.0907 | 1.96786 | -2.49465 |  | |  |  |  |  |  |  |  |  |  |  |  |
| AAEL001146 | 5.57333 | 0.989347 | -2.49399 | n-acetylgalactosaminyltransferase [Source:VB External Description;Acc:AAEL001146] | | | | | | | | |  |  |  |  |
| AAEL010102 | 17.3977 | 3.08877 | -2.49379 | tetraspanin, putative [Source:VB External Description;Acc:AAEL010102] | | | | | | |  |  |  |  |  |  |
| AAEL009531 | 3.26012 | 0.57881 | -2.49376 | niemann-pick C1 [Source:VB External Description;Acc:AAEL009531] | | | | | | |  |  |  |  |  |  |
| AAEL002580 | 11.6972 | 2.07679 | -2.49373 | predicted protein [Source:VB External Description;Acc:AAEL002580] | | | | | | |  |  |  |  |  |  |
| AAEL002276 | 7.07033 | 1.25552 | -2.49349 | serine protease, putative [Source:VB External Description;Acc:AAEL002276] | | | | | | | |  |  |  |  |  |
| AAEL013123 | 4.39518 | 0.781257 | -2.49205 | GTP-binding protein alpha subunit, gna [Source:VB External Description;Acc:AAEL013123] | | | | | | | | |  |  |  |  |
| AAEL012905 | 3.83772 | 0.682282 | -2.49181 |  | |  |  |  |  |  |  |  |  |  |  |  |
| AAEL011184 | 114.536 | 20.3753 | -2.49091 | mitochondrial phosphate carrier protein [Source:VB External Description;Acc:AAEL011184] | | | | | | | | |  |  |  |  |
| AAEL004582 | 1.34429 | 0.239147 | -2.49088 | beta-galactosidase [Source:VB External Description;Acc:AAEL004582] | | | | | | |  |  |  |  |  |  |
| AAEL003076 | 43.1772 | 7.68552 | -2.49006 | glucosyl/glucuronosyl transferases [Source:VB External Description;Acc:AAEL003076] | | | | | | | | |  |  |  |  |
| AAEL009585 | 2.12265 | 0.378158 | -2.4888 |  | |  |  |  |  |  |  |  |  |  |  |  |
| AAEL010957 | 3.85334 | 0.686602 | -2.48856 | serine collagenase 1 precursor, putative [Source:VB External Description;Acc:AAEL010957] | | | | | | | | |  |  |  |  |
| AAEL006682 | 13.8886 | 2.47694 | -2.48728 |  | |  |  |  |  |  |  |  |  |  |  |  |
| AAEL006526 | 24.183 | 4.31683 | -2.48595 | neurotransmitter gated ion channel [Source:VB External Description;Acc:AAEL006526] | | | | | | | | |  |  |  |  |
| AAEL007193 | 10.1068 | 1.80438 | -2.48575 | amino acid transporter [Source:VB External Description;Acc:AAEL007193] | | | | | | | |  |  |  |  |  |
| AAEL000533 | 198.921 | 35.5161 | -2.48565 | C-Type Lectin (CTL16) [Source:VB Community Annotation;Acc:AAEL000533] | | | | | | | |  |  |  |  |  |
| AAEL010799 | 63.0132 | 11.2722 | -2.48289 |  | |  |  |  |  |  |  |  |  |  |  |  |
| AAEL010783 | 40.9797 | 7.33216 | -2.4826 | sodium/potassium-dependent ATPase beta-2 subunit [Source:VB External Description;Acc:AAEL010783] | | | | | | | | | |  |  |  |
| AAEL001613 | 1.54595 | 0.276694 | -2.48213 | organic anion transporter [Source:VB External Description;Acc:AAEL001613] | | | | | | | |  |  |  |  |  |
| AAEL018000 | 20.0402 | 3.58772 | -2.48176 |  | |  |  |  |  |  |  |  |  |  |  |  |
| AAEL010326 | 7.06941 | 1.26567 | -2.48169 | Phosphotriesterase-related protein (EC 3.1.-.-)(Parathion hydrolase-related protein) [Source:VB External Description;Acc:AAEL010326] | | | | | | | | | | | | |
| AAEL017074 | 87.1245 | 15.6024 | -2.48131 |  | |  |  |  |  |  |  |  |  |  |  |  |
| AAEL003821 | 194.017 | 34.7673 | -2.48038 |  | |  |  |  |  |  |  |  |  |  |  |  |
| AAEL008438 | 9.12283 | 1.6379 | -2.47764 |  | |  |  |  |  |  |  |  |  |  |  |  |
| AAEL003122 | 23.0747 | 4.1532 | -2.47402 |  | |  |  |  |  |  |  |  |  |  |  |  |
| AAEL000309 | 7.46327 | 1.34367 | -2.47363 |  | |  |  |  |  |  |  |  |  |  |  |  |
| AAEL005753 | 14.6665 | 2.64076 | -2.4735 | serine protease [Source:VB External Description;Acc:AAEL005753] | | | | | | |  |  |  |  |  |  |
| AAEL003873 | 49.9778 | 8.99925 | -2.47341 | glycerol-3-phosphate dehydrogenase [Source:VB External Description;Acc:AAEL003873] | | | | | | | | |  |  |  |  |
| AAEL005148 | 1.23742 | 0.222967 | -2.47243 |  | |  |  |  |  |  |  |  |  |  |  |  |
| AAEL005913 | 2.57757 | 0.464507 | -2.47224 |  | |  |  |  |  |  |  |  |  |  |  |  |
| AAEL001261 | 30.9681 | 5.58145 | -2.47207 |  | |  |  |  |  |  |  |  |  |  |  |  |
| AAEL008546 | 25.1165 | 4.53102 | -2.47073 |  | |  |  |  |  |  |  |  |  |  |  |  |
| AAEL017417 | 25.1165 | 4.53102 | -2.47073 |  | |  |  |  |  |  |  |  |  |  |  |  |
| AAEL008246 | 2.60812 | 0.470559 | -2.47057 | phospholipase c epsilon [Source:VB External Description;Acc:AAEL008246] | | | | | | | |  |  |  |  |  |
| AAEL013039 | 38.7144 | 6.99589 | -2.46829 |  | |  |  |  |  |  |  |  |  |  |  |  |
| AAEL007231 | 2.01383 | 0.364209 | -2.4671 | leucine-rich immune protein (Coil-less) [Source:VB Community Annotation;Acc:AAEL007231] | | | | | | | | |  |  |  |  |
| AAEL011677 | 2.681 | 0.484983 | -2.46676 | AMP dependent coa ligase [Source:VB External Description;Acc:AAEL011677] | | | | | | | |  |  |  |  |  |
| AAEL001485 | 7.09322 | 1.2835 | -2.46636 |  | |  |  |  |  |  |  |  |  |  |  |  |
| AAEL006535 | 5.73494 | 1.03779 | -2.46626 |  | |  |  |  |  |  |  |  |  |  |  |  |
| AAEL006216 | 11.9089 | 2.15532 | -2.46606 |  | |  |  |  |  |  |  |  |  |  |  |  |
| AAEL003636 | 6.62157 | 1.19948 | -2.46476 |  | |  |  |  |  |  |  |  |  |  |  |  |
| AAEL006288 | 22.2664 | 4.03942 | -2.46265 |  | |  |  |  |  |  |  |  |  |  |  |  |
| AAEL002704 | 59.6596 | 10.8277 | -2.46203 | Serine Protease Inhibitor (serpin) homologue [Source:VB Community Annotation;Acc:AAEL002704] | | | | | | | | |  |  |  |  |
| AAEL007102 | 7.04036 | 1.2785 | -2.4612 | trypsin, putative [Source:VB External Description;Acc:AAEL007102] | | | | | | |  |  |  |  |  |  |
| AAEL013326 | 12.6447 | 2.30368 | -2.45652 |  | |  |  |  |  |  |  |  |  |  |  |  |
| AAEL008304 | 113.01 | 20.5977 | -2.4559 |  | |  |  |  |  |  |  |  |  |  |  |  |
| AAEL008303 | 113.01 | 20.5977 | -2.4559 |  | |  |  |  |  |  |  |  |  |  |  |  |
| AAEL012311 | 19.9763 | 3.64164 | -2.45563 | vitellogenin, putative [Source:VB External Description;Acc:AAEL012311] | | | | | | |  |  |  |  |  |  |
| AAEL017554 | 26.3706 | 4.81098 | -2.45453 |  | |  |  |  |  |  |  |  |  |  |  |  |
| AAEL011638 | 2.35106 | 0.429493 | -2.4526 | cyclic-nucleotide-gated cation channel [Source:VB External Description;Acc:AAEL011638] | | | | | | | | |  |  |  |  |
| AAEL014152 | 3.78596 | 0.691632 | -2.45258 |  | |  |  |  |  |  |  |  |  |  |  |  |
| AAEL013797 | 1.16123 | 0.2123 | -2.45148 |  | |  |  |  |  |  |  |  |  |  |  |  |
| AAEL011421 | 19.5697 | 3.57825 | -2.45129 | multiple inositol polyphosphate phosphatase [Source:VB External Description;Acc:AAEL011421] | | | | | | | | |  |  |  |  |
| AAEL010851 | 1.09937 | 0.201122 | -2.45054 | synaptotagmin-12 [Source:VB External Description;Acc:AAEL010851] | | | | | | |  |  |  |  |  |  |
| AAEL011332 | 2.40482 | 0.440016 | -2.4503 | dopamine beta hydroxylase [Source:VB External Description;Acc:AAEL011332] | | | | | | | |  |  |  |  |  |
| AAEL004901 | 18.029 | 3.30528 | -2.44748 |  | |  |  |  |  |  |  |  |  |  |  |  |
| AAEL001139 | 2.20192 | 0.403719 | -2.44734 | br serine/threonine-protein kinase [Source:VB External Description;Acc:AAEL001139] | | | | | | | | |  |  |  |  |
| AAEL013645 | 22.4731 | 4.12062 | -2.44727 |  | |  |  |  |  |  |  |  |  |  |  |  |
| AAEL011296 | 745.145 | 136.648 | -2.44705 |  | |  |  |  |  |  |  |  |  |  |  |  |
| AAEL011298 | 745.145 | 136.648 | -2.44705 |  | |  |  |  |  |  |  |  |  |  |  |  |
| AAEL012554 | 0.970176 | 0.177943 | -2.44683 | maltose phosphorylase [Source:VB External Description;Acc:AAEL012554] | | | | | | | |  |  |  |  |  |
| AAEL017087 | 208.26 | 38.2227 | -2.44588 |  | |  |  |  |  |  |  |  |  |  |  |  |
| AAEL002865 | 208.26 | 38.2227 | -2.44588 |  | |  |  |  |  |  |  |  |  |  |  |  |
| AAEL000447 | 2.31557 | 0.425488 | -2.44418 | V-1 protein, putative [Source:VB External Description;Acc:AAEL000447] | | | | | | |  |  |  |  |  |  |
| AAEL018687 | 1074.42 | 197.627 | -2.44271 | NADH dehydrogenase subunit 1 [Source:VB Community Annotation;Acc:AAEL018687] | | | | | | | |  |  |  |  |  |
| AAEL005008 | 83.5475 | 15.3706 | -2.44243 | aquaporin [Source:VB External Description;Acc:AAEL005008] | | | | | | |  |  |  |  |  |  |
| AAEL006069 | 8.24275 | 1.51712 | -2.44179 |  | |  |  |  |  |  |  |  |  |  |  |  |
| AAEL009910 | 30.9709 | 5.70371 | -2.44094 |  | |  |  |  |  |  |  |  |  |  |  |  |
| AAEL009240 | 2.99834 | 0.552352 | -2.4405 | neurotactin [Source:VB External Description;Acc:AAEL009240] | | | | | | |  |  |  |  |  |  |
| AAEL007059 | 17.7604 | 3.27294 | -2.44001 |  | |  |  |  |  |  |  |  |  |  |  |  |
| AAEL006601 | 6.63772 | 1.2233 | -2.43991 | 50-kda dystrophin-associated glycoprotein, putative [Source:VB External Description;Acc:AAEL006601] | | | | | | | | | |  |  |  |
| AAEL002283 | 76.7029 | 14.1398 | -2.43952 |  | |  |  |  |  |  |  |  |  |  |  |  |
| AAEL003748 | 16.3031 | 3.0081 | -2.43822 | cytochrome P450 (CYP9AE1) [Source:VB Community Annotation;Acc:AAEL003748] | | | | | | | |  |  |  |  |  |
| AAEL003886 | 5.07775 | 0.937226 | -2.43772 |  | |  |  |  |  |  |  |  |  |  |  |  |
| AAEL002720 | 43.2909 | 7.99102 | -2.43761 | Serine Protease Inhibitor (serpin) likely cleavage at V/V. [Source:VB Community Annotation;Acc:AAEL002720] | | | | | | | | | | |  |  |
| AAEL001953 | 1.29988 | 0.239947 | -2.4376 |  | |  |  |  |  |  |  |  |  |  |  |  |
| AAEL002228 | 17.368 | 3.20679 | -2.43723 | fatty acid synthase [Source:VB External Description;Acc:AAEL002228] | | | | | | |  |  |  |  |  |  |
| AAEL005496 | 17.4552 | 3.22294 | -2.43721 | zinc/iron transporter [Source:VB External Description;Acc:AAEL005496] | | | | | | |  |  |  |  |  |  |
| AAEL003313 | 37.787 | 6.97972 | -2.43665 | alkaline phosphatase [Source:VB External Description;Acc:AAEL003313] | | | | | | |  |  |  |  |  |  |
| AAEL000937 | 7.49707 | 1.3849 | -2.43654 |  | |  |  |  |  |  |  |  |  |  |  |  |
| AAEL008305 | 208.928 | 38.62 | -2.43558 |  | |  |  |  |  |  |  |  |  |  |  |  |
| AAEL007872 | 6.65312 | 1.23065 | -2.43461 |  | |  |  |  |  |  |  |  |  |  |  |  |
| AAEL002848 | 20.0219 | 3.70716 | -2.43319 | tubulin beta chain [Source:VB External Description;Acc:AAEL002848] | | | | | | |  |  |  |  |  |  |
| AAEL018264 | 2.52946 | 0.468482 | -2.43277 |  | |  |  |  |  |  |  |  |  |  |  |  |
| AAEL004335 | 4.56905 | 0.847099 | -2.43129 | secreted ferritin G subunit precursor, putative [Source:VB External Description;Acc:AAEL004335] | | | | | | | | |  |  |  |  |
| AAEL003150 | 2.69085 | 0.498915 | -2.4312 | alpha-n-acetylglucosaminidase [Source:VB External Description;Acc:AAEL003150] | | | | | | | |  |  |  |  |  |
| AAEL000024 | 14.6598 | 2.71904 | -2.43069 | dopachrome-conversion enzyme (DCE), putative [Source:VB External Description;Acc:AAEL000024] | | | | | | | | | |  |  |  |
| AAEL006252 | 3.65991 | 0.680801 | -2.4265 |  | |  |  |  |  |  |  |  |  |  |  |  |
| AAEL009425 | 10.5174 | 1.95829 | -2.42512 |  | |  |  |  |  |  |  |  |  |  |  |  |
| AAEL017212 | 294.736 | 54.8873 | -2.42488 |  | |  |  |  |  |  |  |  |  |  |  |  |
| AAEL011086 | 4.05422 | 0.755802 | -2.42334 |  | |  |  |  |  |  |  |  |  |  |  |  |
| AAEL009850 | 13.6717 | 2.54997 | -2.42263 | galectin [Source:VB Community Annotation;Acc:AAEL009850] | | | | | | |  |  |  |  |  |  |
| AAEL009847 | 8.22829 | 1.5365 | -2.42095 | microtubule-associated protein [Source:VB External Description;Acc:AAEL009847] | | | | | | | |  |  |  |  |  |
| AAEL012157 | 2.25889 | 0.421855 | -2.42079 |  | |  |  |  |  |  |  |  |  |  |  |  |
| AAEL007926 | 19.7283 | 3.69209 | -2.41776 | retinoid-inducible serine carboxypeptidase (serine carboxypeptidase [Source:VB External Description;Acc:AAEL007926] | | | | | | | | | | |  |  |
| AAEL004391 | 2.16574 | 0.405785 | -2.41607 |  | |  |  |  |  |  |  |  |  |  |  |  |
| AAEL008032 | 5.84409 | 1.09508 | -2.41595 |  | |  |  |  |  |  |  |  |  |  |  |  |
| AAEL009757 | 4.27349 | 0.801225 | -2.41514 |  | |  |  |  |  |  |  |  |  |  |  |  |
| AAEL015331 | 6.72541 | 1.26175 | -2.4142 |  | |  |  |  |  |  |  |  |  |  |  |  |
| AAEL010120 | 3.90298 | 0.73258 | -2.41352 | chondroitin 4-sulfotransferase [Source:VB External Description;Acc:AAEL010120] | | | | | | | |  |  |  |  |  |
| AAEL006617 | 26.3657 | 4.94892 | -2.41348 |  | |  |  |  |  |  |  |  |  |  |  |  |
| AAEL005593 | 128.4 | 24.1082 | -2.41304 |  | |  |  |  |  |  |  |  |  |  |  |  |
| AAEL017454 | 39.8847 | 7.49057 | -2.41269 |  | |  |  |  |  |  |  |  |  |  |  |  |
| AAEL008488 | 6.48544 | 1.21942 | -2.41101 | cysteine-rich secretory protein-2, putative [Source:VB External Description;Acc:AAEL008488] | | | | | | | | |  |  |  |  |
| AAEL010260 | 28.1764 | 5.2979 | -2.411 |  | |  |  |  |  |  |  |  |  |  |  |  |
| AAEL009065 | 3.56413 | 0.670208 | -2.41087 |  | |  |  |  |  |  |  |  |  |  |  |  |
| AAEL008262 | 5.01604 | 0.944142 | -2.40947 | ppa (fragment) [Source:VB External Description;Acc:AAEL008262] | | | | | | |  |  |  |  |  |  |
| AAEL010147 | 11.0412 | 2.07854 | -2.40926 |  | |  |  |  |  |  |  |  |  |  |  |  |
| AAEL017078 | 69.7209 | 13.1306 | -2.40866 |  | |  |  |  |  |  |  |  |  |  |  |  |
| AAEL007104 | 1.74758 | 0.32914 | -2.40858 | voltage-gated potassium channel [Source:VB External Description;Acc:AAEL007104] | | | | | | | |  |  |  |  |  |
| AAEL007064 | 213.827 | 40.2989 | -2.40763 | Gram-Negative Binding Protein (GNBP) or Beta-1 3-Glucan Binding Protein (BGBP). [Source:VB Community Annotation;Acc:AAEL007064] | | | | | | | | | | | | |
| AAEL001586 | 9.13883 | 1.72409 | -2.40618 | glucosyl/glucuronosyl transferases [Source:VB External Description;Acc:AAEL001586] | | | | | | | | |  |  |  |  |
| AAEL008588 | 22.4049 | 4.22803 | -2.40575 | innexin [Source:VB Community Annotation;Acc:AAEL008588] | | | | | |  |  |  |  |  |  |  |
| AAEL001474 | 13.9197 | 2.63327 | -2.4022 |  | |  |  |  |  |  |  |  |  |  |  |  |
| AAEL006351 | 7.68693 | 1.45489 | -2.4015 |  | |  |  |  |  |  |  |  |  |  |  |  |
| AAEL006609 | 31.8952 | 6.03849 | -2.40108 | zinc finger protein [Source:VB External Description;Acc:AAEL006609] | | | | | | |  |  |  |  |  |  |
| AAEL013007 | 47.0626 | 8.91418 | -2.40041 | cytochrome c oxidase, subunit VB, putative [Source:VB External Description;Acc:AAEL013007] | | | | | | | | |  |  |  |  |
| AAEL000612 | 79.314 | 15.0329 | -2.39945 |  | |  |  |  |  |  |  |  |  |  |  |  |
| AAEL012704 | 21.0912 | 3.99915 | -2.39888 |  | |  |  |  |  |  |  |  |  |  |  |  |
| AAEL006644 | 6.90817 | 1.31057 | -2.39811 |  | |  |  |  |  |  |  |  |  |  |  |  |
| AAEL011929 | 35.2256 | 6.68286 | -2.39809 | serine-type enodpeptidase, [Source:VB External Description;Acc:AAEL011929] | | | | | | | |  |  |  |  |  |
| AAEL017108 | 16.884 | 3.20554 | -2.39701 |  | |  |  |  |  |  |  |  |  |  |  |  |
| AAEL007106 | 1.47237 | 0.279748 | -2.39595 | serine protease, putative [Source:VB External Description;Acc:AAEL007106] | | | | | | | |  |  |  |  |  |
| AAEL003123 | 75.471 | 14.3408 | -2.3958 | deoxyribonuclease I, putative [Source:VB External Description;Acc:AAEL003123] | | | | | | | |  |  |  |  |  |
| AAEL005819 | 14.2099 | 2.70036 | -2.39567 | l-allo-threonine aldolase [Source:VB External Description;Acc:AAEL005819] | | | | | | | |  |  |  |  |  |
| AAEL009266 | 10.8385 | 2.05972 | -2.39565 | c4b-binding protein beta chain [Source:VB External Description;Acc:AAEL009266] | | | | | | | |  |  |  |  |  |
| AAEL011460 | 3.78328 | 0.719093 | -2.39539 |  | |  |  |  |  |  |  |  |  |  |  |  |
| AAEL017418 | 2.89208 | 0.549799 | -2.39513 |  | |  |  |  |  |  |  |  |  |  |  |  |
| AAEL006333 | 26.9424 | 5.12225 | -2.39503 | salivary apyrase, putative [Source:VB External Description;Acc:AAEL006333] | | | | | | | |  |  |  |  |  |
| AAEL006051 | 6.52657 | 1.24173 | -2.39398 | beta-sarcoglycan [Source:VB External Description;Acc:AAEL006051] | | | | | | |  |  |  |  |  |  |
| AAEL009679 | 12.1829 | 2.3187 | -2.39347 | secretory Phospholipase A2, putative [Source:VB External Description;Acc:AAEL009679] | | | | | | | | |  |  |  |  |
| AAEL005694 | 4.83569 | 0.921331 | -2.39193 |  | |  |  |  |  |  |  |  |  |  |  |  |
| AAEL014741 | 0.975861 | 0.186028 | -2.39115 | serine/threonine-protein kinase ripk4 [Source:VB External Description;Acc:AAEL014741] | | | | | | | | |  |  |  |  |
| AAEL005993 | 3.90503 | 0.74461 | -2.39078 | semaphorin [Source:VB External Description;Acc:AAEL005993] | | | | | | |  |  |  |  |  |  |
| AAEL002254 | 12.6933 | 2.42077 | -2.39053 |  | |  |  |  |  |  |  |  |  |  |  |  |
| AAEL008085 | 219.239 | 41.8498 | -2.38921 | trypsin [Source:VB External Description;Acc:AAEL008085] | | | | | |  |  |  |  |  |  |  |
| AAEL018696 | 11.8565 | 2.26331 | -2.38917 |  | |  |  |  |  |  |  |  |  |  |  |  |
| AAEL004220 | 22.3428 | 4.26586 | -2.3889 |  | |  |  |  |  |  |  |  |  |  |  |  |
| AAEL007293 | 44.1856 | 8.4445 | -2.38749 | cAMP-dependent protein kinase catalytic subunit [Source:VB External Description;Acc:AAEL007293] | | | | | | | | | |  |  |  |
| AAEL013140 | 4.04152 | 0.772451 | -2.38739 |  | |  |  |  |  |  |  |  |  |  |  |  |
| AAEL012672 | 4.70743 | 0.900189 | -2.38664 | ubiquitin conjugating enzyme E2, putative [Source:VB External Description;Acc:AAEL012672] | | | | | | | | |  |  |  |  |
| AAEL007780 | 90.7875 | 17.3639 | -2.3864 |  | |  |  |  |  |  |  |  |  |  |  |  |
| AAEL003339 | 5.2718 | 1.00837 | -2.38626 |  | |  |  |  |  |  |  |  |  |  |  |  |
| AAEL016998 | 2.2319 | 0.427042 | -2.38582 |  | |  |  |  |  |  |  |  |  |  |  |  |
| AAEL009840 | 45.8528 | 8.77738 | -2.38515 |  | |  |  |  |  |  |  |  |  |  |  |  |
| AAEL012578 | 18.8979 | 3.62098 | -2.38377 | phosphoserine aminotransferase [Source:VB External Description;Acc:AAEL012578] | | | | | | | |  |  |  |  |  |
| AAEL003512 | 46.4232 | 8.8963 | -2.38357 | aquaporin (AQP1(Ae.a [Source:VB Community Annotation;Acc:AAEL003512] | | | | | | | |  |  |  |  |  |
| AAEL001837 | 66.8107 | 12.811 | -2.3827 | lipase [Source:VB External Description;Acc:AAEL001837] | | | | | |  |  |  |  |  |  |  |
| AAEL013013 | 62.0331 | 11.9019 | -2.38184 |  | |  |  |  |  |  |  |  |  |  |  |  |
| AAEL009629 | 50.9242 | 9.7739 | -2.38135 | endoU protein, putative [Source:VB External Description;Acc:AAEL009629] | | | | | | | |  |  |  |  |  |
| AAEL004961 | 5.65972 | 1.08674 | -2.38073 |  | |  |  |  |  |  |  |  |  |  |  |  |
| AAEL002204 | 0.597937 | 0.114935 | -2.37917 | fatty acid synthase [Source:VB External Description;Acc:AAEL002204] | | | | | | |  |  |  |  |  |  |
| AAEL006408 | 145.485 | 27.9773 | -2.37854 |  | |  |  |  |  |  |  |  |  |  |  |  |
| AAEL011676 | 4.31999 | 0.830907 | -2.37827 | AMP dependent coa ligase [Source:VB External Description;Acc:AAEL011676] | | | | | | | |  |  |  |  |  |
| AAEL013570 | 64.3861 | 12.385 | -2.37816 |  | |  |  |  |  |  |  |  |  |  |  |  |
| AAEL001565 | 5.65728 | 1.08958 | -2.37634 | peptidyl-glycine alpha-amidating monooxygenase [Source:VB External Description;Acc:AAEL001565] | | | | | | | | | |  |  |  |
| AAEL003268 | 3.05639 | 0.588679 | -2.37628 |  | |  |  |  |  |  |  |  |  |  |  |  |
| AAEL009300 | 7.14702 | 1.37662 | -2.37621 |  | |  |  |  |  |  |  |  |  |  |  |  |
| AAEL009923 | 2.0039 | 0.386052 | -2.37595 | vesicular inhibitory amino acid transporter, putative [Source:VB External Description;Acc:AAEL009923] | | | | | | | | | |  |  |  |
| AAEL002721 | 13.5705 | 2.61492 | -2.37563 |  | |  |  |  |  |  |  |  |  |  |  |  |
| AAEL004793 | 4.02324 | 0.776029 | -2.37418 | dipeptidyl-peptidase [Source:VB External Description;Acc:AAEL004793] | | | | | | |  |  |  |  |  |  |
| AAEL000854 | 33.6017 | 6.49678 | -2.37074 | fumarylacetoacetate hydrolase [Source:VB External Description;Acc:AAEL000854] | | | | | | | |  |  |  |  |  |
| AAEL010131 | 0.86348 | 0.166953 | -2.37072 | scabrous protein [Source:VB External Description;Acc:AAEL010131] | | | | | | |  |  |  |  |  |  |
| AAEL000669 | 54.4026 | 10.5231 | -2.37011 |  | |  |  |  |  |  |  |  |  |  |  |  |
| AAEL011756 | 24.4338 | 4.72914 | -2.36923 | aldehyde dehydrogenase [Source:VB External Description;Acc:AAEL011756] | | | | | | | |  |  |  |  |  |
| AAEL005509 | 34.68 | 6.71486 | -2.36867 |  | |  |  |  |  |  |  |  |  |  |  |  |
| AAEL009459 | 84.5756 | 16.3773 | -2.36854 |  | |  |  |  |  |  |  |  |  |  |  |  |
| AAEL009330 | 167.07 | 32.356 | -2.36835 | carbonic anhydrase II, putative [Source:VB External Description;Acc:AAEL009330] | | | | | | | |  |  |  |  |  |
| AAEL014422 | 4.04311 | 0.783509 | -2.36744 | syntrophin [Source:VB External Description;Acc:AAEL014422] | | | | | | |  |  |  |  |  |  |
| AAEL014450 | 25.0064 | 4.84804 | -2.36682 | peptidyl-glycine alpha-amidating monooxygenase [Source:VB External Description;Acc:AAEL014450] | | | | | | | | | |  |  |  |
| AAEL012846 | 13.8859 | 2.69259 | -2.36655 |  | |  |  |  |  |  |  |  |  |  |  |  |
| AAEL014457 | 2.02581 | 0.39288 | -2.36634 | neuronal cell adhesion molecule [Source:VB External Description;Acc:AAEL014457] | | | | | | | |  |  |  |  |  |
| AAEL000530 | 5.08815 | 0.987193 | -2.36574 |  | |  |  |  |  |  |  |  |  |  |  |  |
| AAEL012783 | 46.9416 | 9.10873 | -2.36554 | protease m1 zinc metalloprotease [Source:VB External Description;Acc:AAEL012783] | | | | | | | |  |  |  |  |  |
| AAEL011234 | 7.17447 | 1.39324 | -2.36443 | reticulon/nogo receptor [Source:VB External Description;Acc:AAEL011234] | | | | | | | |  |  |  |  |  |
| AAEL002131 | 2.5886 | 0.502962 | -2.36365 |  | |  |  |  |  |  |  |  |  |  |  |  |
| AAEL000940 | 43.5105 | 8.4612 | -2.36243 |  | |  |  |  |  |  |  |  |  |  |  |  |
| AAEL014965 | 27.4345 | 5.3374 | -2.36178 | nova [Source:VB External Description;Acc:AAEL014965] | | | | | |  |  |  |  |  |  |  |
| AAEL014609 | 95.201 | 18.5223 | -2.36171 | cytochrome P450 [Source:VB Community Annotation;Acc:AAEL014609] | | | | | | |  |  |  |  |  |  |
| AAEL008031 | 7.02315 | 1.36798 | -2.36008 | carbonic anhydrase [Source:VB External Description;Acc:AAEL008031] | | | | | | |  |  |  |  |  |  |
| AAEL004919 | 3.33266 | 0.649573 | -2.35911 |  | |  |  |  |  |  |  |  |  |  |  |  |
| AAEL010386 | 5.12461 | 0.99904 | -2.35883 | glucosyl/glucuronosyl transferases [Source:VB External Description;Acc:AAEL010386] | | | | | | | | |  |  |  |  |
| AAEL012850 | 3.38719 | 0.661487 | -2.3563 |  | |  |  |  |  |  |  |  |  |  |  |  |
| AAEL009127 | 21.6701 | 4.23685 | -2.35464 | cytochrome P450 [Source:VB Community Annotation;Acc:AAEL009127] | | | | | | |  |  |  |  |  |  |
| AAEL013703 | 339.618 | 66.5097 | -2.35227 | trypsin [Source:VB External Description;Acc:AAEL013703] | | | | | |  |  |  |  |  |  |  |
| AAEL003764 | 62.01 | 12.1497 | -2.35158 |  | |  |  |  |  |  |  |  |  |  |  |  |
| AAEL008083 | 211.419 | 41.4249 | -2.35154 | 40S ribosomal protein SA [Source:VB Community Annotation;Acc:AAEL008083] | | | | | | | |  |  |  |  |  |
| AAEL009175 | 4.49259 | 0.880825 | -2.35062 | slit protein [Source:VB External Description;Acc:AAEL009175] | | | | | | |  |  |  |  |  |  |
| AAEL013233 | 13.2768 | 2.60327 | -2.35052 | PIWI [Source:VB Community Annotation;Acc:AAEL013233] | | | | | |  |  |  |  |  |  |  |
| AAEL010160 | 6.11471 | 1.19918 | -2.35024 |  | |  |  |  |  |  |  |  |  |  |  |  |
| AAEL007534 | 10.5312 | 2.06567 | -2.34999 |  | |  |  |  |  |  |  |  |  |  |  |  |
| AAEL015483 | 5.47348 | 1.07372 | -2.34984 |  | |  |  |  |  |  |  |  |  |  |  |  |
| AAEL010284 | 8.13532 | 1.5962 | -2.34956 | aliphatic nitrilase, putative [Source:VB External Description;Acc:AAEL010284] | | | | | | | |  |  |  |  |  |
| AAEL010168 | 2798.81 | 549.701 | -2.34809 | 40S ribosomal protein S2 [Source:VB Community Annotation;Acc:AAEL010168] | | | | | | | |  |  |  |  |  |
| AAEL010262 | 5.86826 | 1.15294 | -2.34761 |  | |  |  |  |  |  |  |  |  |  |  |  |
| AAEL017506 | 62.1165 | 12.2166 | -2.34613 |  | |  |  |  |  |  |  |  |  |  |  |  |
| AAEL004750 | 5.52512 | 1.08665 | -2.34612 | nonmuscle myosin heavy chain-A, putative [Source:VB External Description;Acc:AAEL004750] | | | | | | | | |  |  |  |  |
| AAEL008164 | 11.5203 | 2.26705 | -2.34528 |  | |  |  |  |  |  |  |  |  |  |  |  |
| AAEL002680 | 6.53976 | 1.28771 | -2.34443 | AMP dependent ligase [Source:VB External Description;Acc:AAEL002680] | | | | | | | |  |  |  |  |  |
| AAEL004095 | 3.14684 | 0.620011 | -2.34354 | aldo-keto reductase [Source:VB External Description;Acc:AAEL004095] | | | | | | |  |  |  |  |  |  |
| AAEL012518 | 16.4887 | 3.25156 | -2.34228 |  | |  |  |  |  |  |  |  |  |  |  |  |
| AAEL004488 | 2.64186 | 0.521464 | -2.34091 |  | |  |  |  |  |  |  |  |  |  |  |  |
| AAEL011193 | 8.47768 | 1.67382 | -2.34052 | steroid dehydrogenase [Source:VB External Description;Acc:AAEL011193] | | | | | | | |  |  |  |  |  |
| AAEL001230 | 1.27416 | 0.25162 | -2.34023 | myelin transcription factor 1, myt1 [Source:VB External Description;Acc:AAEL001230] | | | | | | | | |  |  |  |  |
| AAEL002669 | 7.09131 | 1.40117 | -2.33942 | AMP dependent ligase [Source:VB External Description;Acc:AAEL002669] | | | | | | | |  |  |  |  |  |
| AAEL001340 | 1.12959 | 0.223599 | -2.33682 |  | |  |  |  |  |  |  |  |  |  |  |  |
| AAEL007233 | 0.82444 | 0.163231 | -2.33649 | zinc finger protein [Source:VB External Description;Acc:AAEL007233] | | | | | | |  |  |  |  |  |  |
| AAEL002511 | 3.58416 | 0.709829 | -2.33609 | ionotropic glutamate receptor subunit ia [Source:VB External Description;Acc:AAEL002511] | | | | | | | | |  |  |  |  |
| AAEL004077 | 3.74416 | 0.741609 | -2.33591 |  | |  |  |  |  |  |  |  |  |  |  |  |
| AAEL018667 | 45798 | 9078.48 | -2.33476 |  | |  |  |  |  |  |  |  |  |  |  |  |
| AAEL018664 | 45798 | 9078.48 | -2.33476 |  | |  |  |  |  |  |  |  |  |  |  |  |
| AAEL018671 | 45798 | 9078.48 | -2.33476 |  | |  |  |  |  |  |  |  |  |  |  |  |
| AAEL018669 | 45798 | 9078.48 | -2.33476 |  | |  |  |  |  |  |  |  |  |  |  |  |
| AAEL018668 | 45798 | 9078.48 | -2.33476 |  | |  |  |  |  |  |  |  |  |  |  |  |
| AAEL018662 | 45798 | 9078.48 | -2.33476 |  | |  |  |  |  |  |  |  |  |  |  |  |
| AAEL008141 | 32.8174 | 6.51443 | -2.33275 | period circadian protein [Source:VB Community Annotation;Acc:AAEL008141] | | | | | | | |  |  |  |  |  |
| AAEL010570 | 3.00389 | 0.596328 | -2.33266 | acetylcholine receptor, beta-type subunit invertebrate [Source:VB External Description;Acc:AAEL010570] | | | | | | | | | |  |  |  |
| AAEL003530 | 975.504 | 193.706 | -2.33228 | acidic ribosomal protein P1, putative [Source:VB Community Annotation;Acc:AAEL003530] | | | | | | | | |  |  |  |  |
| AAEL005332 | 306.789 | 60.9215 | -2.33222 |  | |  |  |  |  |  |  |  |  |  |  |  |
| AAEL009670 | 521.361 | 103.711 | -2.32972 | C-Type Lysozyme (Lys-D). [Source:VB Community Annotation;Acc:AAEL009670] | | | | | | | |  |  |  |  |  |
| AAEL010946 | 2.87599 | 0.572953 | -2.32757 | cytochrome P450 (CYP314A1) [Source:VB Community Annotation;Acc:AAEL010946] | | | | | | | |  |  |  |  |  |
| AAEL015018 | 1.78523 | 0.355738 | -2.32722 | toll [Source:VB External Description;Acc:AAEL015018] | | | | | |  |  |  |  |  |  |  |
| AAEL005317 | 51.4269 | 10.2551 | -2.32619 | titin (connectin) [Source:VB External Description;Acc:AAEL005317] | | | | | | |  |  |  |  |  |  |
| AAEL010856 | 5.68011 | 1.13364 | -2.32496 | dopamine beta hydroxylase [Source:VB External Description;Acc:AAEL010856] | | | | | | | |  |  |  |  |  |
| AAEL013864 | 4.73019 | 0.944293 | -2.32459 |  | |  |  |  |  |  |  |  |  |  |  |  |
| AAEL012513 | 20.8273 | 4.15789 | -2.32455 | calcium-binding protein E63-1 [Source:VB External Description;Acc:AAEL012513] | | | | | | | |  |  |  |  |  |
| AAEL010821 | 2487.61 | 496.813 | -2.32399 | 60S acidic ribosomal protein P0 [Source:VB Community Annotation;Acc:AAEL010821] | | | | | | | |  |  |  |  |  |
| AAEL003586 | 6.25132 | 1.24905 | -2.32334 | neuronal cell adhesion molecule [Source:VB External Description;Acc:AAEL003586] | | | | | | | |  |  |  |  |  |
| AAEL008079 | 56.1347 | 11.2233 | -2.3224 | trypsin-alpha, putative [Source:VB External Description;Acc:AAEL008079] | | | | | | | |  |  |  |  |  |
| AAEL003619 | 14.4118 | 2.88402 | -2.3211 | sodium/chloride dependent amino acid transporter [Source:VB External Description;Acc:AAEL003619] | | | | | | | | | |  |  |  |
| AAEL014315 | 18.5581 | 3.71381 | -2.32108 |  | |  |  |  |  |  |  |  |  |  |  |  |
| AAEL003003 | 5.95403 | 1.19305 | -2.31921 | glutamate-gated chloride channel [Source:VB External Description;Acc:AAEL003003] | | | | | | | |  |  |  |  |  |
| AAEL006305 | 7.60383 | 1.52446 | -2.31843 |  | |  |  |  |  |  |  |  |  |  |  |  |
| AAEL014523 | 12.1231 | 2.43095 | -2.31817 | neuroendocrine convertase [Source:VB External Description;Acc:AAEL014523] | | | | | | | |  |  |  |  |  |
| AAEL001799 | 5.05446 | 1.01426 | -2.31714 |  | |  |  |  |  |  |  |  |  |  |  |  |
| AAEL011506 | 4.19055 | 0.840959 | -2.31703 |  | |  |  |  |  |  |  |  |  |  |  |  |
| AAEL001409 | 63.6601 | 12.7763 | -2.31692 |  | |  |  |  |  |  |  |  |  |  |  |  |
| AAEL001434 | 63.6601 | 12.7763 | -2.31692 |  | |  |  |  |  |  |  |  |  |  |  |  |
| AAEL006448 | 1.59627 | 0.320447 | -2.31655 |  | |  |  |  |  |  |  |  |  |  |  |  |
| AAEL006984 | 11.5957 | 2.32797 | -2.31645 | cytochrome P450 [Source:VB Community Annotation;Acc:AAEL006984] | | | | | | |  |  |  |  |  |  |
| AAEL013491 | 17.5535 | 3.52551 | -2.31585 | short-chain dehydrogenase [Source:VB External Description;Acc:AAEL013491] | | | | | | | |  |  |  |  |  |
| AAEL005567 | 112.077 | 22.5189 | -2.31528 | nucleosome assembly protein [Source:VB External Description;Acc:AAEL005567] | | | | | | | |  |  |  |  |  |
| AAEL002288 | 11.4371 | 2.29812 | -2.31519 | serine protease [Source:VB External Description;Acc:AAEL002288] | | | | | | |  |  |  |  |  |  |
| AAEL005218 | 33.1305 | 6.6613 | -2.31428 |  | |  |  |  |  |  |  |  |  |  |  |  |
| AAEL010313 | 6.22596 | 1.25224 | -2.31378 |  | |  |  |  |  |  |  |  |  |  |  |  |
| AAEL017023 | 19.2678 | 3.88006 | -2.31204 |  | |  |  |  |  |  |  |  |  |  |  |  |
| AAEL001149 | 5.3441 | 1.0767 | -2.31134 |  | |  |  |  |  |  |  |  |  |  |  |  |
| AAEL006995 | 6.71529 | 1.35325 | -2.31102 | sodium/solute symporter [Source:VB External Description;Acc:AAEL006995] | | | | | | | |  |  |  |  |  |
| AAEL010864 | 5.53089 | 1.1152 | -2.31021 | TRP-phosphatase, putative [Source:VB External Description;Acc:AAEL010864] | | | | | | | |  |  |  |  |  |
| AAEL012763 | 19.3992 | 3.91291 | -2.30968 | leucine-rich immune protein (Coil-less) [Source:VB Community Annotation;Acc:AAEL012763] | | | | | | | | |  |  |  |  |
| AAEL007420 | 12.671 | 2.55794 | -2.30848 | Serine Protease Inhibitor (serpin) homologue - unlikely to be inhibitory [Source:VB Community Annotation;Acc:AAEL007420] | | | | | | | | | | | |  |
| AAEL006888 | 0.988264 | 0.19976 | -2.30663 |  | |  |  |  |  |  |  |  |  |  |  |  |
| AAEL007016 | 3.47924 | 0.703305 | -2.30655 | sodium/solute symporter [Source:VB External Description;Acc:AAEL007016] | | | | | | | |  |  |  |  |  |
| AAEL012450 | 2.48207 | 0.502028 | -2.30571 | serine/threonine-protein kinase [Source:VB External Description;Acc:AAEL012450] | | | | | | | |  |  |  |  |  |
| AAEL014594 | 2.91412 | 0.590046 | -2.30416 | cytochrome P450 [Source:VB Community Annotation;Acc:AAEL014594] | | | | | | |  |  |  |  |  |  |
| AAEL006485 | 168.373 | 34.0924 | -2.30414 | inosine-uridine preferring nucleoside hydrolase [Source:VB External Description;Acc:AAEL006485] | | | | | | | | | |  |  |  |
| AAEL001438 | 1.21896 | 0.246931 | -2.30347 |  | |  |  |  |  |  |  |  |  |  |  |  |
| AAEL001851 | 55.206 | 11.1847 | -2.3033 |  | |  |  |  |  |  |  |  |  |  |  |  |
| AAEL010653 | 3.74486 | 0.758835 | -2.30305 | alpha-l-fucosidase [Source:VB External Description;Acc:AAEL010653] | | | | | | |  |  |  |  |  |  |
| AAEL008635 | 15.3946 | 3.11987 | -2.30286 | ABC transporter [Source:VB External Description;Acc:AAEL008635] | | | | | | |  |  |  |  |  |  |
| AAEL017100 | 7.23968 | 1.46861 | -2.30148 |  | |  |  |  |  |  |  |  |  |  |  |  |
| AAEL012998 | 7.23968 | 1.46861 | -2.30148 |  | |  |  |  |  |  |  |  |  |  |  |  |
| AAEL012380 | 16.5686 | 3.36172 | -2.30118 | Peptidoglycan Recognition Protein (Long) [Source:VB Community Annotation;Acc:AAEL012380] | | | | | | | | |  |  |  |  |
| AAEL007024 | 46.5197 | 9.45151 | -2.29923 | cytochrome P450 [Source:VB Community Annotation;Acc:AAEL007024] | | | | | | |  |  |  |  |  |  |
| AAEL012691 | 4.78098 | 0.971451 | -2.29909 | sodium/solute symporter [Source:VB External Description;Acc:AAEL012691] | | | | | | | |  |  |  |  |  |
| AAEL007224 | 7.47765 | 1.51947 | -2.29901 | leucine-rich immune protein (Coil-less) [Source:VB Community Annotation;Acc:AAEL007224] | | | | | | | | |  |  |  |  |
| AAEL002896 | 2.28124 | 0.464187 | -2.29704 |  | |  |  |  |  |  |  |  |  |  |  |  |
| AAEL015445 | 9.60496 | 1.95756 | -2.29472 | cysteine dioxygenase [Source:VB External Description;Acc:AAEL015445] | | | | | | |  |  |  |  |  |  |
| AAEL017228 | 162.699 | 33.1996 | -2.29297 |  | |  |  |  |  |  |  |  |  |  |  |  |
| AAEL018202 | 8.86835 | 1.81188 | -2.29118 |  | |  |  |  |  |  |  |  |  |  |  |  |
| AAEL008383 | 47.8442 | 9.78887 | -2.28913 |  | |  |  |  |  |  |  |  |  |  |  |  |
| AAEL014527 | 8.8063 | 1.80192 | -2.289 | potassium-dependent sodium-calcium exchanger, putative [Source:VB External Description;Acc:AAEL014527] | | | | | | | | | |  |  |  |
| AAEL002555 | 3.41408 | 0.698698 | -2.28876 | sodium/solute symporter [Source:VB External Description;Acc:AAEL002555] | | | | | | | |  |  |  |  |  |
| AAEL017022 | 931.768 | 190.71 | -2.28859 |  | |  |  |  |  |  |  |  |  |  |  |  |
| AAEL001504 | 33.1793 | 6.79216 | -2.28834 | Dual specificity tyrosine-phosphorylation-regulated kinase [Source:VB External Description;Acc:AAEL001504] | | | | | | | | | | |  |  |
| AAEL010257 | 47.2046 | 9.66584 | -2.28796 |  | |  |  |  |  |  |  |  |  |  |  |  |
| AAEL014846 | 13.2469 | 2.71534 | -2.28645 | innexin [Source:VB Community Annotation;Acc:AAEL014846] | | | | | |  |  |  |  |  |  |  |
| AAEL012230 | 3.08978 | 0.633688 | -2.28566 | flagellar protein, putative [Source:VB External Description;Acc:AAEL012230] | | | | | | | |  |  |  |  |  |
| AAEL004246 | 7.55992 | 1.55085 | -2.28531 | still life, sif [Source:VB External Description;Acc:AAEL004246] | | | | | | |  |  |  |  |  |  |
| AAEL003295 | 3.34317 | 0.685828 | -2.2853 |  | |  |  |  |  |  |  |  |  |  |  |  |
| AAEL013628 | 55.8141 | 11.45 | -2.28528 | trypsin-eta, putative [Source:VB External Description;Acc:AAEL013628] | | | | | | |  |  |  |  |  |  |
| AAEL000164 | 4.92901 | 1.01169 | -2.28453 | d-amino acid oxidase [Source:VB External Description;Acc:AAEL000164] | | | | | | |  |  |  |  |  |  |
| AAEL004092 | 1.54181 | 0.316571 | -2.28402 | deoxyribonuclease I, putative [Source:VB External Description;Acc:AAEL004092] | | | | | | | |  |  |  |  |  |
| AAEL000405 | 8.22101 | 1.68813 | -2.28389 | odd Oz protein [Source:VB External Description;Acc:AAEL000405] | | | | | | |  |  |  |  |  |  |
| AAEL000471 | 5.87958 | 1.20746 | -2.28373 | monocarboxylate transporter [Source:VB External Description;Acc:AAEL000471] | | | | | | | |  |  |  |  |  |
| AAEL003118 | 11.7462 | 2.41274 | -2.28345 |  | |  |  |  |  |  |  |  |  |  |  |  |
| AAEL017047 | 34.8844 | 7.16592 | -2.28336 |  | |  |  |  |  |  |  |  |  |  |  |  |
| AAEL007587 | 7.8499 | 1.61468 | -2.28143 |  | |  |  |  |  |  |  |  |  |  |  |  |
| AAEL010144 | 1.12776 | 0.232018 | -2.28115 |  | |  |  |  |  |  |  |  |  |  |  |  |
| AAEL009947 | 4.35776 | 0.896757 | -2.2808 | homeotic antennapedia protein, putative [Source:VB External Description;Acc:AAEL009947] | | | | | | | | |  |  |  |  |
| AAEL009795 | 2.63703 | 0.54294 | -2.28005 | papilin [Source:VB External Description;Acc:AAEL009795] | | | | | |  |  |  |  |  |  |  |
| AAEL002683 | 8.60154 | 1.7724 | -2.27889 | aldehyde oxidase [Source:VB External Description;Acc:AAEL002683] | | | | | | |  |  |  |  |  |  |
| AAEL012764 | 22.0233 | 4.53833 | -2.27879 |  | |  |  |  |  |  |  |  |  |  |  |  |
| AAEL013801 | 2.47194 | 0.509878 | -2.27742 | sulphate transporter [Source:VB External Description;Acc:AAEL013801] | | | | | | |  |  |  |  |  |  |
| AAEL005529 | 2.19422 | 0.452815 | -2.27672 |  | |  |  |  |  |  |  |  |  |  |  |  |
| AAEL017386 | 29.108 | 6.0072 | -2.27665 |  | |  |  |  |  |  |  |  |  |  |  |  |
| AAEL011901 | 4.31528 | 0.890728 | -2.2764 | 1-acyl-sn-glycerol-3-phosphate acyltransferase [Source:VB External Description;Acc:AAEL011901] | | | | | | | | | |  |  |  |
| AAEL010183 | 27.5514 | 5.68982 | -2.27567 |  | |  |  |  |  |  |  |  |  |  |  |  |
| AAEL005052 | 38.4709 | 7.94581 | -2.2755 | tubulin beta chain [Source:VB External Description;Acc:AAEL005052] | | | | | | |  |  |  |  |  |  |
| AAEL005895 | 26.6871 | 5.51241 | -2.27539 |  | |  |  |  |  |  |  |  |  |  |  |  |
| AAEL010180 | 33.2922 | 6.87681 | -2.27537 |  | |  |  |  |  |  |  |  |  |  |  |  |
| AAEL002547 | 5.60392 | 1.15791 | -2.27491 | dipeptidase [Source:VB External Description;Acc:AAEL002547] | | | | | | |  |  |  |  |  |  |
| AAEL015287 | 4.95636 | 1.02472 | -2.27405 | sodium/chloride dependent amino acid transporter [Source:VB External Description;Acc:AAEL015287] | | | | | | | | | |  |  |  |
| AAEL016967 | 12.0272 | 2.48712 | -2.27375 |  | |  |  |  |  |  |  |  |  |  |  |  |
| AAEL004851 | 46960.7 | 9712.4 | -2.27355 |  | |  |  |  |  |  |  |  |  |  |  |  |
| AAEL007450 | 3.98901 | 0.827037 | -2.27001 | odd skipped, putative [Source:VB External Description;Acc:AAEL007450] | | | | | | |  |  |  |  |  |  |
| AAEL001548 | 19.6465 | 4.07347 | -2.26994 | glucosyl/glucuronosyl transferases [Source:VB External Description;Acc:AAEL001548] | | | | | | | | |  |  |  |  |
| AAEL000993 | 15.6378 | 3.24375 | -2.26931 | dally [Source:VB External Description;Acc:AAEL000993] | | | | | |  |  |  |  |  |  |  |
| AAEL013984 | 20.0392 | 4.16467 | -2.26655 |  | |  |  |  |  |  |  |  |  |  |  |  |
| AAEL006342 | 69.0939 | 14.3616 | -2.26634 | epoxide hydrolase [Source:VB External Description;Acc:AAEL006342] | | | | | | |  |  |  |  |  |  |
| AAEL006232 | 2.63754 | 0.548558 | -2.26547 | GPCR Orphan/Putative Class D Family [Source:VB Community Annotation;Acc:AAEL006232] | | | | | | | | |  |  |  |  |
| AAEL017429 | 16.5274 | 3.44007 | -2.26435 |  | |  |  |  |  |  |  |  |  |  |  |  |
| AAEL003977 | 7.99434 | 1.66461 | -2.2638 | elongase, putative [Source:VB External Description;Acc:AAEL003977] | | | | | | |  |  |  |  |  |  |
| AAEL018294 | 1.74427 | 0.363477 | -2.26269 |  | |  |  |  |  |  |  |  |  |  |  |  |
| AAEL006815 | 4.94105 | 1.02982 | -2.26242 | cytochrome P450 [Source:VB Community Annotation;Acc:AAEL006815] | | | | | | |  |  |  |  |  |  |
| AAEL010252 | 0.780335 | 0.162674 | -2.26211 |  | |  |  |  |  |  |  |  |  |  |  |  |
| AAEL012086 | 69.2927 | 14.4593 | -2.2607 | leucine-rich immune protein (Long) [Source:VB Community Annotation;Acc:AAEL012086] | | | | | | | | |  |  |  |  |
| AAEL012135 | 25.462 | 5.32192 | -2.25833 | galectin [Source:VB Community Annotation;Acc:AAEL012135] | | | | | | |  |  |  |  |  |  |
| AAEL015136 | 46.7117 | 9.76851 | -2.25757 | Niemann-Pick Type C-2, putative [Source:VB External Description;Acc:AAEL015136] | | | | | | | |  |  |  |  |  |
| AAEL001754 | 15.7548 | 3.2962 | -2.25692 |  | |  |  |  |  |  |  |  |  |  |  |  |
| AAEL007502 | 5.0671 | 1.06115 | -2.25553 | iroquois-class homeodomain protein irx [Source:VB External Description;Acc:AAEL007502] | | | | | | | | |  |  |  |  |
| AAEL011125 | 1.1961 | 0.250608 | -2.25484 |  | |  |  |  |  |  |  |  |  |  |  |  |
| AAEL001744 | 6.96528 | 1.45968 | -2.25453 | jnk interacting protein (jip) [Source:VB External Description;Acc:AAEL001744] | | | | | | | |  |  |  |  |  |
| AAEL002242 | 2.66248 | 0.558277 | -2.25372 |  | |  |  |  |  |  |  |  |  |  |  |  |
| AAEL005323 | 2.54811 | 0.534326 | -2.25363 | kinesin motor protein, putative [Source:VB External Description;Acc:AAEL005323] | | | | | | | |  |  |  |  |  |
| AAEL013694 | 201.614 | 42.2895 | -2.25323 | 40S ribosomal protein SA [Source:VB Community Annotation;Acc:AAEL013694] | | | | | | | |  |  |  |  |  |
| AAEL002390 | 10.8071 | 2.26721 | -2.253 | zinc finger protein [Source:VB External Description;Acc:AAEL002390] | | | | | | |  |  |  |  |  |  |
| AAEL006260 | 14.0926 | 2.95682 | -2.25282 | serine protease, putative [Source:VB External Description;Acc:AAEL006260] | | | | | | | |  |  |  |  |  |
| AAEL000780 | 4.16919 | 0.874836 | -2.25268 | amino acid transporter [Source:VB External Description;Acc:AAEL000780] | | | | | | | |  |  |  |  |  |
| AAEL017325 | 43.8522 | 9.20261 | -2.25253 | Clip-Domain Serine Protease family B. (Truncated Protease). [Source:VB Community Annotation;Acc:AAEL017325] | | | | | | | | | | |  |  |
| AAEL014436 | 11.5212 | 2.42047 | -2.25092 |  | |  |  |  |  |  |  |  |  |  |  |  |
| AAEL014434 | 11.5212 | 2.42047 | -2.25092 |  | |  |  |  |  |  |  |  |  |  |  |  |
| AAEL017144 | 120.031 | 25.2419 | -2.24951 |  | |  |  |  |  |  |  |  |  |  |  |  |
| AAEL017121 | 44.6251 | 9.39728 | -2.24754 |  | |  |  |  |  |  |  |  |  |  |  |  |
| AAEL005442 | 2.26871 | 0.47791 | -2.24706 | guanylate cyclase beta 1 subunit [Source:VB External Description;Acc:AAEL005442] | | | | | | | |  |  |  |  |  |
| AAEL010523 | 29.2394 | 6.16075 | -2.24674 |  | |  |  |  |  |  |  |  |  |  |  |  |
| AAEL002373 | 1.73375 | 0.365449 | -2.24615 | juvenile hormone-inducible protein, putative [Source:VB External Description;Acc:AAEL002373] | | | | | | | | |  |  |  |  |
| AAEL001323 | 13.7982 | 2.90907 | -2.24585 |  | |  |  |  |  |  |  |  |  |  |  |  |
| AAEL003831 | 15.0559 | 3.17822 | -2.24403 | fatty acid hydroxylase [Source:VB External Description;Acc:AAEL003831] | | | | | | | |  |  |  |  |  |
| AAEL001355 | 15.8569 | 3.35253 | -2.24178 |  | |  |  |  |  |  |  |  |  |  |  |  |
| AAEL001963 | 3.94788 | 0.834718 | -2.24172 | protein serine/threonine kinase, putative [Source:VB External Description;Acc:AAEL001963] | | | | | | | | |  |  |  |  |
| AAEL005237 | 8.68894 | 1.83739 | -2.24152 |  | |  |  |  |  |  |  |  |  |  |  |  |
| AAEL001298 | 1.49432 | 0.316007 | -2.24146 |  | |  |  |  |  |  |  |  |  |  |  |  |
| AAEL007004 | 7.43967 | 1.57359 | -2.24118 | GPCR Bride of Sevenless Family [Source:VB Community Annotation;Acc:AAEL007004] | | | | | | | | |  |  |  |  |
| AAEL001338 | 4.96113 | 1.04995 | -2.24035 | helix-loop-helix transcription factor [Source:VB External Description;Acc:AAEL001338] | | | | | | | | |  |  |  |  |
| AAEL002583 | 3.16022 | 0.669447 | -2.23899 | Toll-like receptor [Source:VB Community Annotation;Acc:AAEL002583] | | | | | | |  |  |  |  |  |  |
| AAEL002518 | 4.33095 | 0.917701 | -2.23859 | glutamate receptor, ionotropic kainate 1, 2, 3 (glur5, glur6, glur7) [Source:VB External Description;Acc:AAEL002518] | | | | | | | | | | |  |  |
| AAEL008963 | 18.6752 | 3.95813 | -2.23823 | tyrosine aminotransferase [Source:VB External Description;Acc:AAEL008963] | | | | | | | |  |  |  |  |  |
| AAEL005255 | 33.9034 | 7.18775 | -2.23782 | PAR-domain protein 1 [Source:VB Community Annotation;Acc:AAEL005255] | | | | | | | |  |  |  |  |  |
| AAEL005667 | 78.8325 | 16.7261 | -2.23669 | LIM domain-binding protein, putative [Source:VB External Description;Acc:AAEL005667] | | | | | | | | |  |  |  |  |
| AAEL005315 | 14.3147 | 3.03739 | -2.23659 |  | |  |  |  |  |  |  |  |  |  |  |  |
| AAEL009461 | 2.51819 | 0.534517 | -2.23608 | histone H1, putative [Source:VB External Description;Acc:AAEL009461] | | | | | | |  |  |  |  |  |  |
| AAEL001176 | 124.653 | 26.4737 | -2.23528 |  | |  |  |  |  |  |  |  |  |  |  |  |
| AAEL017083 | 124.653 | 26.4737 | -2.23528 |  | |  |  |  |  |  |  |  |  |  |  |  |
| AAEL001543 | 1.0435 | 0.221675 | -2.23491 |  | |  |  |  |  |  |  |  |  |  |  |  |
| AAEL001107 | 2282.57 | 485.003 | -2.2346 |  | |  |  |  |  |  |  |  |  |  |  |  |
| AAEL011566 | 3.85797 | 0.820517 | -2.23324 | cell adhesion molecule [Source:VB External Description;Acc:AAEL011566] | | | | | | | |  |  |  |  |  |
| AAEL007703 | 347.159 | 73.8431 | -2.23306 |  | |  |  |  |  |  |  |  |  |  |  |  |
| AAEL013040 | 2.3964 | 0.510113 | -2.23198 |  | |  |  |  |  |  |  |  |  |  |  |  |
| AAEL017513 | 30.5679 | 6.51083 | -2.2311 |  | |  |  |  |  |  |  |  |  |  |  |  |
| AAEL014137 | 4.79316 | 1.02132 | -2.23054 | Clip-Domain Serine Protease family B. [Source:VB Community Annotation;Acc:AAEL014137] | | | | | | | | |  |  |  |  |
| AAEL003766 | 4.90773 | 1.04591 | -2.2303 |  | |  |  |  |  |  |  |  |  |  |  |  |
| AAEL009173 | 7.1864 | 1.53174 | -2.2301 | fasciclin ii (fas ii) [Source:VB External Description;Acc:AAEL009173] | | | | | | |  |  |  |  |  |  |
| AAEL007407 | 87.7243 | 18.7021 | -2.22977 |  | |  |  |  |  |  |  |  |  |  |  |  |
| AAEL007408 | 87.7243 | 18.7021 | -2.22977 |  | |  |  |  |  |  |  |  |  |  |  |  |
| AAEL014853 | 1.92432 | 0.411264 | -2.22621 | otoferlin [Source:VB External Description;Acc:AAEL014853] | | | | | |  |  |  |  |  |  |  |
| AAEL005619 | 5.64534 | 1.20702 | -2.22561 | defective proboscis extension response, putative [Source:VB External Description;Acc:AAEL005619] | | | | | | | | | |  |  |  |
| AAEL018150 | 8.16672 | 1.74703 | -2.22485 |  | |  |  |  |  |  |  |  |  |  |  |  |
| AAEL000254 | 7.69169 | 1.64572 | -2.22458 | nuclear factor i [Source:VB External Description;Acc:AAEL000254] | | | | | | |  |  |  |  |  |  |
| AAEL000595 | 3.92441 | 0.840891 | -2.22248 | beat protein [Source:VB External Description;Acc:AAEL000595] | | | | | | |  |  |  |  |  |  |
| AAEL005179 | 52.7916 | 11.3198 | -2.22145 |  | |  |  |  |  |  |  |  |  |  |  |  |
| AAEL002294 | 12.6362 | 2.7133 | -2.21944 | sulphate transporter [Source:VB External Description;Acc:AAEL002294] | | | | | | |  |  |  |  |  |  |
| AAEL012687 | 13.2806 | 2.85211 | -2.21922 |  | |  |  |  |  |  |  |  |  |  |  |  |
| AAEL012678 | 13.2806 | 2.85211 | -2.21922 |  | |  |  |  |  |  |  |  |  |  |  |  |
| AAEL005282 | 5.98026 | 1.28659 | -2.21665 |  | |  |  |  |  |  |  |  |  |  |  |  |
| AAEL017276 | 22.3697 | 4.81799 | -2.21504 |  | |  |  |  |  |  |  |  |  |  |  |  |
| AAEL005093 | 29.0232 | 6.25211 | -2.21479 | Clip-Domain Serine Protease family B. [Source:VB Community Annotation;Acc:AAEL005093] | | | | | | | | |  |  |  |  |
| AAEL001368 | 3.40488 | 0.733833 | -2.21408 | kek1 [Source:VB External Description;Acc:AAEL001368] | | | | | |  |  |  |  |  |  |  |
| AAEL013333 | 20.5875 | 4.43727 | -2.21402 | testican [Source:VB External Description;Acc:AAEL013333] | | | | | |  |  |  |  |  |  |  |
| AAEL005499 | 2.75556 | 0.593994 | -2.21383 | ATP-binding cassette transporter [Source:VB External Description;Acc:AAEL005499] | | | | | | | | |  |  |  |  |
| AAEL011203 | 1.67156 | 0.360483 | -2.21319 |  | |  |  |  |  |  |  |  |  |  |  |  |
| AAEL007131 | 1.98494 | 0.428094 | -2.2131 | sugar transporter [Source:VB External Description;Acc:AAEL007131] | | | | | | |  |  |  |  |  |  |
| AAEL017328 | 21.1293 | 4.55779 | -2.21284 |  | |  |  |  |  |  |  |  |  |  |  |  |
| AAEL013629 | 25.1914 | 5.43458 | -2.21269 | trypsin-alpha, putative [Source:VB External Description;Acc:AAEL013629] | | | | | | | |  |  |  |  |  |
| AAEL008241 | 5.71809 | 1.23401 | -2.21218 |  | |  |  |  |  |  |  |  |  |  |  |  |
| AAEL018697 | 5.24358 | 1.13162 | -2.21216 | potassium/chloride symporter, putative [Source:VB External Description;Acc:AAEL018697] | | | | | | | | |  |  |  |  |
| AAEL010294 | 5.68628 | 1.22734 | -2.21195 | membrane-associated guanylate kinase (maguk) [Source:VB External Description;Acc:AAEL010294] | | | | | | | | | |  |  |  |
| AAEL006605 | 4.58903 | 0.991762 | -2.21012 | juvenile hormone-inducible protein, putative [Source:VB External Description;Acc:AAEL006605] | | | | | | | | |  |  |  |  |
| AAEL002587 | 12.4075 | 2.68269 | -2.20946 | odorant binding protein OBP11 [Source:VB Community Annotation;Acc:AAEL002587] | | | | | | | |  |  |  |  |  |
| AAEL004383 | 6.74119 | 1.45797 | -2.20905 |  | |  |  |  |  |  |  |  |  |  |  |  |
| AAEL010712 | 10.9647 | 2.37216 | -2.20859 | low-density lipoprotein receptor (ldl) [Source:VB External Description;Acc:AAEL010712] | | | | | | | | |  |  |  |  |
| AAEL014616 | 45.062 | 9.75315 | -2.20797 | cytochrome P450 (CYP9J27) [Source:VB Community Annotation;Acc:AAEL014616] | | | | | | | |  |  |  |  |  |
| AAEL011859 | 8.77915 | 1.90093 | -2.20738 |  | |  |  |  |  |  |  |  |  |  |  |  |
| AAEL012457 | 30.6858 | 6.6486 | -2.20645 | alcohol dehydrogenase [Source:VB External Description;Acc:AAEL012457] | | | | | | | |  |  |  |  |  |
| AAEL000799 | 1.00008 | 0.216839 | -2.20542 |  | |  |  |  |  |  |  |  |  |  |  |  |
| AAEL018183 | 1.74615 | 0.378755 | -2.20484 |  | |  |  |  |  |  |  |  |  |  |  |  |
| AAEL001905 | 5.09526 | 1.10526 | -2.20478 |  | |  |  |  |  |  |  |  |  |  |  |  |
| AAEL014082 | 8.11713 | 1.7623 | -2.20351 | odorant binding protein OBP34 [Source:VB Community Annotation;Acc:AAEL014082] | | | | | | | |  |  |  |  |  |
| AAEL001724 | 4.02084 | 0.87371 | -2.20227 | GPCR Orphan/Putative Class B Family [Source:VB Community Annotation;Acc:AAEL001724] | | | | | | | | |  |  |  |  |
| AAEL014181 | 1.90123 | 0.413338 | -2.20154 |  | |  |  |  |  |  |  |  |  |  |  |  |
| AAEL012249 | 4.46383 | 0.970777 | -2.20107 |  | |  |  |  |  |  |  |  |  |  |  |  |
| AAEL005531 | 15.5748 | 3.38898 | -2.20029 | venom allergen [Source:VB External Description;Acc:AAEL005531] | | | | | | |  |  |  |  |  |  |
| AAEL012528 | 6.1199 | 1.33273 | -2.19912 | tetraspanin, putative [Source:VB External Description;Acc:AAEL012528] | | | | | | |  |  |  |  |  |  |
| AAEL013421 | 5.8566 | 1.27612 | -2.1983 | alpha-amylase [Source:VB External Description;Acc:AAEL013421] | | | | | | |  |  |  |  |  |  |
| AAEL003144 | 32.0787 | 6.99355 | -2.19752 |  | |  |  |  |  |  |  |  |  |  |  |  |
| AAEL009858 | 19.2167 | 4.1895 | -2.19751 | SIFamide [Source:VB Community Annotation;Acc:AAEL009858] | | | | | | |  |  |  |  |  |  |
| AAEL004509 | 7.85399 | 1.71347 | -2.19651 |  | |  |  |  |  |  |  |  |  |  |  |  |
| AAEL018343 | 4.60634 | 1.00532 | -2.19597 |  | |  |  |  |  |  |  |  |  |  |  |  |
| AAEL010513 | 20.2066 | 4.41414 | -2.19462 | class b basic helix-loop-helix protein (bhlhb) (differentially expressed in chondrocytes) (mdec) (sharp) [Source:VB External Description;Acc:AAEL010513] | | | | | | | | | | | | |
| AAEL012932 | 68.278 | 14.9205 | -2.19412 |  | |  |  |  |  |  |  |  |  |  |  |  |
| AAEL017073 | 30.2015 | 6.60981 | -2.19194 |  | |  |  |  |  |  |  |  |  |  |  |  |
| AAEL014607 | 16.9498 | 3.70963 | -2.19192 | cytochrome P450 [Source:VB Community Annotation;Acc:AAEL014607] | | | | | | |  |  |  |  |  |  |
| AAEL011364 | 2.145 | 0.469497 | -2.19179 |  | |  |  |  |  |  |  |  |  |  |  |  |
| AAEL008106 | 142.183 | 31.1307 | -2.19133 |  | |  |  |  |  |  |  |  |  |  |  |  |
| AAEL010169 | 6053.29 | 1326.08 | -2.19055 |  | |  |  |  |  |  |  |  |  |  |  |  |
| AAEL003970 | 37.3848 | 8.19442 | -2.18974 | amino acid transporter [Source:VB External Description;Acc:AAEL003970] | | | | | | | |  |  |  |  |  |
| AAEL007785 | 1.4559 | 0.319331 | -2.18879 | leucine-rich transmembrane protein [Source:VB External Description;Acc:AAEL007785] | | | | | | | | |  |  |  |  |
| AAEL009014 | 2.86866 | 0.629662 | -2.18773 |  | |  |  |  |  |  |  |  |  |  |  |  |
| AAEL002815 | 23.142 | 5.08159 | -2.18716 |  | |  |  |  |  |  |  |  |  |  |  |  |
| AAEL007010 | 31.4514 | 6.91025 | -2.18631 | cytochrome P450 [Source:VB Community Annotation;Acc:AAEL007010] | | | | | | |  |  |  |  |  |  |
| AAEL005243 | 2.53947 | 0.558278 | -2.18547 |  | |  |  |  |  |  |  |  |  |  |  |  |
| AAEL006136 | 38.1752 | 8.39599 | -2.18486 | serine protease, putative [Source:VB External Description;Acc:AAEL006136] | | | | | | | |  |  |  |  |  |
| AAEL012207 | 917.144 | 201.831 | -2.184 | myosin light chain 1, [Source:VB External Description;Acc:AAEL012207] | | | | | | |  |  |  |  |  |  |
| AAEL004230 | 38.7849 | 8.53874 | -2.1834 |  | |  |  |  |  |  |  |  |  |  |  |  |
| AAEL001592 | 17.2173 | 3.79099 | -2.18322 |  | |  |  |  |  |  |  |  |  |  |  |  |
| AAEL017406 | 180.536 | 39.7634 | -2.18277 |  | |  |  |  |  |  |  |  |  |  |  |  |
| AAEL011038 | 2.27657 | 0.501704 | -2.18195 | integrin alpha-ps [Source:VB External Description;Acc:AAEL011038] | | | | | | |  |  |  |  |  |  |
| AAEL017342 | 114.226 | 25.1735 | -2.18191 |  | |  |  |  |  |  |  |  |  |  |  |  |
| AAEL009845 | 15.123 | 3.33494 | -2.18101 | galectin [Source:VB Community Annotation;Acc:AAEL009845] | | | | | | |  |  |  |  |  |  |
| AAEL010771 | 7.1917 | 1.58601 | -2.18093 |  | |  |  |  |  |  |  |  |  |  |  |  |
| AAEL010868 | 4.73208 | 1.04432 | -2.17991 | sugar transporter [Source:VB External Description;Acc:AAEL010868] | | | | | | |  |  |  |  |  |  |
| AAEL009523 | 10.8214 | 2.38845 | -2.17974 |  | |  |  |  |  |  |  |  |  |  |  |  |
| AAEL008153 | 37.2664 | 8.22592 | -2.17963 |  | |  |  |  |  |  |  |  |  |  |  |  |
| AAEL011178 | 4.40319 | 0.973736 | -2.17695 | posterior sex combs protein [Source:VB External Description;Acc:AAEL011178] | | | | | | | |  |  |  |  |  |
| AAEL002692 | 4.83335 | 1.06962 | -2.17593 | beat protein [Source:VB External Description;Acc:AAEL002692] | | | | | | |  |  |  |  |  |  |
| AAEL005336 | 25.1664 | 5.57039 | -2.17565 | d-3-phosphoglycerate dehydrogenase [Source:VB External Description;Acc:AAEL005336] | | | | | | | | |  |  |  |  |
| AAEL001763 | 76.3143 | 16.9083 | -2.17422 |  | |  |  |  |  |  |  |  |  |  |  |  |
| AAEL012778 | 52.8715 | 11.7194 | -2.17359 | protease m1 zinc metalloprotease [Source:VB External Description;Acc:AAEL012778] | | | | | | | |  |  |  |  |  |
| AAEL001794 | 18.315 | 4.06032 | -2.17336 | macroglobulin/complement [Source:VB External Description;Acc:AAEL001794] | | | | | | | |  |  |  |  |  |
| AAEL000161 | 4.0924 | 0.907528 | -2.17293 |  | |  |  |  |  |  |  |  |  |  |  |  |
| AAEL002765 | 4.9521 | 1.09837 | -2.17267 | Sevenup nuclear receptor [Source:VB Community Annotation;Acc:AAEL002765] | | | | | | | |  |  |  |  |  |
| AAEL013612 | 14.5033 | 3.21937 | -2.17153 |  | |  |  |  |  |  |  |  |  |  |  |  |
| AAEL017529 | 102.124 | 22.6819 | -2.17071 |  | |  |  |  |  |  |  |  |  |  |  |  |
| AAEL017035 | 15.3879 | 3.41956 | -2.16992 |  | |  |  |  |  |  |  |  |  |  |  |  |
| AAEL007771 | 7149.42 | 1589.05 | -2.16966 | 60S ribosomal protein L22 [Source:VB Community Annotation;Acc:AAEL007771] | | | | | | | |  |  |  |  |  |
| AAEL012091 | 1.6377 | 0.36418 | -2.16895 |  | |  |  |  |  |  |  |  |  |  |  |  |
| AAEL001306 | 65.7908 | 14.6434 | -2.16763 |  | |  |  |  |  |  |  |  |  |  |  |  |
| AAEL005003 | 9.98403 | 2.22353 | -2.16677 | serine/threonine-protein kinase, putative [Source:VB External Description;Acc:AAEL005003] | | | | | | | | |  |  |  |  |
| AAEL005762 | 4.02904 | 0.897579 | -2.16632 | leucine-rich transmembrane proteins [Source:VB External Description;Acc:AAEL005762] | | | | | | | | |  |  |  |  |
| AAEL015659 | 2.01472 | 0.449061 | -2.1656 |  | |  |  |  |  |  |  |  |  |  |  |  |
| AAEL007493 | 41.7306 | 9.30318 | -2.16531 |  | |  |  |  |  |  |  |  |  |  |  |  |
| AAEL013613 | 101.202 | 22.5691 | -2.16482 | pyruvate dehydrogenase [Source:VB External Description;Acc:AAEL013613] | | | | | | | |  |  |  |  |  |
| AAEL000333 | 10.2692 | 2.29058 | -2.16453 | cysteine-rich venom protein, putative [Source:VB External Description;Acc:AAEL000333] | | | | | | | | |  |  |  |  |
| AAEL018021 | 64.4199 | 14.3694 | -2.16451 |  | |  |  |  |  |  |  |  |  |  |  |  |
| AAEL005692 | 64.4199 | 14.3694 | -2.16451 |  | |  |  |  |  |  |  |  |  |  |  |  |
| AAEL005596 | 49.3019 | 11.0021 | -2.16387 | trypsin-epsilon, putative [Source:VB External Description;Acc:AAEL005596] | | | | | | | |  |  |  |  |  |
| AAEL014847 | 42.7574 | 9.55081 | -2.16248 | innexin [Source:VB Community Annotation;Acc:AAEL014847] | | | | | |  |  |  |  |  |  |  |
| AAEL001429 | 3.77615 | 0.84389 | -2.16179 | amino acid transporter [Source:VB External Description;Acc:AAEL001429] | | | | | | | |  |  |  |  |  |
| AAEL002638 | 14.1913 | 3.17422 | -2.16053 | cytochrome P450 (CYP9J6) [Source:VB Community Annotation;Acc:AAEL002638] | | | | | | | |  |  |  |  |  |
| AAEL012093 | 7.72605 | 1.72816 | -2.1605 | leucine-rich transmembrane protein [Source:VB External Description;Acc:AAEL012093] | | | | | | | | |  |  |  |  |
| AAEL005444 | 21.4288 | 4.79746 | -2.15921 | pyrokinin, putative [Source:VB External Description;Acc:AAEL005444] | | | | | | |  |  |  |  |  |  |
| AAEL006323 | 15.8343 | 3.5459 | -2.15883 |  | |  |  |  |  |  |  |  |  |  |  |  |
| AAEL013588 | 26.9759 | 6.04656 | -2.15748 |  | |  |  |  |  |  |  |  |  |  |  |  |
| AAEL017320 | 40.1874 | 9.01206 | -2.15682 |  | |  |  |  |  |  |  |  |  |  |  |  |
| AAEL000288 | 34.6385 | 7.77263 | -2.1559 |  | |  |  |  |  |  |  |  |  |  |  |  |
| AAEL009094 | 3.02204 | 0.678278 | -2.15558 | WSCD family member AAEL009094 [Source:VB External Description;Acc:AAEL009094] | | | | | | | | |  |  |  |  |
| AAEL017539 | 2.05568 | 0.461557 | -2.15504 | cytochrome P450 [Source:VB Community Annotation;Acc:AAEL017539] | | | | | | |  |  |  |  |  |  |
| AAEL000757 | 43.2832 | 9.7184 | -2.15502 | anterior fat body protein [Source:VB External Description;Acc:AAEL000757] | | | | | | | |  |  |  |  |  |
| AAEL009403 | 7817.97 | 1755.72 | -2.15473 |  | |  |  |  |  |  |  |  |  |  |  |  |
| AAEL006573 | 45.1916 | 10.1559 | -2.15373 |  | |  |  |  |  |  |  |  |  |  |  |  |
| AAEL009831 | 2.66743 | 0.59968 | -2.15319 | pyrroline-5-carboxylate reductase [Source:VB External Description;Acc:AAEL009831] | | | | | | | | |  |  |  |  |
| AAEL000960 | 15.7272 | 3.53781 | -2.15233 |  | |  |  |  |  |  |  |  |  |  |  |  |
| AAEL002227 | 1.35755 | 0.305454 | -2.15198 | fatty acid synthase [Source:VB External Description;Acc:AAEL002227] | | | | | | |  |  |  |  |  |  |
| AAEL011126 | 27.3241 | 6.14942 | -2.15165 | alcohol dehydrogenase [Source:VB External Description;Acc:AAEL011126] | | | | | | | |  |  |  |  |  |
| AAEL002357 | 6.95176 | 1.56508 | -2.15114 |  | |  |  |  |  |  |  |  |  |  |  |  |
| AAEL008430 | 952.68 | 214.594 | -2.15038 |  | |  |  |  |  |  |  |  |  |  |  |  |
| AAEL009818 | 28.0867 | 6.32774 | -2.15013 |  | |  |  |  |  |  |  |  |  |  |  |  |
| AAEL000523 | 6.27043 | 1.41292 | -2.14989 | ring finger protein [Source:VB External Description;Acc:AAEL000523] | | | | | | |  |  |  |  |  |  |
| AAEL014755 | 22.474 | 5.06569 | -2.14943 | tep2 [Source:VB External Description;Acc:AAEL014755] | | | | | |  |  |  |  |  |  |  |
| AAEL001184 | 5.02852 | 1.13389 | -2.14886 |  | |  |  |  |  |  |  |  |  |  |  |  |
| AAEL003603 | 72.8828 | 16.4348 | -2.14882 | transcription factor, putative [Source:VB External Description;Acc:AAEL003603] | | | | | | | |  |  |  |  |  |
| AAEL006604 | 4.86572 | 1.09761 | -2.14829 |  | |  |  |  |  |  |  |  |  |  |  |  |
| AAEL010172 | 8.06151 | 1.81924 | -2.14771 | Leucokinins Precursor [Contains Leucokinin-1;Leucokinin-3;Leucokinin-2] [Source:VB External Description;Acc:AAEL010172] | | | | | | | | | | | |  |
| AAEL004725 | 12.7492 | 2.87835 | -2.14709 |  | |  |  |  |  |  |  |  |  |  |  |  |
| AAEL006366 | 6.66972 | 1.50605 | -2.14686 |  | |  |  |  |  |  |  |  |  |  |  |  |
| AAEL009123 | 9.52772 | 2.15196 | -2.14648 | cytochrome P450 [Source:VB Community Annotation;Acc:AAEL009123] | | | | | | |  |  |  |  |  |  |
| AAEL009492 | 15.8987 | 3.59117 | -2.14638 |  | |  |  |  |  |  |  |  |  |  |  |  |
| AAEL017132 | 151.097 | 34.1356 | -2.14612 | C-Type Lysozyme (Lys-C). [Source:VB Community Annotation;Acc:AAEL017132] | | | | | | | |  |  |  |  |  |
| AAEL011867 | 3.22728 | 0.729637 | -2.14507 |  | |  |  |  |  |  |  |  |  |  |  |  |
| AAEL000504 | 0.999165 | 0.225934 | -2.14482 |  | |  |  |  |  |  |  |  |  |  |  |  |
| AAEL005337 | 26.2114 | 5.9311 | -2.14382 | carbonic anhydrase [Source:VB External Description;Acc:AAEL005337] | | | | | | |  |  |  |  |  |  |
| AAEL012613 | 10.4909 | 2.37428 | -2.14358 |  | |  |  |  |  |  |  |  |  |  |  |  |
| AAEL013618 | 7.32432 | 1.65838 | -2.14292 |  | |  |  |  |  |  |  |  |  |  |  |  |
| AAEL006210 | 91.6986 | 20.7802 | -2.14169 | PFTAIRE-interacting factor 1A, putative [Source:VB External Description;Acc:AAEL006210] | | | | | | | | |  |  |  |  |
| AAEL006704 | 1352.82 | 306.867 | -2.14028 | fibrinogen and fibronectin [Source:VB External Description;Acc:AAEL006704] | | | | | | | |  |  |  |  |  |
| AAEL005645 | 1.81814 | 0.412724 | -2.13921 |  | |  |  |  |  |  |  |  |  |  |  |  |
| AAEL000599 | 0.630898 | 0.143279 | -2.13858 | wingless protein, putative [Source:VB External Description;Acc:AAEL000599] | | | | | | | |  |  |  |  |  |
| AAEL001844 | 21.3229 | 4.84686 | -2.13728 |  | |  |  |  |  |  |  |  |  |  |  |  |
| AAEL001840 | 21.3229 | 4.84686 | -2.13728 |  | |  |  |  |  |  |  |  |  |  |  |  |
| AAEL006283 | 5.49645 | 1.25018 | -2.13636 | GPCR Myosuppressin Family [Source:VB Community Annotation;Acc:AAEL006283] | | | | | | | |  |  |  |  |  |
| AAEL013388 | 2.64508 | 0.601847 | -2.13584 |  | |  |  |  |  |  |  |  |  |  |  |  |
| AAEL010466 | 7.53988 | 1.71616 | -2.13536 | mitogen activated protein kinase kinase kinase 4, mapkkk4, mekk4 [Source:VB External Description;Acc:AAEL010466] | | | | | | | | | | |  |  |
| AAEL018222 | 9.89566 | 2.2539 | -2.13437 |  | |  |  |  |  |  |  |  |  |  |  |  |
| AAEL014140 | 8.47532 | 1.93178 | -2.13333 | Clip-Domain Serine Protease family B. [Source:VB Community Annotation;Acc:AAEL014140] | | | | | | | | |  |  |  |  |
| AAEL008159 | 45.6563 | 10.4175 | -2.1318 | short-chain dehydrogenase [Source:VB External Description;Acc:AAEL008159] | | | | | | | |  |  |  |  |  |
| AAEL003066 | 88.2828 | 20.1441 | -2.13178 | brain chitinase and chia [Source:VB External Description;Acc:AAEL003066] | | | | | | | |  |  |  |  |  |
| AAEL004247 | 9.16425 | 2.09285 | -2.13055 | Sialin, Sodium/sialic acid cotransporter, putative [Source:VB External Description;Acc:AAEL004247] | | | | | | | | | |  |  |  |
| AAEL012532 | 47.6778 | 10.9012 | -2.12883 | tetraspanin 29fb [Source:VB External Description;Acc:AAEL012532] | | | | | | |  |  |  |  |  |  |
| AAEL017089 | 74.2295 | 16.9727 | -2.12878 |  | |  |  |  |  |  |  |  |  |  |  |  |
| AAEL005017 | 46.1173 | 10.5802 | -2.12394 |  | |  |  |  |  |  |  |  |  |  |  |  |
| AAEL002049 | 20.823 | 4.7779 | -2.12373 | circadian protein clock/arnt/bmal/pas [Source:VB External Description;Acc:AAEL002049] | | | | | | | | |  |  |  |  |
| AAEL008826 | 17.7298 | 4.07115 | -2.12267 |  | |  |  |  |  |  |  |  |  |  |  |  |
| AAEL010773 | 11.506 | 2.6432 | -2.12203 | Clip-Domain Serine Protease family E. Protease homologue. [Source:VB Community Annotation;Acc:AAEL010773] | | | | | | | | | | |  |  |
| AAEL000627 | 537.463 | 123.478 | -2.12192 | cecropin anti-microbial peptide [Source:VB Community Annotation;Acc:AAEL000627] | | | | | | | |  |  |  |  |  |
| AAEL000953 | 6.68489 | 1.53839 | -2.11949 |  | |  |  |  |  |  |  |  |  |  |  |  |
| AAEL012103 | 7.01431 | 1.61454 | -2.11918 |  | |  |  |  |  |  |  |  |  |  |  |  |
| AAEL004931 | 18.2173 | 4.20308 | -2.11579 | beta-hexosaminidase b [Source:VB External Description;Acc:AAEL004931] | | | | | | | |  |  |  |  |  |
| AAEL006909 | 49.3961 | 11.4172 | -2.11319 |  | |  |  |  |  |  |  |  |  |  |  |  |
| AAEL004369 | 108.942 | 25.2109 | -2.11145 | alpha-glucosidase [Source:VB External Description;Acc:AAEL004369] | | | | | | |  |  |  |  |  |  |
| AAEL005978 | 9.29272 | 2.15104 | -2.11106 |  | |  |  |  |  |  |  |  |  |  |  |  |
| AAEL003697 | 35.1354 | 8.13897 | -2.11001 | Serine Protease Inhibitor (serpin) homologue - unlikely to be inhibitory. [Source:VB Community Annotation;Acc:AAEL003697] | | | | | | | | | | | |  |
| AAEL003854 | 10.7691 | 2.49545 | -2.10952 |  | |  |  |  |  |  |  |  |  |  |  |  |
| AAEL003860 | 10.7691 | 2.49545 | -2.10952 |  | |  |  |  |  |  |  |  |  |  |  |  |
| AAEL001498 | 1.53798 | 0.356416 | -2.1094 |  | |  |  |  |  |  |  |  |  |  |  |  |
| AAEL006253 | 5.95496 | 1.38017 | -2.10925 |  | |  |  |  |  |  |  |  |  |  |  |  |
| AAEL005451 | 8822.71 | 2045.12 | -2.10903 | 60S ribosomal protein L38 [Source:VB Community Annotation;Acc:AAEL005451] | | | | | | | |  |  |  |  |  |
| AAEL010061 | 6.769 | 1.56908 | -2.10902 |  | |  |  |  |  |  |  |  |  |  |  |  |
| AAEL006339 | 1.47295 | 0.341616 | -2.10827 | peptidylglycine alpha-amidating monooxygenase COOH-terminal interactor protein-1 [Source:VB External Description;Acc:AAEL006339] | | | | | | | | | | | | |
| AAEL004880 | 1.88327 | 0.436885 | -2.10791 |  | |  |  |  |  |  |  |  |  |  |  |  |
| AAEL018123 | 8.91898 | 2.06921 | -2.1078 |  | |  |  |  |  |  |  |  |  |  |  |  |
| AAEL004098 | 10.1585 | 2.35682 | -2.10778 | neuromusculin [Source:VB External Description;Acc:AAEL004098] | | | | | | |  |  |  |  |  |  |
| AAEL014188 | 1893.05 | 439.293 | -2.10745 | serine-type enodpeptidase, [Source:VB External Description;Acc:AAEL014188] | | | | | | | |  |  |  |  |  |
| AAEL007992 | 4.19859 | 0.97475 | -2.1068 | trypsin, putative [Source:VB External Description;Acc:AAEL007992] | | | | | | |  |  |  |  |  |  |
| AAEL008176 | 190.226 | 44.2131 | -2.10517 |  | |  |  |  |  |  |  |  |  |  |  |  |
| AAEL005983 | 3.31639 | 0.771302 | -2.10424 |  | |  |  |  |  |  |  |  |  |  |  |  |
| AAEL008033 | 4.93601 | 1.14807 | -2.10413 |  | |  |  |  |  |  |  |  |  |  |  |  |
| AAEL012774 | 1.39089 | 0.323596 | -2.10374 | protease m1 zinc metalloprotease [Source:VB External Description;Acc:AAEL012774] | | | | | | | |  |  |  |  |  |
| AAEL009601 | 62.8171 | 14.62 | -2.10321 | pyridoxine kinase [Source:VB External Description;Acc:AAEL009601] | | | | | | |  |  |  |  |  |  |
| AAEL004816 | 24.0112 | 5.59246 | -2.10215 |  | |  |  |  |  |  |  |  |  |  |  |  |
| AAEL004809 | 15.4667 | 3.60548 | -2.1009 |  | |  |  |  |  |  |  |  |  |  |  |  |
| AAEL006575 | 44.9131 | 10.4716 | -2.10065 | troponin C [Source:VB External Description;Acc:AAEL006575] | | | | | | |  |  |  |  |  |  |
| AAEL009219 | 66.5688 | 15.5216 | -2.10057 | predicted protein [Source:VB External Description;Acc:AAEL009219] | | | | | | |  |  |  |  |  |  |
| AAEL012712 | 13.9855 | 3.26185 | -2.10017 | Clip-Domain Serine Protease family C. [Source:VB Community Annotation;Acc:AAEL012712] | | | | | | | | |  |  |  |  |
| AAEL000545 | 5.40659 | 1.26352 | -2.09727 |  | |  |  |  |  |  |  |  |  |  |  |  |
| AAEL000334 | 0.820503 | 0.191867 | -2.09641 | villin [Source:VB External Description;Acc:AAEL000334] | | | | | |  |  |  |  |  |  |  |
| AAEL009404 | 7.06639 | 1.65295 | -2.09593 | DNA-binding protein smubp-2 [Source:VB External Description;Acc:AAEL009404] | | | | | | | |  |  |  |  |  |
| AAEL007473 | 27.9788 | 6.54982 | -2.09481 | cytochrome P450 [Source:VB Community Annotation;Acc:AAEL007473] | | | | | | |  |  |  |  |  |  |
| AAEL012398 | 16.2881 | 3.81612 | -2.09364 |  | |  |  |  |  |  |  |  |  |  |  |  |
| AAEL008502 | 18.94 | 4.43769 | -2.09356 |  | |  |  |  |  |  |  |  |  |  |  |  |
| AAEL006000 | 257.465 | 60.3245 | -2.09356 |  | |  |  |  |  |  |  |  |  |  |  |  |
| AAEL006194 | 41.7801 | 9.79365 | -2.0929 |  | |  |  |  |  |  |  |  |  |  |  |  |
| AAEL006467 | 3.7358 | 0.875719 | -2.09288 | alcohol dehydrogenase [Source:VB External Description;Acc:AAEL006467] | | | | | | | |  |  |  |  |  |
| AAEL013611 | 14.1516 | 3.31734 | -2.09287 | AMP dependent ligase [Source:VB External Description;Acc:AAEL013611] | | | | | | | |  |  |  |  |  |
| AAEL010854 | 14.0314 | 3.29218 | -2.09155 | ubiquitin specific protease 2, [Source:VB External Description;Acc:AAEL010854] | | | | | | | |  |  |  |  |  |
| AAEL009821 | 0.67976 | 0.159519 | -2.09129 |  | |  |  |  |  |  |  |  |  |  |  |  |
| AAEL006866 | 588.385 | 138.115 | -2.09089 |  | |  |  |  |  |  |  |  |  |  |  |  |
| AAEL006873 | 588.385 | 138.115 | -2.09089 |  | |  |  |  |  |  |  |  |  |  |  |  |
| AAEL006522 | 6.48857 | 1.52347 | -2.09054 | turtle protein, isoform [Source:VB External Description;Acc:AAEL006522] | | | | | | | |  |  |  |  |  |
| AAEL008907 | 4.6367 | 1.08944 | -2.08951 |  | |  |  |  |  |  |  |  |  |  |  |  |
| AAEL011937 | 3.81329 | 0.896094 | -2.08932 | glucosyl/glucuronosyl transferases [Source:VB External Description;Acc:AAEL011937] | | | | | | | | |  |  |  |  |
| AAEL015124 | 1.36739 | 0.321439 | -2.0888 |  | |  |  |  |  |  |  |  |  |  |  |  |
| AAEL006638 | 2.08748 | 0.491047 | -2.08783 | n-acetyllactosaminide beta-1,3-n-acetylglucosaminyltransferase [Source:VB External Description;Acc:AAEL006638] | | | | | | | | | | |  |  |
| AAEL013812 | 11.7868 | 2.77337 | -2.08746 |  | |  |  |  |  |  |  |  |  |  |  |  |
| AAEL014382 | 16.2848 | 3.83549 | -2.08604 | C-Type Lectin (CTL) - mannose binding. [Source:VB Community Annotation;Acc:AAEL014382] | | | | | | | | |  |  |  |  |
| AAEL017297 | 2.09618 | 0.493727 | -2.08598 | cytochrome P450 [Source:VB Community Annotation;Acc:AAEL017297] | | | | | | |  |  |  |  |  |  |
| AAEL010372 | 13.7521 | 3.23992 | -2.08562 | aldehyde oxidase [Source:VB External Description;Acc:AAEL010372] | | | | | | |  |  |  |  |  |  |
| AAEL001209 | 28.2643 | 6.65969 | -2.08545 | sodium-dependent phosphate transporter [Source:VB External Description;Acc:AAEL001209] | | | | | | | | |  |  |  |  |
| AAEL017598 | 3.30896 | 0.780616 | -2.08369 | 18S_rRNA [Source:RNAmmer;Acc:18S_rRNA] | | | | |  |  |  |  |  |  |  |  |
| AAEL012077 | 3.61889 | 0.854047 | -2.08316 | NAD dehydrogenase [Source:VB External Description;Acc:AAEL012077] | | | | | | |  |  |  |  |  |  |
| AAEL000057 | 3.73056 | 0.880809 | -2.08249 | Toll-like receptor [Source:VB Community Annotation;Acc:AAEL000057] | | | | | | |  |  |  |  |  |  |
| AAEL004481 | 63.9353 | 15.1044 | -2.08165 |  | |  |  |  |  |  |  |  |  |  |  |  |
| AAEL011910 | 1.32539 | 0.313152 | -2.08148 |  | |  |  |  |  |  |  |  |  |  |  |  |
| AAEL003051 | 2.63156 | 0.622551 | -2.07965 |  | |  |  |  |  |  |  |  |  |  |  |  |
| AAEL001289 | 19.2726 | 4.56447 | -2.07803 | permease, putative [Source:VB External Description;Acc:AAEL001289] | | | | | | |  |  |  |  |  |  |
| AAEL007092 | 29.276 | 6.93459 | -2.07784 |  | |  |  |  |  |  |  |  |  |  |  |  |
| AAEL011519 | 6.24478 | 1.47926 | -2.07778 | sucrose transport protein [Source:VB External Description;Acc:AAEL011519] | | | | | | | |  |  |  |  |  |
| AAEL007111 | 2.31593 | 0.548984 | -2.07675 |  | |  |  |  |  |  |  |  |  |  |  |  |
| AAEL013267 | 5.86474 | 1.39053 | -2.07643 |  | |  |  |  |  |  |  |  |  |  |  |  |
| AAEL011492 | 22.4938 | 5.33699 | -2.07543 |  | |  |  |  |  |  |  |  |  |  |  |  |
| AAEL000206 | 35.0617 | 8.3207 | -2.07512 | ninjurin A, putative [Source:VB External Description;Acc:AAEL000206] | | | | | | |  |  |  |  |  |  |
| AAEL008658 | 8.10145 | 1.92395 | -2.07411 | leucine-rich immune protein (TM) [Source:VB Community Annotation;Acc:AAEL008658] | | | | | | | | |  |  |  |  |
| AAEL008600 | 12.5426 | 2.97919 | -2.07385 | zinc carboxypeptidase [Source:VB External Description;Acc:AAEL008600] | | | | | | | |  |  |  |  |  |
| AAEL017405 | 52.1377 | 12.3959 | -2.07246 |  | |  |  |  |  |  |  |  |  |  |  |  |
| AAEL010523 | 35.6343 | 8.47593 | -2.07182 |  | |  |  |  |  |  |  |  |  |  |  |  |
| AAEL000239 | 14.2066 | 3.37931 | -2.07176 |  | |  |  |  |  |  |  |  |  |  |  |  |
| AAEL000251 | 14.2066 | 3.37931 | -2.07176 |  | |  |  |  |  |  |  |  |  |  |  |  |
| AAEL002817 | 25.7624 | 6.13122 | -2.07102 |  | |  |  |  |  |  |  |  |  |  |  |  |
| AAEL003227 | 4.38141 | 1.04328 | -2.07026 | protease m1 zinc metalloprotease [Source:VB External Description;Acc:AAEL003227] | | | | | | | |  |  |  |  |  |
| AAEL011905 | 75.3918 | 17.9684 | -2.06894 | myosin i [Source:VB External Description;Acc:AAEL011905] | | | | | |  |  |  |  |  |  |  |
| AAEL006978 | 17.5623 | 4.18758 | -2.06829 | protein-glutamine gamma-glutamyltransferase [Source:VB External Description;Acc:AAEL006978] | | | | | | | | | |  |  |  |
| AAEL002257 | 4.06638 | 0.971673 | -2.0652 |  | |  |  |  |  |  |  |  |  |  |  |  |
| AAEL006973 | 9.55889 | 2.28443 | -2.06501 | ubiquitin conjugating enzyme 7 interacting protein [Source:VB External Description;Acc:AAEL006973] | | | | | | | | | |  |  |  |
| AAEL000455 | 7.38128 | 1.7642 | -2.06486 |  | |  |  |  |  |  |  |  |  |  |  |  |
| AAEL004825 | 5.80592 | 1.38798 | -2.06453 | potassium-dependent sodium-calcium exchanger, putative [Source:VB External Description;Acc:AAEL004825] | | | | | | | | | |  |  |  |
| AAEL001509 | 21.8199 | 5.21805 | -2.06406 |  | |  |  |  |  |  |  |  |  |  |  |  |
| AAEL001760 | 6.97974 | 1.66966 | -2.06362 |  | |  |  |  |  |  |  |  |  |  |  |  |
| AAEL002946 | 6.71687 | 1.60695 | -2.06346 |  | |  |  |  |  |  |  |  |  |  |  |  |
| AAEL007986 | 1881.17 | 450.182 | -2.06305 |  | |  |  |  |  |  |  |  |  |  |  |  |
| AAEL005733 | 223.141 | 53.5135 | -2.05998 | myosin heavy chain, nonmuscle or smooth muscle [Source:VB External Description;Acc:AAEL005733] | | | | | | | | | |  |  |  |
| AAEL001673 | 81.9582 | 19.6589 | -2.0597 | actin [Source:VB External Description;Acc:AAEL001673] | | | | | |  |  |  |  |  |  |  |
| AAEL014734 | 9.19168 | 2.20577 | -2.05904 |  | |  |  |  |  |  |  |  |  |  |  |  |
| AAEL017148 | 15.7148 | 3.77507 | -2.05755 |  | |  |  |  |  |  |  |  |  |  |  |  |
| AAEL009038 | 20.5023 | 4.92608 | -2.05727 | prolylcarboxypeptidase, putative [Source:VB External Description;Acc:AAEL009038] | | | | | | | |  |  |  |  |  |
| AAEL007659 | 22.0002 | 5.28635 | -2.05718 |  | |  |  |  |  |  |  |  |  |  |  |  |
| AAEL017352 | 72.4737 | 17.4159 | -2.05705 |  | |  |  |  |  |  |  |  |  |  |  |  |
| AAEL003843 | 115.833 | 27.8569 | -2.05594 |  | |  |  |  |  |  |  |  |  |  |  |  |
| AAEL006201 | 2.759 | 0.663629 | -2.0557 |  | |  |  |  |  |  |  |  |  |  |  |  |
| AAEL006860 | 5099.18 | 1227.27 | -2.05482 | 40S ribosomal protein S28 [Source:VB Community Annotation;Acc:AAEL006860] | | | | | | | |  |  |  |  |  |
| AAEL005868 | 51.0778 | 12.2967 | -2.05443 |  | |  |  |  |  |  |  |  |  |  |  |  |
| AAEL005353 | 13.5031 | 3.25295 | -2.05348 |  | |  |  |  |  |  |  |  |  |  |  |  |
| AAEL009993 | 13.8926 | 3.34681 | -2.05346 |  | |  |  |  |  |  |  |  |  |  |  |  |
| AAEL008471 | 3.65137 | 0.88023 | -2.05249 |  | |  |  |  |  |  |  |  |  |  |  |  |
| AAEL012352 | 26.0759 | 6.28923 | -2.05176 |  | |  |  |  |  |  |  |  |  |  |  |  |
| AAEL006702 | 123.135 | 29.7026 | -2.05157 | fibrinogen and fibronectin [Source:VB External Description;Acc:AAEL006702] | | | | | | | |  |  |  |  |  |
| AAEL010524 | 19.0076 | 4.58594 | -2.05128 |  | |  |  |  |  |  |  |  |  |  |  |  |
| AAEL000567 | 87.5049 | 21.1147 | -2.05111 | facilitated trehalose transporter Tret1 [Source:VB Community Annotation;Acc:AAEL000567] | | | | | | | | |  |  |  |  |
| AAEL004827 | 4.71752 | 1.13845 | -2.05096 |  | |  |  |  |  |  |  |  |  |  |  |  |
| AAEL015440 | 135.013 | 32.5967 | -2.0503 |  | |  |  |  |  |  |  |  |  |  |  |  |
| AAEL013069 | 3892.34 | 939.823 | -2.05018 | guanine nucleotide-binding protein subunit beta-like protein [Source:VB Community Annotation;Acc:AAEL013069] | | | | | | | | | | |  |  |
| AAEL013372 | 8.80125 | 2.12787 | -2.0483 | ABC transporter [Source:VB External Description;Acc:AAEL013372] | | | | | | |  |  |  |  |  |  |
| AAEL005293 | 7.53914 | 1.82416 | -2.04717 | galectin [Source:VB Community Annotation;Acc:AAEL005293] | | | | | | |  |  |  |  |  |  |
| AAEL001267 | 19.9127 | 4.81887 | -2.04692 |  | |  |  |  |  |  |  |  |  |  |  |  |
| AAEL001259 | 19.9127 | 4.81887 | -2.04692 |  | |  |  |  |  |  |  |  |  |  |  |  |
| AAEL002385 | 11.766 | 2.84758 | -2.04682 | Carboxy/choline esterase Alpha Esterase [Source:VB Community Annotation;Acc:AAEL002385] | | | | | | | | |  |  |  |  |
| AAEL017256 | 35.4227 | 8.5781 | -2.04594 |  | |  |  |  |  |  |  |  |  |  |  |  |
| AAEL004524 | 10.1875 | 2.46716 | -2.04588 | Clip-Domain Serine Protease family C. [Source:VB Community Annotation;Acc:AAEL004524] | | | | | | | | |  |  |  |  |
| AAEL008289 | 81.3526 | 19.7033 | -2.04575 | pupal cuticle protein 78E, putative [Source:VB External Description;Acc:AAEL008289] | | | | | | | | |  |  |  |  |
| AAEL003419 | 2.43422 | 0.589827 | -2.04509 |  | |  |  |  |  |  |  |  |  |  |  |  |
| AAEL006256 | 835.963 | 202.569 | -2.04503 | ATPase subunit, putative [Source:VB External Description;Acc:AAEL006256] | | | | | | | |  |  |  |  |  |
| AAEL011263 | 16.7875 | 4.06834 | -2.04487 | phosphatidylethanolamine-binding protein [Source:VB External Description;Acc:AAEL011263] | | | | | | | | |  |  |  |  |
| AAEL002267 | 30.7784 | 7.46051 | -2.04457 |  | |  |  |  |  |  |  |  |  |  |  |  |
| AAEL004156 | 8.93044 | 2.16511 | -2.04429 | fibrinogen and fibronectin [Source:VB External Description;Acc:AAEL004156] | | | | | | | |  |  |  |  |  |
| AAEL003596 | 186.075 | 45.1487 | -2.04313 |  | |  |  |  |  |  |  |  |  |  |  |  |
| AAEL008155 | 9.24783 | 2.24396 | -2.04307 | protease m1 zinc metalloprotease [Source:VB External Description;Acc:AAEL008155] | | | | | | | |  |  |  |  |  |
| AAEL008310 | 112.592 | 27.3332 | -2.04238 |  | |  |  |  |  |  |  |  |  |  |  |  |
| AAEL002534 | 7611.73 | 1848.71 | -2.04171 | 60S ribosomal protein L10 [Source:VB Community Annotation;Acc:AAEL002534] | | | | | | | |  |  |  |  |  |
| AAEL015251 | 18.3323 | 4.45354 | -2.04137 |  | |  |  |  |  |  |  |  |  |  |  |  |
| AAEL002656 | 4.86531 | 1.18312 | -2.03993 |  | |  |  |  |  |  |  |  |  |  |  |  |
| AAEL007910 | 9.02846 | 2.19662 | -2.03919 |  | |  |  |  |  |  |  |  |  |  |  |  |
| AAEL018333 | 3.79266 | 0.923774 | -2.0376 |  | |  |  |  |  |  |  |  |  |  |  |  |
| AAEL003426 | 2.47948 | 0.604256 | -2.03681 | sodium-dependent phosphate transporter [Source:VB External Description;Acc:AAEL003426] | | | | | | | | |  |  |  |  |
| AAEL004048 | 16.9152 | 4.12331 | -2.03644 | UNC93A protein, putative [Source:VB External Description;Acc:AAEL004048] | | | | | | | |  |  |  |  |  |
| AAEL000859 | 49.5917 | 12.0919 | -2.03606 |  | |  |  |  |  |  |  |  |  |  |  |  |
| AAEL007913 | 69.1454 | 16.8628 | -2.03579 | sulfotransferase (sult) [Source:VB External Description;Acc:AAEL007913] | | | | | | | |  |  |  |  |  |
| AAEL009112 | 29.102 | 7.09784 | -2.03566 |  | |  |  |  |  |  |  |  |  |  |  |  |
| AAEL006434 | 10.0763 | 2.45806 | -2.03537 | serine protease, putative [Source:VB External Description;Acc:AAEL006434] | | | | | | | |  |  |  |  |  |
| AAEL018653 | 15.1926 | 3.70664 | -2.03518 | 28S_rRNA [Source:RNAmmer;Acc:28S_rRNA] | | | | |  |  |  |  |  |  |  |  |
| AAEL017440 | 15.9939 | 3.90272 | -2.03497 |  | |  |  |  |  |  |  |  |  |  |  |  |
| AAEL010956 | 31.9343 | 7.81206 | -2.03133 |  | |  |  |  |  |  |  |  |  |  |  |  |
| AAEL001659 | 7.85039 | 1.92093 | -2.03096 | misexpression suppressor of ras, putative [Source:VB External Description;Acc:AAEL001659] | | | | | | | | |  |  |  |  |
| AAEL006443 | 2.85149 | 0.69816 | -2.03009 |  | |  |  |  |  |  |  |  |  |  |  |  |
| AAEL008118 | 2.80301 | 0.686419 | -2.02981 | Protein mab-21 [Source:VB External Description;Acc:AAEL008118] | | | | | | |  |  |  |  |  |  |
| AAEL009214 | 363.79 | 89.0892 | -2.02978 | diazepam binding inhibitor, putative [Source:VB External Description;Acc:AAEL009214] | | | | | | | | |  |  |  |  |
| AAEL000516 | 44.4263 | 10.8822 | -2.02945 | juvenile hormone-inducible protein, putative [Source:VB External Description;Acc:AAEL000516] | | | | | | | | |  |  |  |  |
| AAEL005331 | 238.363 | 58.4275 | -2.02844 |  | |  |  |  |  |  |  |  |  |  |  |  |
| AAEL009249 | 4.22122 | 1.03491 | -2.02815 | coronin [Source:VB External Description;Acc:AAEL009249] | | | | | |  |  |  |  |  |  |  |
| AAEL012509 | 15.6934 | 3.84906 | -2.02758 |  | |  |  |  |  |  |  |  |  |  |  |  |
| AAEL008016 | 5.11981 | 1.25645 | -2.02674 | short-chain dehydrogenase [Source:VB External Description;Acc:AAEL008016] | | | | | | | |  |  |  |  |  |
| AAEL017155 | 2.059 | 0.505584 | -2.02592 |  | |  |  |  |  |  |  |  |  |  |  |  |
| AAEL001181 | 4.07821 | 1.00203 | -2.025 |  | |  |  |  |  |  |  |  |  |  |  |  |
| AAEL006518 | 62.9464 | 15.4709 | -2.02457 | cytidine deaminase, putative [Source:VB External Description;Acc:AAEL006518] | | | | | | | |  |  |  |  |  |
| AAEL003641 | 20.5483 | 5.05138 | -2.02427 | sodium/chloride dependent amino acid transporter [Source:VB External Description;Acc:AAEL003641] | | | | | | | | | |  |  |  |
| AAEL000543 | 11.1061 | 2.73088 | -2.02391 | C-Type Lectin (CTLMA11) - mannose binding. [Source:VB Community Annotation;Acc:AAEL000543] | | | | | | | | | |  |  |  |
| AAEL001490 | 21.4805 | 5.28196 | -2.02388 | acylphosphatase, putative [Source:VB External Description;Acc:AAEL001490] | | | | | | | |  |  |  |  |  |
| AAEL013327 | 13.8706 | 3.41105 | -2.02375 |  | |  |  |  |  |  |  |  |  |  |  |  |
| AAEL016996 | 28.8779 | 7.10175 | -2.02372 |  | |  |  |  |  |  |  |  |  |  |  |  |
| AAEL008354 | 11.1509 | 2.74316 | -2.02324 | gaba receptor invertebrate [Source:VB External Description;Acc:AAEL008354] | | | | | | | |  |  |  |  |  |
| AAEL009271 | 1.71134 | 0.421121 | -2.02282 | testin [Source:VB External Description;Acc:AAEL009271] | | | | | |  |  |  |  |  |  |  |
| AAEL007824 | 11616.5 | 2858.81 | -2.02269 | 40S ribosomal protein S29 [Source:VB Community Annotation;Acc:AAEL007824] | | | | | | | |  |  |  |  |  |
| AAEL013432 | 7.36637 | 1.81409 | -2.02171 | serine protease, putative [Source:VB External Description;Acc:AAEL013432] | | | | | | | |  |  |  |  |  |
| AAEL003645 | 48.7921 | 12.0226 | -2.0209 | stearoyl-coa desaturase [Source:VB External Description;Acc:AAEL003645] | | | | | | | |  |  |  |  |  |
| AAEL014278 | 27.1358 | 6.68843 | -2.02046 |  | |  |  |  |  |  |  |  |  |  |  |  |
| AAEL006705 | 3.54306 | 0.873521 | -2.02008 |  | |  |  |  |  |  |  |  |  |  |  |  |
| AAEL008946 | 152.714 | 37.6597 | -2.01974 | acidic ribosomal protein P1, putative [Source:VB Community Annotation;Acc:AAEL008946] | | | | | | | | |  |  |  |  |
| AAEL004957 | 37.4523 | 9.23859 | -2.01931 |  | |  |  |  |  |  |  |  |  |  |  |  |
| AAEL003942 | 7858.86 | 1938.64 | -2.01927 | 60S ribosomal protein L44 L41, putative [Source:VB Community Annotation;Acc:AAEL003942] | | | | | | | | |  |  |  |  |
| AAEL000817 | 2.75791 | 0.680385 | -2.01915 | rhomboid [Source:VB External Description;Acc:AAEL000817] | | | | | |  |  |  |  |  |  |  |
| AAEL004680 | 8.71558 | 2.15227 | -2.01774 | nuclear lamin L1 alpha, putative [Source:VB External Description;Acc:AAEL004680] | | | | | | | |  |  |  |  |  |
| AAEL015665 | 22.0844 | 5.45369 | -2.01772 |  | |  |  |  |  |  |  |  |  |  |  |  |
| AAEL014620 | 25.0042 | 6.17559 | -2.01752 |  | |  |  |  |  |  |  |  |  |  |  |  |
| AAEL007038 | 7.87528 | 1.94545 | -2.01723 | prolyl 4-hydroxylase alpha subunit 1 [Source:VB External Description;Acc:AAEL007038] | | | | | | | | |  |  |  |  |
| AAEL004264 | 9.98273 | 2.4665 | -2.01697 |  | |  |  |  |  |  |  |  |  |  |  |  |
| AAEL007902 | 3.07379 | 0.759514 | -2.01687 |  | |  |  |  |  |  |  |  |  |  |  |  |
| AAEL008441 | 1247.23 | 308.238 | -2.01662 |  | |  |  |  |  |  |  |  |  |  |  |  |
| AAEL008444 | 1247.23 | 308.238 | -2.01662 |  | |  |  |  |  |  |  |  |  |  |  |  |
| AAEL005749 | 7.05136 | 1.74281 | -2.01649 | lysosomal alpha-mannosidase (mannosidase alpha class 2b member 1) [Source:VB External Description;Acc:AAEL005749] | | | | | | | | | | |  |  |
| AAEL004470 | 144.112 | 35.629 | -2.01607 |  | |  |  |  |  |  |  |  |  |  |  |  |
| AAEL007388 | 3.84093 | 0.950057 | -2.01537 |  | |  |  |  |  |  |  |  |  |  |  |  |
| AAEL013517 | 11.4608 | 2.83496 | -2.0153 | pupal cuticle protein 78E, putative [Source:VB External Description;Acc:AAEL013517] | | | | | | | | |  |  |  |  |
| AAEL012149 | 12.3533 | 3.05696 | -2.01473 | npdc-1 [Source:VB External Description;Acc:AAEL012149] | | | | | |  |  |  |  |  |  |  |
| AAEL017180 | 7.99587 | 1.97886 | -2.01458 |  | |  |  |  |  |  |  |  |  |  |  |  |
| AAEL009992 | 9.77819 | 2.4261 | -2.01093 |  | |  |  |  |  |  |  |  |  |  |  |  |
| AAEL017030 | 125.281 | 31.0864 | -2.01081 |  | |  |  |  |  |  |  |  |  |  |  |  |
| AAEL014966 | 27.2948 | 6.77502 | -2.01033 |  | |  |  |  |  |  |  |  |  |  |  |  |
| AAEL005187 | 6.50274 | 1.61595 | -2.00867 | laminin gamma 1 chain [Source:VB External Description;Acc:AAEL005187] | | | | | | | |  |  |  |  |  |
| AAEL003889 | 14.4267 | 3.58559 | -2.00846 | Gram-Negative Binding Protein (GNBP) or Beta-1 3-Glucan Binding Protein (BGBP). [Source:VB Community Annotation;Acc:AAEL003889] | | | | | | | | | | | | |
| AAEL015151 | 24.9879 | 6.21763 | -2.00679 | acid phosphatase [Source:VB External Description;Acc:AAEL015151] | | | | | | |  |  |  |  |  |  |
| AAEL005220 | 507.381 | 126.442 | -2.0046 | 60S ribosomal protein L30 [Source:VB Community Annotation;Acc:AAEL005220] | | | | | | | |  |  |  |  |  |
| AAEL005660 | 5.85061 | 1.45813 | -2.00447 | p60 epidermal growth factor receptor, putative [Source:VB External Description;Acc:AAEL005660] | | | | | | | | |  |  |  |  |
| AAEL008478 | 11.6117 | 2.89502 | -2.00393 |  | |  |  |  |  |  |  |  |  |  |  |  |
| AAEL001467 | 2.20846 | 0.550703 | -2.00369 | sdk-P1 [Source:VB External Description;Acc:AAEL001467] | | | | | |  |  |  |  |  |  |  |
| AAEL008323 | 5.80556 | 1.44949 | -2.00189 |  | |  |  |  |  |  |  |  |  |  |  |  |
