# Supplementary material for: The Lethal(2)-Essential-for-Life [L(2)EFL] Gene Family Modulates Dengue Virus Infection in Aedes aegypti
Source: Int J Mol Sci. 2020 Oct 12;21(20):7520. doi: 10.3390/ijms21207520 (PMC7593908; doi:10.3390/ijms21207520)
Supplement: Supplementary file 1 [file ijms-21-07520-s001.zip › Supplementary Data/Tables S1,2,3.docx]

Table S1: List of primer sequences for dsRNA production

|  |
| --- |

*l(2)efl-1* (AAEL013338)

First round PCR:

Forward primer: 5’-CGACTCACTATAGGGTTGCTCCCCGTCAAGACATC- 3’

Reverse primer: 5’-CGACTCACTATAGGGTTGACCGGTCTGGACGATTG- 3’

Second round PCR (T7): 5’-ATAGAATTCTCTAGAAGCTTAATACGACTCACTATAGGG- 3’

*l(2)efl-2* (AAEL013346)

First round PCR:

Forward primer: 5’-CGACTCACTATAGGGGGACCAGCATTTTGGCAGTG- 3’

Reverse primer: 5’-CGACTCACTATAGGGGAAGGTTCTTAGCTGGTTGCC- 3’

*l(2)efl-3* (AAEL010670)

First round PCR:

Forward primer: 5’-CGACTCACTATAGGGGCATTGCAGAATCAGAGACGC- 3’

Reverse primer: 5’-CGACTCACTATAGGGATCCTGCAGTTGTTTCGCTG- 3’

*l(2)efl-4* (AAEL013351)

First round PCR:

Forward primer: 5’-CGACTCACTATAGGGATTTTCACGGCCCTGGTTCA- 3’

Reverse primer: 5’-CGACTCACTATAGGGATCCGCTTCAGGTTTCTCGG- 3’

*l(2)efl-5* (AAEL013344)

First round PCR:

Forward primer: 5’-CGACTCACTATAGGGAACACGCATTCGATAAAGTGATTTC- 3’

Reverse primer: 5’-CGACTCACTATAGGGTTCTCCTCGTGCTTGCCTTC- 3’

|  |
| --- |

Table S2: List of primer sequences for RNA-seq validation

|  |
| --- |

Histone H4 (AAEL003689)

Forward primer: 5’-AACACCTTCAGTACGCCTCG- 3’

Reverse primer: 5’-GGTATAACGAAGCCCGCCAT- 3’

*l(2)efl-1* (AAEL013338)

Forward primer: 5’-AGCCTTGCTCCCCGTCAAGA- 3’

Reverse primer: 5’-GTCAGAGGCCTTCACCGAAA- 3’

4-nitrophenyl phosphatase (AAEL007097)

Forward primer: 5’-GCAGTACACGGTCCTAGATGG- 3’

Reverse primer: 5’-ATATCCAAAACGACCGCACCA- 3’

Cdc6 (AAEL010855)

Forward primer: 5’-AACCTCGACTGAACCGTGTC- 3’

Reverse primer: 5’-AGGACGTATCGCTGAGGACA- 3’

Cactus (AAEL000709)

Forward primer: 5’-GGCCGCCATCCAACACAACC- 3’

Reverse primer: 5’-GGAAAGGCCAGGGAAAGTGGC- 3’

Antibacterial peptide (AAEL004223)

Forward primer: 5’-GAAGCTGGTCGGCTGAAGAA- 3’

Reverse primer: 5’-AACCGTCTCCACCGACAATC- 3’

Hypothetical protein (AAEL004851)

Forward primer: 5’-GTTCCCGCCGTATGAATTTGG- 3’

Reverse primer: 5’-GCTTCAGTCGCTTCACCGAA- 3’

Vago (AAEL000200)

Forward primer: 5’-GATCGCAGTAGCATTTGCCG- 3’

Reverse primer: 5’-ACACTCCGGGTAATCCTTGG- 3’

Zinc carboxylpeptidase (AAEL001863)

Forward primer: 5’-GCTCCAAAGGACCAGCGATC- 3’

Reverse primer: 5’-GAGGTTCGTTGTAAGGGGCA- 3’

Carboxylpeptidase (AAEL010776)

Forward primer: 5’-GTATCGTTTCGCTAAGGAAGCA- 3’

Reverse primer: 5’-ATCGTTGAAGGGCAAGATTCAAG- 3’

40s rps17 (AAEL004175)

Forward primer: 5’-GGCCATCATTCCAACGAAGC- 3’

Reverse primer: 5’-ACCAGGACCAGGACGTGATA- 3’

|  |
| --- |

Table S3: List of primer sequences for *l(2)efl-2* to *l(2)efl-5*

|  |
| --- |

*l(2)efl-2* (AAEL013346)

Forward primer: 5’-CACGCATACGAAAACGTGGT- 3’

Reverse primer: 5’-TCGTTTGCACAGAACACTTG- 3’

*l(2)efl-3* (AAEL010670)

Forward primer: 5’-CACGCATTCGATAAAGTGATTGT- 3’

Reverse primer: 5’-ACGATCGGAATGAGCGACAT- 3’

*l(2)efl-4* (AAEL013351)

Forward primer: 5’-TGGTGGAATACCTCGCTTCG- 3’

Reverse primer: 5’-TTGAACCAGGGCCGTGAAAA- 3’

*l(2)efl-5* (AAEL013344)

Forward primer: 5’-CCTCTGCGTACATCCCAACTC- 3’

Reverse primer: 5’-GGAGCGTCTCTGATTCTGTAAC- 3’

|  |
| --- |
