# Supplementary material for: The Lethal(2)-Essential-for-Life [L(2)EFL] Gene Family Modulates Dengue Virus Infection in Aedes aegypti
Source: Int J Mol Sci. 2020 Oct 12;21(20):7520. doi: 10.3390/ijms21207520 (PMC7593908; doi:10.3390/ijms21207520)
Supplement: Supplementary file 1 [file ijms-21-07520-s001.zip › Supplementary Data/Figure S1.pptx]

## Slide 1
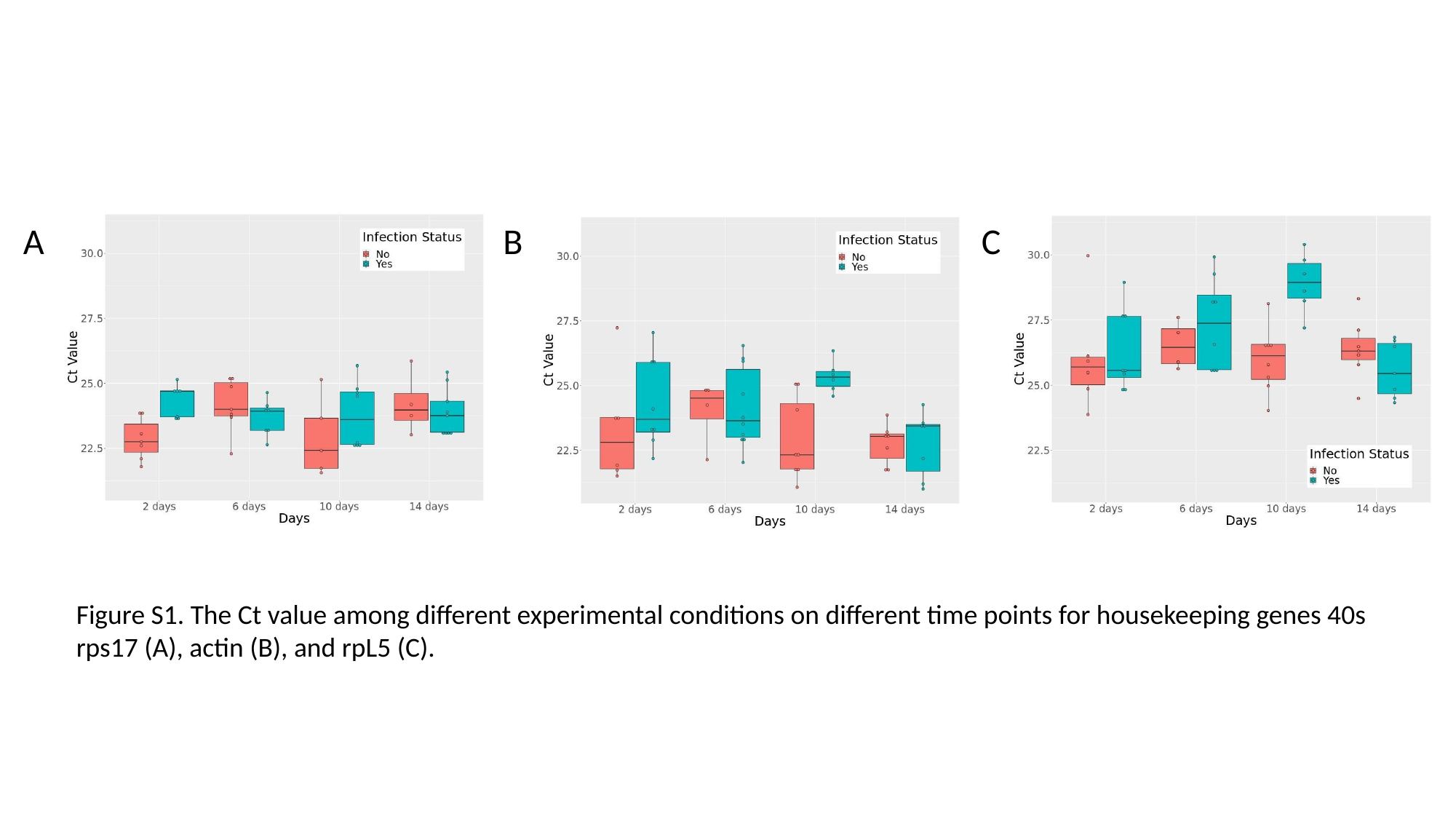

C
B
A
Figure S1. The Ct value among different experimental conditions on different time points for housekeeping genes 40s rps17 (A), actin (B), and rpL5 (C).
